# Light-assisted carbon dioxide reduction in an automated photoreactor system coupled to carbonylation chemistry†

**DOI:** 10.1039/d4sc06660j

**Published:** 2024-11-08

**Authors:** Jasper H. A. Schuurmans, Tom M. Masson, Stefan D. A. Zondag, Simone Pilon, Nicola Bragato, Miguel Claros, Tim den Hartog, Francesc Sastre, Jonathan van den Ham, Pascal Buskens, Giulia Fiorani, Timothy Noël

**Affiliations:** a Flow Chemistry Group, Van't Hoff Institute for Molecular Sciences (HIMS), Universiteit van Amsterdam (UvA) 1098 XH Amsterdam The Netherlands t.noel@uva.nl; b Dipartimento di Scienze Molecolari e Nanosistemi, Università Ca' Foscari Venezia Via Torino 155 30172 Venezia Italy; c Zuyd University of Applied Sciences Nieuw Eyckholt 300 6419 DJ Heerlen The Netherlands; d The Netherlands Organisation for Applied Scientific Research (TNO) High Tech Campus 25 5656 AE Eindhoven The Netherlands; e Design and Synthesis of Inorganic Materials (DESINe), Institute for Materials Research, Hasselt University Agoralaan Building D 3590 Diepenbeek Belgium

## Abstract

Continuous-flow methodologies offer promising avenues for sustainable processing due to their precise process control, scalability, and efficient heat and mass transfer. The small dimensions of continuous-flow reactors render them highly suitable for light-assisted reactions, as can be encountered in carbon dioxide hydrogenations. In this study, we present a reactor system emphasizing reproducibility, modularity, and automation, facilitating streamlined screening of conditions and catalysts for these processes. The proposed commercially available photoreactor, in which carbon dioxide hydrogenation was conducted, features narrow channels with a high-surface area catalyst deposition. Meticulous control over temperature, light intensity, pressure, residence time, and reagent stoichiometry yielded the selective formation of carbon monoxide and methane using heterogeneous catalysts, including a novel variant of ruthenium nanoparticles on titania catalyst. All details on the automation are made available, enabling its use by researchers worldwide. Furthermore, we demonstrated the direct utilization of on-demand generated carbon monoxide in the production of fine chemicals *via* various carbonylative cross-coupling reactions.

## Abstract 2

A modular, automated photoreactor system for CO_2_ hydrogenation enables precise conversion into methane or carbon monoxide. The carbon monoxide is subsequently utilized in carbonylation reactions, advancing sustainable synthesis and CO_2_ utilization.

## Graphical abstract

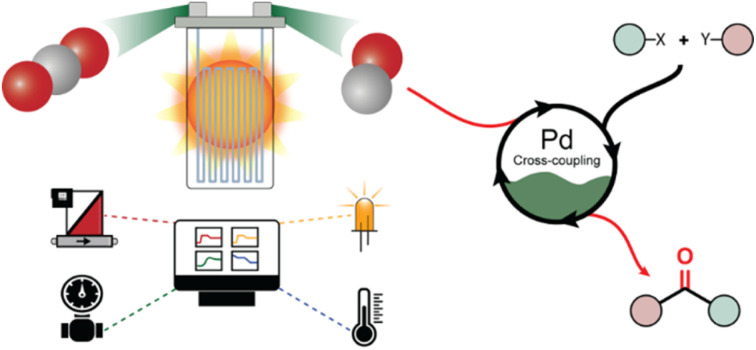

## Introduction

The rising concentrations of greenhouse gases in our atmosphere, with carbon dioxide as a notorious example, have propelled sustainable processing and carbon capture and utilization (CCU) to the forefront of both academic and industrial research efforts.^1–5^ Tackling the carbon footprint challenge necessitates a collaborative endeavor across various disciplines. Chemistry and chemical engineering, guided by the principles of green chemistry, take on a pivotal role in advancing this overarching goal.^6,7^ This entails a fundamental shift away from conventional fossil fuel-based methodologies towards sustainable alternatives, exemplified by the emergence of photochemical processes and circular chemistry practices.^8,9^ These approaches leverage milder conditions and, where feasible, harness solar energy as a green and renewable driving force for reactions.^10–14^ Integrating solar-powered or -assisted techniques with continuous-flow technologies offers enhanced control, scalability, selectivity, as well as superior mass and heat transfer capabilities to processes.^15–18^

Although carbon dioxide represents an ideal C1 building block, its inert nature has limited widespread application in synthetic organic chemistry. Arguably, a more promising approach involves converting CO_2_ into diverse C1 building blocks like methane or carbon monoxide.^19^ Particularly, carbon monoxide serves as an activated C1 building block with abundant associated chemistry. Reduction of CO_2_ can be achieved through hydrogenation reactions, such as the Sabatier reaction and reverse water gas shift (RWGS) reaction. These reactions have also been investigated as light-assisted processes in photochemical or photothermal systems,^20–22^ thereby lowering energy consumption and enhancing sustainability.^23–25^

To date, significant strides have been made in the development and evaluation of catalysts and reactor systems for the hydrogenation of carbon dioxide.^26–34^ A variety of photoreactor systems have been proposed to carry out these reactions without the presence of any liquid, usually revolving around non-refined catalyst deposition on a support without surface area optimization, and one-sided heating. An increase in surface area and the suppression of short-circuiting require additional design considerations, where packed bed, monolith or optical fiber reactors are potential candidates.^22,35–38^ The reactor's applicability to catalyst screening is primarily determined by its versatility for adaption to various systems and operational flexibility. An additional challenge arises from the preferential use of narrow (micro)channels, which are favored for their good heat transfer properties and short diffusion lengths.^15,39^ However, their small dimensions make it challenging to deposit a fine catalyst powder, often leading to excessive pressure drops.^40,41^ Moreover, the direct comparison of the performance and efficacy of different catalysts remains challenging due to the absence of standardized operational procedures. While standardization methodologies have been effectively employed in batch processes for high-throughput screening and continuous-flow liquid systems, their application to light-assisted gas-phase reactions over heterogeneous catalysts remains relatively unexplored in this context.^42–47^

In this endeavor, we aim to tackle these challenges by introducing an automated, integrated, and modular reactor system, using an oil tank for homogeneous heating. We propose a straightforward catalyst immobilization strategy to maximize surface area, flexibility and reproducibility, while at the same time minimizing the pressure drop.^48–50^ The standardized reactor system is equipped with automated control over crucial parameters such as temperature, light intensity, pressure, residence time, and reagent stoichiometry, enabling the execution of gas-phase chemistry in a robust and reproducible manner. The automatization uses either commercially available, benchmarked equipment or inexpensive in-house developed devices and control, facilitated by a custom-built, open-source code, accessible for use in any facility worldwide. The precise control and modularity of this system facilitate the continuous light-assisted production of carbon dioxide hydrogenation products, which can be seamlessly integrated into downstream units. In this study, we leverage the generated carbon monoxide as a reagent in a diverse array of palladium-catalyzed carbonylative cross-coupling reactions, demonstrating the versatility and utility of our approach.

## Results and discussion

### Design of the automated, modular continuous-flow photoreactor setup

**Fig. 1:**
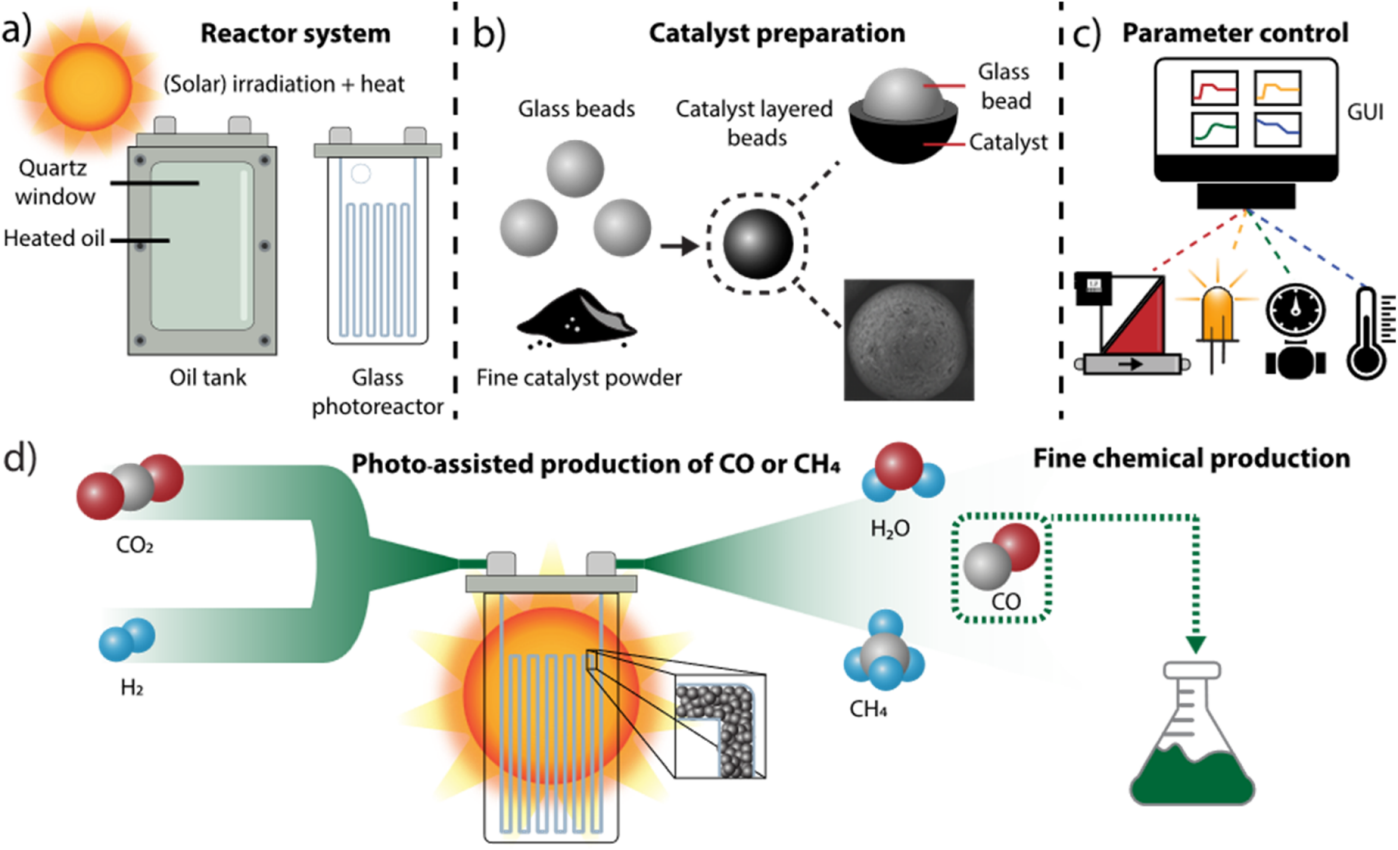
(a) Principles of the modular system, including the heating and the photoreactor. (b) Schematic representation of the catalyst loading on glass beads, with a cross section and SEM image. (c) Overview of the automated control system for the individual flow rate, light intensity, pressure and temperature. (d) Scheme for the production of fine chemicals from carbon dioxide and hydrogen.

We initiated our research by developing a reactor assembly tailored to evaluating catalysts for high-temperature light-driven processes, particularly relevant for light-assisted carbon dioxide reduction.^20,51,52^ This assembly features a heated stainless steel tank with a quartz window for irradiation, which can house glass microchannel photoreactors. A transparent silicon oil was employed to ensure efficient and uniform heat transfer between the tank and the reactor. The reflective properties of stainless steel, combined with the high refractive index of the oil and quartz, optimize photon utilization within the reactor unit. Moreover, the oil-filled tank offers versatility by having the possibility of accommodating various reactor types, resulting in a highly modular system (Fig. 1a). Our investigation primarily focused on commercially available microchannel photoreactors, well-suited for continuous-flow reactions within our experimental framework.^53,54^ These reactors were filled with an immobilized catalyst, where the catalyst powders were easily and uniformly coated onto glass beads (Fig. 1b, ESI Sections S1 and S5†), creating a packed-bed system with precise amounts of active catalyst.^50,55^ This configuration facilitated a large irradiation area and ensured reproducible packing, enhancing the reliability of our experimental setup.

Utilizing automation to precisely regulate and adjust reaction parameters aligns with the modularity and continuous-flow nature of our system. The labor-intensive process of screening various conditions and benchmarking catalysts can be streamlined into a reproducible and automated procedure. All monitored reaction parameters are under electronic control, coordinated by a computer interface that enables users to modify and monitor each parameter as needed. The flow rate of individual reagents is precisely controlled by mass flow controllers (MFCs), while the temperature of the silicon oil is maintained by a proportional–integral–derivative (PID) controller connected to a heating element and thermocouple within the oil tank. Reaction pressure is monitored and regulated by a sensor at the reactor outlet, with adjustment facilitated by a mechanical back pressure regulator (BPR). To enable electronic pressure control, a closed-loop system was established between the pressure sensor and a motor controlling the BPR. Furthermore, the intensity of the light emitting diode (LED) light source is adjusted through an electronically controlled LED power supply. Both pressure and light control devices were custom-designed and manufactured using cost-effective electronic components and sensors, and are operated by Arduino microcontrollers (UNO boards). Overall, centralized control over reagent stoichiometry, residence time, oil temperature, pressure, and light intensity is managed by a Python script running on a dedicated computer (Fig. 1c). An intuitive graphical user interface (GUI) has been developed to facilitate automation of reaction conditions, as well as real-time adjustment and monitoring of all parameters, without requiring advanced scripting knowledge. This control code, along with detailed construction specifications and firmware for the in-house developed devices, is provided as open-source software, facilitating systematic experiment reproducibility and adaptation for similar automation setups.

To assess the efficacy of each catalyst across diverse reaction conditions, the gas mixture exiting the reactor is analyzed using an online gas chromatograph equipped with both a thermal conductivity detector and a flame ionization detector. The automated system was deployed to optimize the hydrogenation of carbon dioxide, targeting a range of hydrogenation products, including carbon monoxide. This compound can be generated *ex situ* and directly utilized as a feedstock in carbonylative cross-coupling reactions, relevant for the synthesis of various fine chemicals, pharmaceuticals and agrochemicals (Fig. 1d).^56,57^ The benefits of the system were leveraged in the parametric screening, catalyst selection and operation for subsequent reactions.

### Light-assisted hydrogenation of carbon dioxide

**Fig. 2:**
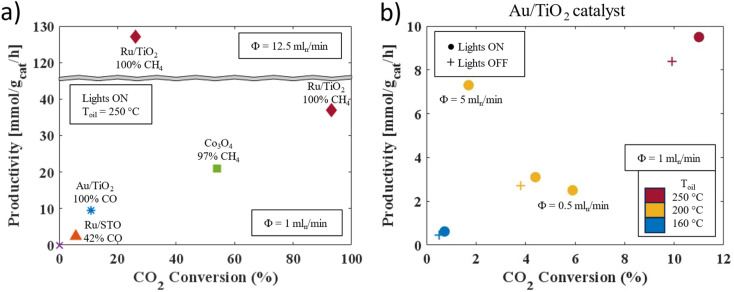
(a) An overview of the evaluated catalysts in the hydrogenation of carbon dioxide, presenting the total carbon dioxide conversion against the productivity towards the desired compound (at steady-state). The overall selectivity is given in the data labels. The point in the origin represents the control experiment in which no hydrogenation products where observed. The feed ratio between CO_2_ : H_2_ is equal to 1 : 4 for reactions targeting methane and 1 : 1 for reactions targeting carbon monoxide. (b) Results of the parametric screening of the gold nanoparticles on titanium dioxide (Au/TiO_2_) in the formation of carbon monoxide.

The platform's versatility was demonstrated through the light-assisted hydrogenation of carbon dioxide, where this stable compound was transformed into gaseous reduction products, such as methane and carbon monoxide, using heat and light. To comprehensively assess the impact of key variables, we conducted systematic experiments across a range of heterogeneous catalysts (see ESI Section S6† for details). Among these catalysts, commercially available cobalt(ii,iii) oxide (Co_3_O_4_) was selected as a benchmark, providing a well-established reference for comparative analysis with other catalysts.^58^Fig. 2a illustrates the selectivity, conversion, and productivity of the reaction under various operational conditions. Notably, the cobalt oxide catalyst exhibited remarkable selectivity towards methane production, with its formation increasing with rising reaction temperatures.

However, this benchmark catalyst was surpassed by another, namely ruthenium nanoparticles on titanium dioxide (Ru/TiO_2_, details on the characterization are available in ESI Section S3†), achieving nearly complete conversion with 100% selectivity towards methane. This novel catalyst was synthesized by introducing RuCl_3_ into an aqueous dispersion of TiO_2_ P90, a commercially available blend of rutile and anatase. This process led to the formation and deposition of ruthenium(iii) oxide hydroxide species onto the TiO_2_ surface, which were subsequently reduced to ruthenium nanoparticles. Analysis by inductively coupled plasma–atomic emission spectroscopy revealed a ruthenium content of 5.5% w/w, corresponding to a ruthenium yield of 96%. Transmission electron microscopy examination demonstrated a uniform distribution of spherical ruthenium nanoparticles on the TiO_2_ surface, with an average particle size of 1.79 ± 0.51 nm (lognormal distribution). Notably, the productivity of this catalyst could be further enhanced to 127 mmol g_cat_^−1^ h^−1^ by increasing the flow rate.

Control experiments confirmed that the targeted compounds were exclusively generated from the catalyst-mediated transformation of the feedstock (see ESI Section S6.2† for details). Furthermore, the versatility and adaptability of our screening platform were underscored by seamlessly transitioning to alternative catalyst systems. For instance, a catalyst comprising ruthenium (oxide) nanoparticles supported on strontium titanate (STO, Ru/STO) exhibited varying selectivity in carbon monoxide production (approximately ranging between 40% and 70%), depending on the specific reaction conditions and duration of operation. Previous studies had reported high methane selectivity for a similar catalyst, suggesting that the observed variance could be attributed to the catalyst's preparation and exposure to distinct reaction environments.^59^ Contrarily, another catalyst consisting of gold nanoparticles dispersed on titanium dioxide (Au/TiO_2_ 3.1% w/w gold on anatase, with spheroidal gold particles averaging 1.70 nm in diameter, lognormal distribution) consistently yielded carbon monoxide as the primary product across all investigated conditions. This catalyst achieved productivities of up to 9.5 mmol g_cat_^−1^ h^−1^, showcasing its robust performance under diverse reaction conditions. The carbon dioxide conversions obtained with catalysts targeting carbon monoxide were generally lower than those with methane producing catalysts, as illustrated in Fig. 2a. This could be attributed to the relatively low temperatures employed, combined with the endothermic nature of the reverse water gas shift reaction.^60,61^

Precise automated control over the oil temperature, light intensity, and individual flow rates facilitated systematic condition screening for all investigated catalysts, with selected results depicted in Fig. 2b (all results can be found in the ESI Section S6.1†). The irradiation of the reactor system notably enhanced carbon monoxide production when employing the Au/TiO_2_ catalyst. However, the flow rate exhibited a dual influence, with productivity and conversion showing a trade-off relationship, likely attributable to decreased residence time at higher throughputs. Furthermore, the reaction temperature emerged as a critical determinant of both conversion and productivity. Particularly noteworthy is the observation that significant carbon monoxide production only occurred at temperatures exceeding 160 °C, underscoring the necessity for thermal heating or robust light sources to supply the requisite thermal energy for catalyst-driven reactions. In general, a comparable dependency of the investigated parameters on methane production was observed for the Ru/TiO_2_ catalyst. Remarkably, this catalyst maintained a methane production of 3.5 mmol g_cat_^−1^ h^−1^ at 160 °C, highlighting its potential to produce value-added compounds under these conditions. The parametric screening process furnishes invaluable insights into optimizing reaction conditions to meet predefined criteria, like purity or throughput, thereby enabling on-demand production of carbon monoxide or methane from carbon dioxide.

### On-demand supply of carbon monoxide in carbonylation reactions

**Fig. 3:**
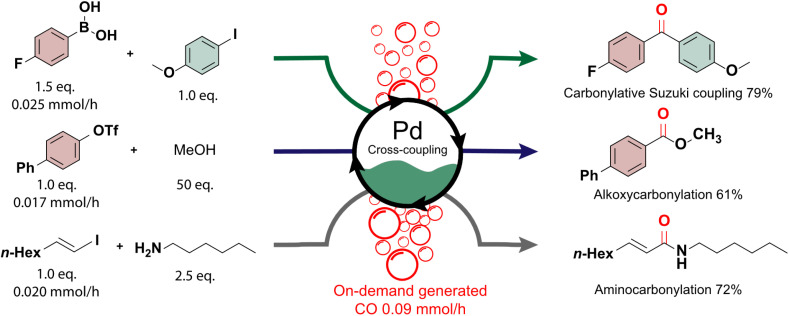
Carbon monoxide generated by the photoreactor is subsequently used as a reagent in the (top) carbonylative Suzuki coupling (7.5 mol% of Pd(OAc)_2_ and xantphos, 3 eq. of K_2_CO_3_, in dioxane : THF (after addition, 98 : 2 volume ratio), addition over 6 hours, reaction for 7 hours at 80 °C), (middle) an alkoxycarbonylation (5 mol% of Pd(OAc)_2_ and dppp, 3 eq. of TEA, in dioxane, addition over 6 hours, reaction for 6.5 hours at 70 °C) and (bottom) an aminocarbonylation reaction (10 mol% of Pd_2_(dba)_3_ and tpp, 3 eq. of K_2_CO_3_, in dioxane, addition over 5 hours, reaction for 5.5 hours at 80 °C).

The generated carbon monoxide serves as a crucial precursor in subsequent reactions, showcasing the seamless integration of our light-assisted process with synthetically valuable cross-coupling reactions.^62^ This integration was demonstrated through the successful execution of carbonylative Suzuki coupling, alkoxycarbonylation, and aminocarbonylation reactions using on-demand carbon monoxide directly from our automated photoreactor platform (Fig. 3). Carbonylation reactions hold particular significance due to the versatility imparted by the carbonyl functional group, a key constituent of numerous biologically active molecules and pharmaceuticals.^63–65^ Traditionally, achieving selective carbonylation reactions necessitates high pressures and temperatures.^66–68^ However, the reactions conducted in this study proved compatible with the continuous supply of carbon monoxide, requiring only low pressures and moderate temperatures. This underscores the potential of our carbon dioxide upgrading approach to facilitate efficient and sustainable synthesis routes for diverse carbonylation reactions.

The initial conditions for the experiments were established in batch for the palladium-catalyzed Suzuki coupling between 4-iodoanisole, carbon monoxide and 4-fluorophenylboronic acid. Various headspace compositions were analyzed to optimize the reaction and evaluate its compatibility with hydrogen and carbon dioxide, present in the mixture (see ESI Section S7†). A decrease in reactivity was noted, possibly due to the formation of an inactive palladium precipitate in the presence of hydrogen.^69,70^ To address this, a combination of Pd(OAc)_2_ and XantPhos was employed. For the carbonylative Suzuki coupling, a fed-batch approach was adopted, with the boronic acid added dropwise to the reactor flask while continuously purging the photoreactor's gaseous outlet through the reaction mixture. Similarly, in alkoxycarbonylation and aminocarbonylation, the limiting reagents ([1,1′-biphenyl]-4-yl trifluoromethanesulfonate and (*E*)-1-iodooct-1-ene, respectively) were continuously added to the reaction medium to maintain their presence throughout the reaction (see ESI Section S1.2†). This strategy resulted in excellent selectivity towards the desired carbonylated products, facilitated by the low relative concentration of the added reagent and the continuous replenishment of carbon monoxide. The products of the carbonylative Suzuki coupling, alkoxycarbonylation, and aminocarbonylation reactions were isolated in good yields (79%, 61%, and 72%, respectively). These findings highlight the adaptability of fine chemical production to specific carbon dioxide sources, with potential for scalability through a numbering-up or sizing-up strategy to adjust the carbon monoxide production accordingly.^71,72^

## Outlook & conclusion

In this work, we introduced a modular and automated photoreactor system designed to facilitate the light-assisted hydrogenation of carbon dioxide. The integration of automation and modularity standardized the screening of diverse catalysts and reaction conditions. Employing a packed-bed approach, wherein the catalyst is deposited on glass beads, offered several advantages, including providing extensive irradiation areas, ensuring reproducible loading, and optimizing catalyst utilization.

The platform's versatility enables the evaluation of novel catalysts under tightly controlled reaction parameters, addressing a critical need in the scientific community for a standardized system to assess and compare catalysts in both thermal and light-assisted heterogeneous-catalyzed gas conversion processes. The production of one of the most encountered reduction products, namely methane, by a commercially available cobalt(ii,iii) oxide catalyst, was used to validate the system.^22,73,74^ Moreover, the automated system enables temperature-controlled, light-assisted reactions, facilitating swift mechanistic studies on photothermal reaction pathways.^75^ Leveraging commercially available components alongside open-source code establishes a framework for future advancements in automation within the field.

Furthermore, the integrated flow system allows for seamless integration of downstream units, enabling immediate utilization of generated products from carbon dioxide. The methane produced to validate the system can serve as building block for fine chemical synthesis.^76,77^ However, direct methane activation is challenging due to its high bond dissociation energy, other reduction products like carbon monoxide create possibilities for carbonylations.^19,78^ This integration is exemplified by the on-demand production of carbon monoxide for various carbonylative cross-coupling reactions. Tailoring the catalyst and operation conditions enabled stable carbon monoxide production required for these purposes.

In conclusion, our modular and automated photoreactor system not only advances the understanding and exploration of light-assisted gas conversion processes but also lays the groundwork for future developments in automation within the field, offering a promising avenue for sustainable chemical synthesis.

## Methods

### Modular reactor system

A glass photoreactor (LTF MR-LAB-V) was immersed into a heated oil reactor casing with a quartz window. The backplate of the casing contained ceramic heating rods to heat the oil (CAS 68083-14-7) to a set temperature using a PID controller. The pressure was regulated with an automated back pressure regulator developed in-house. An overflow tube and vessel are present to prevent pressure buildup in the tank. Irradiation was provided by an array of four white chip-on-board LEDs (CXB3590, cool white, 86 W electrical input power per chip) placed at 2 cm from the window. Gases were supplied through mass flow controllers (MFC, Bronkhorst max. 10 ml min^−1^, volume at normal conditions; 0 °C and 1 atm), connected to a supply at 5 bars, and could be co-fed into the reactor. Stainless-steel Swagelok and IDEX fittings in combination with PFA/FEP tubing (OD 1.6 mm) were used to connect the distinct parts. The analysis was conducted with a gas chromatograph (see ESI Section S2†).

### Ruthenium on titanium dioxide catalyst

The Ru/TiO_2_ catalyst was synthesized by a direct chemical reduction method, RuCl_3_ (Ruthenium(iii) chloride hydrate, 99.9% (PGM basis), Alfa Aesar) was used as the ruthenium precursor. A 6 mM RuCl_3_ solution was prepared with 100 ml ultra-filtered water (Milli-Q Millipore, 18.2 MΩ cm) in vigorous stirring for 5–10 minutes in a three necked round bottom flask. The support TiO_2_ (1.00 g, AEROXIDE TiO_2_ P90 Evonik) was added to the mixture. The dispersion was left in vigorous stirring for at least 1 hour at 100 °C. A 0.18 M NaBH_4_ solution was prepared using demineralized water. 10 ml of this solution was added to the previous mixture using a pump with a flow rate of 3 ml min^−1^ and the mixture was left stirring for an extra 30 minutes. The solid was recuperated by filtration and extensively washed with ultra-filtered water. Further characterization details can be found in the ESI Section S3.†

### CO_2_ hydrogenation

The catalyst was deposited on glass beads (150–212 μm) following a previously published procedure,^79^ prior to the placement in the glass reactor. A layer of the catalyst was obtained on the glass beads by first grinding the catalyst, followed by mixing with the glass beads. Approximately 1.5 grams of coated beads were deposited in the glass reactor (1.0 wt% of catalyst). The catalysts were supplied by partners, (produced based on literature^30,80^), apart from commercially available Co_3_O_4_ (Sigma-Aldrich). The desired parameters for the reaction could be set in the graphical user interface. Samples were taken after steady-state was reached.

### Carbonylation reactions in batch

Determined amounts of the reagents, catalyst and additives were charged in a Schlenk flask (under nitrogen). Before heating, the headspace of the flask was filled with a specific ratio of gases (using a balloon and autoclave, see ESI Section S1.2†). Hereafter, the Schlenk flask was immersed in an oil bath to meet the set temperature. Reactions were performed under continuous stirring. Qualitative and quantitative analysis and characterization of reaction mixtures and pure products were performed with GC-MS and NMR.

### Carbonylation reactions with on-demand produced carbon monoxide

Determined amounts of the reagents, catalyst and additives were charged in a Schlenk flask (under nitrogen). Before heating, gas from the automated system was purged through the solution, to fill the headspace with the gas mixture. Hereafter, the Schlenk flask was immersed in an oil bath to meet the set temperature. One of the reagents in solution was supplied by a syringe pump at a specific flow rate. Reactions were performed under continuous stirring. Qualitative and quantitative analysis and characterization of reaction mixtures and pure products were performed with GC-MS and NMR.

## Data availability

The data supporting this article have been included as part of the ESI.† The GUI, automation and custom devices control software is available through GitHub: https://github.com/Noel-Research-Group/automation-for-solar-assisted-carbon-dioxide-hydrogenations.

## Author contributions

The roles as defined by CRediT for this work are as follows: conceptualization (J. S., T. M., S. Z., T. N.), data curation (J. S., T. M., S. Z.), funding acquisition (T. N.), investigation (J. S., T. M., S. Z., N. B., M. C., F. S., J. H.), methodology (J. S., T. M., S. Z., S. P., N. B., F. S., J. H.), project administration (T. N.), software (S. P.), supervision (M. C., T. H., P. B., G. F., T. N.), writing – original draft (J. S., T. M., S. Z., S. P.), writing – review & editing (M. C., T. H., P. B., G. F., T. N.).

## Conflicts of interest

The authors declare no conflict of interest.

## Supplementary Material

SC-015-D4SC06660J-s001

## References

[cit1] Choi Y. H. (2017). *et al.*, Carbon dioxide Fischer-Tropsch synthesis: A new path to carbon-neutral fuels. Appl. Catal., B.

[cit2] Lozano F. J. (2018). *et al.*, New perspectives for green and sustainable chemistry and engineering: Approaches from sustainable resource and energy use, management, and transformation. J. Cleaner Prod..

[cit3] Baena-Moreno F. M. (2019). *et al.*, Carbon capture and utilization technologies: a literature review and recent advances. Energy Sources, Part A.

[cit4] Detz R. J., van der Zwaan B. (2019). Transitioning towards negative CO_2_ emissions. Energy Policy.

[cit5] Masoudi Soltani S. (2021). *et al.*, Sorption-enhanced Steam Methane Reforming for Combined CO_2_ Capture and Hydrogen Production: A State-of-the-Art Review. Carbon Capture Sci. Technol..

[cit6] Anastas P., Eghbali N. (2010). Green Chemistry: Principles and Practice. Chem. Soc. Rev..

[cit7] Mulvihill M. J., Beach E. S., Zimmerman J. B., Anastas P. T. (2011). Green Chemistry and Green Engineering: A Framework for Sustainable Technology Development. Annu. Rev. Environ. Resour..

[cit8] Yoon T. P., Ischay M. A., Du J. (2010). Visible light photocatalysis as a greener approach to photochemical synthesis. Nat. Chem..

[cit9] Keijer T., Bakker V., Slootweg J. C. (2019). Circular chemistry to enable a circular economy. Nat. Chem..

[cit10] Ravelli D., Dondi D., Fagnoni M., Albini A. (2009). Photocatalysis. A multi-faceted concept for green chemistry. Chem. Soc. Rev..

[cit11] Crisenza G. E. M., Melchiorre P. (2020). Chemistry glows green with photoredox catalysis. Nat. Commun..

[cit12] Masson T. M. (2021). *et al.*, Development of an Off-Grid Solar-Powered Autonomous Chemical Mini-Plant for Producing Fine Chemicals. ChemSusChem.

[cit13] Zondag S. D. A., Masson T. M., Debije M. G., Noël T. (2022). The development of luminescent solar concentrator-based photomicroreactors: a cheap reactor enabling efficient solar-powered photochemistry. Photochem. Photobiol. Sci..

[cit14] Zondag S. D. A. (2024). *et al.*, Determining photon flux and effective optical path length in intensified flow photoreactors. Nat. Chem. Eng..

[cit15] Gemoets H. P. L. (2016). *et al.*, Liquid phase oxidation chemistry in continuous-flow microreactors. Chem. Soc. Rev..

[cit16] Dallinger D., Kappe C. O. (2017). Why flow means green – Evaluating the merits of continuous processing in the context of sustainability. Curr. Opin. Green Sustainable Chem..

[cit17] Buglioni L., Raymenants F., Slattery A., Zondag S. D. A., Noël T. (2022). Technological Innovations in Photochemistry for Organic Synthesis: Flow Chemistry, High-Throughput Experimentation, Scale-up, and Photoelectrochemistry. Chem. Rev..

[cit18] Laporte A. A. H., Masson T. M., Zondag S. D. A., Noël T. (2024). Multiphasic Continuous-Flow Reactors for Handling Gaseous Reagents in Organic Synthesis: Enhancing Efficiency and Safety in Chemical Processes. Angew. Chem., Int. Ed..

[cit19] Raymenants F., Masson T. M., Sanjosé-Orduna J., Noël T. (2023). Efficient C(sp^3^)−H Carbonylation of Light and Heavy Hydrocarbons with Carbon Monoxide via Hydrogen Atom Transfer Photocatalysis in Flow. Angew. Chem., Int. Ed..

[cit20] Ghoussoub M., Xia M., Duchesne P. N., Segal D., Ozin G. (2019). Principles of photothermal gas-phase heterogeneous CO_2_ catalysis. Energy Environ. Sci..

[cit21] Wang S. (2021). *et al.*, CO_2_ Footprint of Thermal Versus Photothermal CO_2_ Catalysis. Small.

[cit22] Schuurmans J. H. A., Masson T. M., Zondag S. D. A., Buskens P., Noël T. (2024). Solar-Driven Continuous CO_2_ Reduction
to CO and CH_4_ using Heterogeneous Photothermal Catalysts: Recent Progress and Remaining Challenges. ChemSusChem.

[cit23] Stankiewicz A. I., Moulijn J. A. (2000). Process intensification: Transforming chemical engineering. Chem. Eng. Prog..

[cit24] Centi G., Perathoner S. (2010). Towards Solar Fuels from Water and CO_2_. ChemSusChem.

[cit25] Xavier Silva C. (2024). *et al.*, Techno-economic analysis for the sunlight-powered reverse water gas shift process: Scenarios, costs, and comparative insights. Sustain. Energy Technol. Assessments.

[cit26] Sastre F. (2019). *et al.*, Sunlight-Fueled, Low-Temperature Ru-Catalyzed Conversion of CO_2_ and H_2_ to CH_4_ with a High Photon-to-Methane Efficiency. ACS Omega.

[cit27] Khan A. A., Tahir M. (2019). Recent advancements in engineering approach towards design of photo-reactors for selective photocatalytic CO_2_ reduction to renewable fuels. J. CO_2_ Util..

[cit28] Dong Y. (2020). *et al.*, Shining light on CO_2_: from materials discovery to photocatalyst, photoreactor and process engineering. Chem. Soc. Rev..

[cit29] Grote R. (2020). *et al.*, Collective photothermal effect of Al_2_O_3_ -supported spheroidal plasmonic Ru nanoparticle catalysts in the sunlight-powered Sabatier reaction. ChemCatChem.

[cit30] Martínez Molina P. (2021). *et al.*, Low Temperature Sunlight-Powered Reduction of CO_2_ to CO Using a Plasmonic Au/TiO_2_ Nanocatalyst. ChemCatChem.

[cit31] Volders J. (2022). *et al.*, Sunlight-Powered Reverse Water Gas Shift Reaction Catalysed by Plasmonic Au/TiO_2_ Nanocatalysts: Effects of Au Particle Size on the Activity and Selectivity. Nanomaterials.

[cit32] Ozin G. (2022). Accelerated optochemical engineering solutions to CO_2_ photocatalysis for a sustainable future. Matter.

[cit33] Rohlfs J. (2022). *et al.*, Continuous-Flow Sunlight-Powered CO_2_ Methanation Catalyzed by γ-Al_2_O_3_-Supported Plasmonic Ru Nanorods. Catalysts.

[cit34] Martínez Molina P. (2023). *et al.*, Sunlight Powered Continuous Flow Reverse Water Gas Shift Process Using a Plasmonic Au/TiO_2_ Nanocatalyst. Chem.–Asian J..

[cit35] Nguyen T.-V., Wu J. C. S. (2008). Photoreduction of CO_2_ to fuels under sunlight using optical-fiber reactor. Sol. Energy Mater. Sol. Cells.

[cit36] Liou P.-Y. (2011). *et al.*, Photocatalytic CO_2_ reduction using an internally illuminated monolith photoreactor. Energy Environ. Sci..

[cit37] Amara Z. (2015). *et al.*, Applying green chemistry to the photochemical route to artemisinin. Nat. Chem..

[cit38] Toson P., Doshi P., Jajcevic D. (2019). Explicit Residence Time Distribution of a Generalised Cascade of Continuous Stirred Tank Reactors for a Description of Short Recirculation Time (Bypassing). Processes.

[cit39] Tanimu A., Jaenicke S., Alhooshani K. (2017). Heterogeneous catalysis in continuous flow microreactors: A review of methods and applications. Chem. Eng. J..

[cit40] Faridkhou A., Tourvieille J.-N., Larachi F. (2016). Reactions, hydrodynamics and mass transfer in micro-packed beds—Overview and new mass transfer data. Chem. Eng. Process..

[cit41] Ali H., Ahmed I., Robertson K., Lanterna A. E. (2024). PDI-Functionalized Glass Beads: Efficient, Metal-Free Heterogeneous Photocatalysts Suitable for Flow Photochemistry. Org. Process Res. Dev..

[cit42] Mayr L. M., Bojanic D. (2009). Novel trends in high-throughput screening. Curr. Opin. Pharmacol..

[cit43] Plutschack M. B., Pieber B., Gilmore K., Seeberger P. H. (2017). The Hitchhiker's Guide to Flow Chemistry. Chem. Rev..

[cit44] Waldron C. (2019). *et al.*, An autonomous microreactor platform for the rapid identification of kinetic models. React. Chem. Eng..

[cit45] Sahm C. D., Ucoski G. M., Roy S., Reisner E. (2021). Automated and Continuous-Flow Platform to Analyze Semiconductor–Metal Complex Hybrid Systems for Photocatalytic CO_2_ Reduction. ACS Catal..

[cit46] Hurtado L. (2022). *et al.*, Solar CO_2_ hydrogenation by photocatalytic foams. Chem. Eng. J..

[cit47] Slattery A. (2024). *et al.*, Automated self-optimization, intensification, and scale-up of photocatalysis in flow. Science.

[cit48] Knobloch C., Güttel R., Turek T. (2013). Holdup and Pressure Drop in Micro Packed-Bed Reactors for Fischer-Tropsch Synthesis. Chem. Ing. Tech..

[cit49] Lin G., Qiu H. (2022). Diverse Supports for Immobilization of Catalysts in Continuous Flow Reactors. Chem.–Eur. J..

[cit50] Gesmundo N. J., Tu N. P., Sarris K. A., Wang Y. (2023). ChemBeads-Enabled Photoredox High-Throughput Experimentation Platform to Improve C(sp^2^)–C(sp^3^) Decarboxylative Couplings. ACS Med. Chem. Lett..

[cit51] Aquilanti V., Mundim K. C., Elango M., Kleijn S., Kasai T. (2010). Temperature dependence of chemical and biophysical rate processes: Phenomenological approach to deviations from Arrhenius law. Chem. Phys. Lett..

[cit52] Scaiano J. C. (2023). A beginners guide to understanding the mechanisms of photochemical reactions: things you should know if light is one of your reagents. Chem. Soc. Rev..

[cit53] Gobert S. R. L., Kuhn S., Braeken L., Thomassen L. C. J. (2017). Characterization of Milli- and Microflow Reactors: Mixing Efficiency and Residence Time Distribution. Org. Process Res. Dev..

[cit54] Masson T. M., Zondag S. D. A., Debije M. G., Noël T. (2022). Rapid and Replaceable Luminescent Coating for Silicon-Based Microreactors Enabling Energy-Efficient Solar Photochemistry. ACS Sustain. Chem. Eng..

[cit55] Tu N. P. (2019). *et al.*, High-Throughput Reaction Screening with Nanomoles of Solid Reagents Coated on Glass Beads. Angew. Chem., Int. Ed..

[cit56] Xia Y.-S. (2022). *et al.*, Tandem utilization of CO_2_ photoreduction products for the carbonylation of aryl iodides. Nat. Commun..

[cit57] Sang R. (2022). *et al.*, A practical concept for catalytic carbonylations using carbon dioxide. Nat. Commun..

[cit58] Chen G. (2018). *et al.*, Alumina-Supported CoFe Alloy Catalysts Derived from Layered-Double-Hydroxide Nanosheets for Efficient Photothermal CO_2_ Hydrogenation to Hydrocarbons. Adv. Mater..

[cit59] Mateo D., Albero J., García H. (2019). Titanium-Perovskite-Supported RuO_2_ Nanoparticles for Photocatalytic CO_2_ Methanation. Joule.

[cit60] Chen X. (2020). *et al.*, Recent Advances in Supported Metal Catalysts and Oxide Catalysts for the Reverse Water-Gas Shift Reaction. Front. Chem..

[cit61] Yu J., Muhetaer A., Li Q., Xu D. (2024). Solar Energy-Driven Reverse Water Gas Shift Reaction: Photothermal Effect, Photoelectric Activation and Selectivity Regulation. Small.

[cit62] Fukuyama T., Totoki T., Ryu I. (2014). Carbonylation in microflow: close encounters of CO and reactive species. Green Chem..

[cit63] Suzuki Y. J., Carini M., Butterfield D. A. (2010). Protein Carbonylation. Antioxid. Redox Signaling.

[cit64] Wu X., Neumann H., Beller M. (2013). Palladium-Catalyzed Oxidative Carbonylation Reactions. ChemSusChem.

[cit65] Gadge S. T., Bhanage B. M. (2014). Recent developments in palladium catalysed carbonylation reactions. RSC Adv..

[cit66] Littke A. F., Dai C., Fu G. C. (2000). Versatile Catalysts for the Suzuki Cross-Coupling of Arylboronic Acids with Aryl and Vinyl Halides and Triflates under Mild Conditions. J. Am. Chem. Soc..

[cit67] Wu X.-F. (2014). *et al.*, Transition-Metal-Catalyzed Carbonylation Reactions of Olefins and Alkynes: A Personal Account. Acc. Chem. Res..

[cit68] Liu Y., Chen Y.-H., Yi H., Lei A. (2022). An Update on Oxidative C–H Carbonylation with CO. ACS Catal..

[cit69] Iwasawa T., Tokunaga M., Obora Y., Tsuji Y. (2004). Homogeneous Palladium Catalyst Suppressing Pd Black Formation in Air Oxidation of Alcohols. J. Am. Chem. Soc..

[cit70] Bunge M. (2010). *et al.*, Formation of palladium(0) nanoparticles at microbial surfaces. Biotechnol. Bioeng..

[cit71] Su Y., Kuijpers K., Hessel V., Noël T. (2016). A convenient numbering-up strategy for the scale-up of gas–liquid photoredox catalysis in flow. React. Chem. Eng..

[cit72] Zondag S. D. A., Mazzarella D., Noël T. (2023). Scale-Up of Photochemical Reactions: Transitioning from Lab Scale to Industrial Production. Annu. Rev. Chem. Biomol. Eng..

[cit73] Ashok J. (2020). *et al.*, A review of recent catalyst advances in CO_2_ methanation processes. Catal. Today.

[cit74] Akpasi S. O., Isa Y. M. (2022). Review of Carbon Capture and Methane Production from Carbon Dioxide. Atmosphere.

[cit75] Baffou G., Bordacchini I., Baldi A., Quidant R. (2020). Simple experimental procedures to distinguish photothermal from hot-carrier processes in plasmonics. Light: Sci. Appl..

[cit76] Laudadio G. (2020). *et al.*, C(sp^3^)–H functionalizations of light hydrocarbons using decatungstate photocatalysis in flow. Science.

[cit77] Nagornîi D., Raymenants F., Kaplaneris N., Noël T. C. (2024). sp^3^)–H sulfinylation of light hydrocarbons with sulfur dioxide via hydrogen atom transfer photocatalysis in flow. Nat. Commun..

[cit78] Pulcinella A. (2024). *et al.*, C1-4 Alkylation of Aryl Bromides with Light Alkanes enabled by Metallaphotocatalysis in Flow. Angew. Chem., Int. Ed..

[cit79] Impastato A. C., Brown J. T. C., Wang Y., Tu N. P. (2023). Readily Accessible High-Throughput Experimentation: A General Protocol for the Preparation of ChemBeads and EnzyBeads. ACS Med. Chem. Lett..

[cit80] Peng Y., Albero J., Franconetti A., Concepción P., García H. (2022). Visible and NIR Light Assistance of the N_2_ Reduction to NH_3_ Catalyzed by Cs-promoted Ru Nanoparticles Supported on Strontium Titanate. ACS Catal..

