# Supplementary material for: Light-assisted carbon dioxide reduction in an automated photoreactor system coupled to carbonylation chemistry
Source: Chem Sci. 2024 Nov 8;15(47):19842–50. doi: 10.1039/d4sc06660j (PMC11575595; doi:10.1039/d4sc06660j)
Supplement: SC-015-D4SC06660J-s001 [file SC-015-D4SC06660J-s001.pdf]

Supplementary Information (SI) for Chemical Science.
This journal is © The Royal Society of Chemistry 2024

Light-assisted carbon dioxide reduction in an automated photoreactor system coupled to carbonylation chemistry

Supplementary Information

Jasper H.A. Schuurmans†,[1] Tom M. Masson†,[1] Stefan D.A. Zondag†,[1], Simone Pilon,[1] Nicola Bragato,[1,2] Miguel Claros,[1] Tim den Hartog,[3,4] Francesc Sastre,[4] Jonathan van den Ham,[4] Pascal Buskens,[4,5] Giulia Fiorani,[2] Timothy Noël* [1]

[1] Flow Chemistry Group, van't Hoff Institute for Molecular Sciences (HIMS), Universiteit van Amsterdam (UvA), 1098 XH Amsterdam, The Netherlands.

[2] Dipartimento di Scienze Molecolari e Nanosistemi, Università Ca' Foscari Venezia, Via Torino 155, 30172 Venezia, Italy

[3] Zuyd University of Applied Sciences, Nieuw Eyckholt 300, 6419 DJ Heerlen, The Netherlands.

[4] The Netherlands Organisation for Applied Scientific Research (TNO), High Tech Campus 25, 5656 AE Eindhoven, The Netherlands.

[5] Design and Synthesis of Inorganic Materials (DESINe), Institute for Materials Research, Hasselt University, Agoralaan Building D, 3590 Diepenbeek, Belgium.

† These authors contributed equally to the work.

* Corresponding Author: t.noel@uva.nl (Timothy Noël)

Contents

S1 Experimental 3

S1.1 CO_2 hydrogenation catalyst preparation and loading 3

S1.2 Carbonylation reactions 3

S2 Gas chromatography 8

S2.1 Analysis 8

S2.2 Correction for non-equimolar reactions 9

S3 Catalyst characterization 12

S4 Reactor system 19

S4.1 Design 19

S4.2 Silicon oil 19

S4.3 Light source 20

S4.4 Temperature control 20

S4.5 Pressure drop 22

S5 Automation 23

S5.1 System Overview 23

S5.2 Mass flow controllers 23

S5.3 Temperature controller 24

S5.4 Light intensity controller 24

S5.5 Back pressure regulator 28

S5.6 System automation monitor 33

S6 Additional results CO and CH_4 production 36

S6.1 Catalyst screening 36

S6.2 Control experiments 37

S6.3 Catalyst performance in literature 38

S6.4 Catalyst deactivation 38

S7 Additional results carbonylative cross-coupling 39

S7.1 Carbonylative Suzuki coupling 39

S7.2 Alkoxycarbonylation 39

S7.3 Aminocarbonylation 40

S8 References 41

S9 Characterization data 42

S10 NMR and Mass Spectra 43

S1 Experimental

S1.1 CO_2 hydrogenation catalyst preparation and loading

Glass beads (150-212 µm) were weighed and put in a glass sample vial. The catalyst was weighed and grinded with a mortar until it was a fine and homogeneous powder. This powder was added to a vial containing glass beads. A vibrating mixer was taped on the sample vial and turned on, resulting in mixing of the glass beads and catalyst (Figure S1a). The mixer was removed after 5 minutes, obtaining an even distribution of the catalyst on the beads, as can be seen in Figure S1b. The prepared catalyst-coated beads were loaded into the photoreactor through a funnel, followed by overpressure generation using pressurized gas (nitrogen or compressed air) to move it through the channels. The outlet was blocked with a particle filter to prevent loss of the material. The bed remained fixed during reactor operation, as the overpressure within the experimental operating window proved insufficient to result in bed movement (maximum of 4 bar overpressure). Additionally, no leaching of the coated catalyst was observed for the gas phase reactions. For replacement of the catalyst after deactivation or possible reactor contamination, the glass beads are easily flushed out of the reactor using a flow of liquid under sonication. After drying of the reactor, a new batch of coated glass beads can be loaded, and the reactor can subsequently be re-inserted into the reactor holder and casing (Figure S1c).

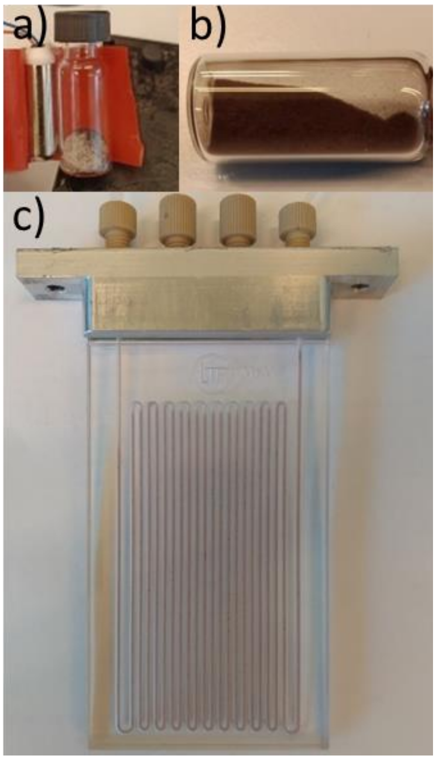

Figure S1 a) Vibrating mixer taped to the glass vial with the catalyst and glass beads. b) Evenly dispersed catalyst on the glass beads. c) Catalyst-coated beads loaded in the glass photoreactor.

S1.2 Carbonylation reactions

General

All reagents and solvents used: ethyl acetate (EtOAc), dioxane, dichloromethane, pentane, tetrahydrofuran (THF), methanol, fluorobenzene, 4-iodoanisole, 4-fluorophenylboronic acid, *trans*-1-iodo-1-octene, 1-hexylamine, 4-phenylphenol, pyridine, trifluoromethanesulfonic anhydride, palladium(II)bromide ($PdBr_2$), palladium(II)acetate [$Pd(OAc)_2$], tris(dibenzylideneacetone)palladium(0) [$Pd_2(dba)_3$], triphenylphosphine (tpp), 1,3-bis(diphenylphosphino)propane (dppp), 4,5-bis(diphenylphosphino)-9,9-dimethylxanthene

(Xantphos), triethylamine (TEA), K_2CO_3, Na_2SO_4, chloroform-d ($CDCl_3$), were purchased from Sigma Aldrich (now Merck) with ≥ 98 % purity, and, unless otherwise specified, used without further purification. Carbon monoxide gas (4.8 purity) was purchased from Nippon gases. [1,1'-biphenyl]-4-yl trifluoromethanesulfonate was prepared according to reported procedures.[1] Product isolation was performed by a Biotage® Isolera Four, with Biotage® SNAP KP-Sil 4 or 10 g flash chromatography cartridge, or manually, using silica (P60, SILICYCLE). TLC analysis was performed using silica on aluminum foils TLC plates (F254, SILICYCLE) with visualization under ultraviolet light (254 nm and 365 nm) or appropriate TLC staining (potassium permanganate).

Qualitative and quantitative analysis and characterization of reaction mixtures and pure products were performed with GC-MS and NMR. The GC-MS system used was an Agilent 8890 GC equipped with an HP-5MS UI capillary column (30 m·0.25 mm, film thickness: 0.25 µm), coupled with Agilent Technologies 5977C mass detector operating at 70 eV. Helium was used as carrier gas and an injection temperature of 300 °C was employed, using a temperature program of 60 °C (3.5 min) – 300 °C (3 min) at 30 °C/min. For NMR analysis, an AV 300-I NMR spectrometer was used, operating at 300 MHz for ^{1}H nuclei, 75 MHz for ^{13}C nuclei and 282 MHz for ^{19}F nuclei. ^{1}H NMR spectra are reported in parts per million (ppm) downfield relative to $CDCl_3$ (7.26 ppm) and all ^{13}C NMR spectra are reported in ppm relative to $CDCl_3$ (77.16 ppm) unless stated otherwise. The multiplicities of signals are designated by the following abbreviations: s (singlet), d (doublet), t (triplet), q (quartet), p (pentet), sext (sextet), m (multiplet), dd (doublet of doublets), dt (doublet of triplets), td (triplet of doublets), ddd (doublet of doublet of doublets). Coupling constants (J) are reported in hertz (Hz). NMR data was processed using the MestReNova 14 software package. Known products were characterized by comparing to the corresponding ^{1}H NMR, ^{13}C NMR and ^{19}F NMR with those available in the literature.

Carbonylative Suzuki coupling in batch mode

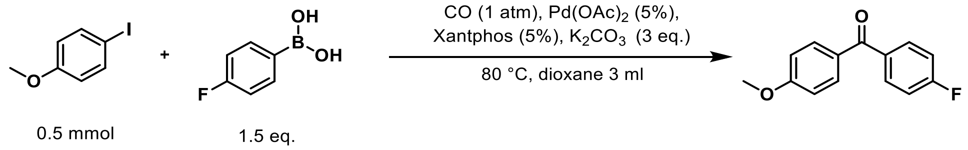

In a 10 ml Schlenk flask equipped with a stirring bar, were introduced 105 mg of 4-fluorophenyl boronic acid (0.75 mmol, 1.5 eq.), 5.6 mg of $Pd(OAc)_2$ (0.025 mmol, 5 mol%), 14.5 mg of 4,5-bis(diphenylphosphino)-9,9-dimethylxanthene (Xantphos, 0.025 mmol, 5 mol%) and 205 mg of anhydrous K_2CO_3 (1.5 mmol, 3.0 eq.). The system was capped with a septum and put under nitrogen through 3 vacuum/N_2 cycles before the addition of 3 ml of anhydrous dioxane and 117 mg of 4-iodoanisole (0.5 mmol, 1.0 eq.). The Schlenk flask was purged with a balloon containing CO or the gas mixture (volumetric ratio of 1:9 CO:H_2) during 5 minutes before introducing it into a preheated oil bath at 80 °C. The reaction was allowed to stir continuously under these conditions for 2 hours. After the reaction, the Schlenk flask was cooled down to room temperature and purged with N_2, before the introduction of 47 µl (0.5 mmol, 1 eq.) of fluorobenzene as standard for the analysis. The system, used for these and other reactions in batch, is illustrated in Figure S2a.

Carbonylative Suzuki coupling in fed-batch mode

In a 10 ml Schlenk flask equipped with a stirring bar, were introduced 5.6 mg of $Pd(OAc)_2$ (0.025 mmol, 5 mol%), 14.5 mg of Xantphos (0.025 mmol, 5 mol%) and 205 mg of K_2CO_3 (1.5 mmol, 3.0 eq.). The system was capped with a septum and put under nitrogen through 3 vacuum/N_2 cycles before the addition of 2 ml of anhydrous dioxane and 117 mg of 4-Iodoanisole (0.5 mmol, 1.0 eq.). Separately, in a 5 ml vial equipped with a septum cap, 105 mg of 4-fluorophenyl boronic acid (0.75 mmol, 1.5 eq.), were dissolved in 1 ml of anhydrous dioxane:THF mixture (95:5) under nitrogen atmosphere. The Schlenk flask was purged with a balloon containing CO or a gas mixture (volumetric ratio of 1:9 CO:H_2) during 1 minute before introducing it into a preheated oil bath at 80°C. The reaction was allowed to stir

continuously under these conditions during the addition of the 4-fluorophenyl boronic acid solution with the help of a syringe pump (Chemyx Fusion 200, corresponding flow rate in Table S13), and then left until the corresponding reaction time. After the reaction, the Schlenk flask was cooled down to room temperature and purged with N_2, before the introduction of 47 µl (0.5 mmol, 1.0 eq.) of fluorobenzene as standard for the analysis. The fed batch system is shown in Figure S2b.

Alkoxycarbonylation in batch mode

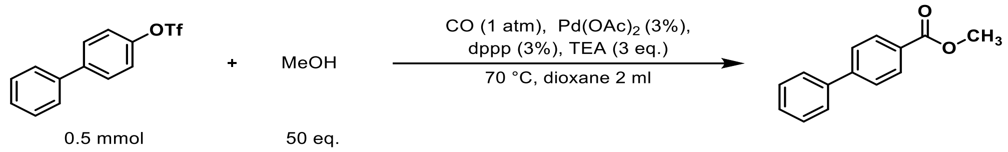

In a 10 ml Schlenk flask equipped with a stirring bar, 151 mg of [1,1'-biphenyl]-4-yl trifluoromethanesulfonate (0.5 mmol, 1.0 eq.), 3.4 mg of $Pd(OAc)_2$ (0.015 mmol, 3 mol%) and 6.2 mg of 1,3-bis(diphenylphosphino)propane (dppp, 0.015 mmol, 3 mol%) were introduced. The system was capped with a septum and put under nitrogen through 3 vacuum/N_2 cycles before the addition of 2 ml of anhydrous dioxane, 1 ml of methanol (25 mmol, 50 eq.) and 209 µl triethylamine (TEA, 1.5 mmol, 3.0 eq) under nitrogen flow. The Schlenk flask was purged with a balloon containing CO or a gas mixture (volumetric ratio of 1:9 CO:H_2) during 1 minute before introducing it into a preheated oil bath at 70 °C. The reaction was allowed to stir continuously under these conditions for 3 hours. After the reaction, the Schlenk flask was cooled down to room temperature and purged with N_2, before analysis.

Aminocarbonylation in batch mode

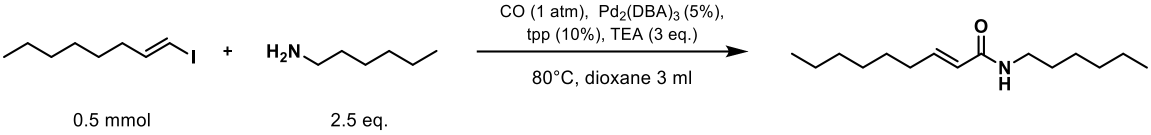

In a 10 ml Schlenk flask equipped with a stirring bar, 22.9 mg of $Pd_2(dba)_3$ (0.025 mmol, 5 mol%), 13.1 mg of triphenylphosphine (tpp, 0.05 mmol, 10 mol%) were introduced. The system was capped with a septum and put under nitrogen through 3 vacuum/N_2 cycles before the addition of 3 ml of anhydrous dioxane followed by 119 mg of (*E*)-1-iodooct-1-ene (0.5 mmol, 1.0 eq.), 165 µl of 1-hexylamine (1.25 mmol, 2.5 eq.) and 209 µl of triethylamine (TEA, 1.5 mmol, 3.0 eq.) under nitrogen flow. The Schlenk flask was purged with a balloon containing CO during 1 minute before introducing it into a preheated oil bath at 80 °C. The reaction was allowed to stir continuously under these conditions for 3 hours. After the reaction, the Schlenk flask was cooled down to room temperature and purged with N_2, before analysis.

Gas mixture preparation

For the preparation of CO and H_2 gas mixtures a stainless steel autoclave equipped with an electronic manometer with a capacity of 952 ml was used. The autoclave was first degassed through 3 vacuum/N_2 cycles and left under vacuum. Then, the autoclave was charged with H_2 followed by CO reaching a total pressure of 5 bar. The gaseous mixture was collected in a balloon which was subsequently used to carry out the carbonylation reaction.

On-demand produced carbon monoxide used in the carbonylative Suzuki coupling

In a 10 ml Schlenk flask, equipped with stirring bar (X shape) 23.4 mg of 4-iodoanisole (0.1 mmol, 1.0 eq.), 1.7 mg of $Pd(OAc)_2$ (0.0075 mmol, 7.5 mol%), 4.3 mg of 4,5-bis(diphenylphosphino)-9,9-dimethylxanthene (Xantphos, 0.0075 mmol, 7.5 mol%) and 41.4 mg of anhydrous K_2CO_3 (0.15 mmol, 3.0 eq.) were introduced. The system is capped with a septum and put under inert environment through 3 vacuum/N_2 cycles. Separately, in a 5 ml vial with a septum cap, 32.2 mg of 4-fluorophenylboronic

acid (0.23 mmol) was dissolved in 1.5 ml of a mixture of anhydrous dioxane:THF (95:5). The solution was sonicated and purged with N_2 to ensure complete solubilization of the boronic acid and deoxygenation of the mixture. Then, 0.5 ml of anhydrous dioxane was added to the 10 ml Schlenk flask under continuous nitrogen flow. The system is then purged with the photoreactor output for 30 minutes with continuous stirring at room temperature. In the meanwhile, a 3 ml syringe (ø = 9.5 mm) was charged with the boronic acid solution. The syringe is mounted on the syringe pump and a flow rate of 0.0028 ml/min was set to drop in 1 ml (0.15 mmol, 1.5 eq.) of the boronic acid solution. Once all preparations are complete, the Schlenk reactor was put inside to the oil bath (previously set at 80°C) with the beginning of the addition of the boronic acid solution via the syringe pump (see Figure S2c). At the end of the 6 hours of addition, the mixture is left to react for an additional hour after which the mixture is let to cool down to room temperature and then flushed with nitrogen to remove the CO present inside the reactor. 9.4 µl (0.1 mmol, 1 eq.) of fluorobenzene was introduced as standard for the analysis. The reaction mixture was extracted with EtOAc/water, and the organic layer was subsequently dried over Na_2SO_4. After removing the solvent under vacuum, the residue was loaded onto silica gel and purified via flash chromatography, using pentane/EtOAc mixtures as the eluent (starting from 100% pentane to 100% EtOAc), to isolate the product.

Based on the well established mechanism for the Pd-catalyzed carbonylative Suzuki coupling an oxidative addition step followed by CO insertion precedes the transmetallation event between the palladium intermediate and the boronic acid.[2] By controlling the presence of both CO and the transmetallating agent, it is possible to manage the selectivity of the process. Conversely, when dripping the aryl halide under low concentrations of CO, there are more opportunities for transmetallation between the oxidative addition palladium intermediate to occur before the CO insertion, leading to a loss of selectivity.

On-demand produced carbon monoxide used in the alkoxycarbonylation

In a 10 ml Schlenk flask, equipped with stirring bar 1.1 mg of $Pd(OAc)_2$ (0.005 mmol, 5 mol%) and 2.1 mg of 1,3-bis(diphenylphosphino)propane (dppp, 0.005 mmol, 5 mol%) were introduced. The system is capped with a septum and put under inert environment through 3 vacuum/N_2 cycles. Separately, in a 5 ml vial with a septum cap, 45.3 mg of [1,1'-biphenyl]-4-yl trifluoromethanesulfonate (0.15 mmol) was dissolved in 1.5 ml of anhydrous dioxane. The solution was sonicated and purged with N_2 to ensure complete solubilization of the substrate and deoxygenation of the mixture. Then, 0.2 ml of anhydrous methanol and 0.3 ml of anhydrous dioxane were added to the 10 ml Schlenk flask followed by 42 µl of triethylamine (TEA, 0.3 mmol, 3 eq.) under nitrogen flow. The system is then purged with the photoreactor output for 30 minutes with continuous stirring at room temperature. In the meanwhile, a 3 ml syringe (ø = 9.5 mm) was charged with the [1,1'-biphenyl]-4-yl trifluoromethanesulfonate solution. The syringe is mounted on the syringe pump and a flow rate of 0.0028 ml/min was set to drop in 1 ml (0.1 mmol, 1.0 eq.) of the substrate solution. Once all preparations are complete, the Schlenk reactor was put inside to the oil bath (previously set at 70°C) with the beginning of the addition of the limiting reagent solution via the syringe pump. At the end of the 6 hours of addition, the mixture is left to react for an additional 30 minutes after which the mixture is let to cool down to room temperature and then flushed with nitrogen to remove the CO present inside the reactor. The reaction mixture was extracted with EtOAc/water, and the organic layer was subsequently dried over Na_2SO_4. After removing the solvent under vacuum, the residue was loaded onto silica gel and purified via flash chromatography, using pentane/EtOAc mixtures as the eluent (from 100% pentane to 20% of EtOAc in pentane), to obtain the product.

On-demand produced carbon monoxide used in the aminocarbonylation

In a 10 ml Schlenk flask, equipped with stirring bar 9.2 mg of $Pd_2(dba)_3$ (0.01 mmol, 10 mol%) and 5.2 mg of triphenylphosphine (tpp, 0.02 mmol, 20 mol%) were introduced. The system is capped with a septum and put under inert environment through 3 vacuum/N_2 cycles. Separately, in a 5 ml vial with a

septum cap, 35.7 mg of (*E*)-1-iodooct-1-ene (0.15 mmol) was dissolved in 1.5 ml of anhydrous dioxane. The solution was sonicated and purged with N_2 to ensure complete solubilization of the substrate and deoxygenation of the mixture. Then, 0.5 ml of anhydrous dioxane was added to the 10 ml Schlenk flask followed by 42 µL of triethylamine (TEA, 0.3 mmol, 3 eq.) and 33 µL of 1-hexylamine (0.25 mmol, 2.5 eq.) under nitrogen flow. The system is then purged with the photoreactor output for 30 minutes with continuous stirring at room temperature. In the meanwhile, a 3 ml syringe (ø = 9.5 mm) was charged with the (*E*)-1-iodooct-1-ene solution. The syringe is mounted on the syringe pump and a flow rate of 0.0035 ml/min was set to drop only 1 ml (0.1 mmol, 1.0 eq.) of the substrate solution. Once all preparations are complete, the Schlenk reactor was put inside the oil bath (previously set at 80°C) with the beginning of the addition of the limiting reagent solution via the syringe pump. At the end of the 5 hours of addition, the mixture is left to react for an additional 30 minutes after which the mixture is let to cool down to room temperature and then flushed with nitrogen to remove the CO present inside the reactor. The reaction mixture was extracted with EtOAc/water, and the organic layer was subsequently dried over Na_2SO_4. After removing the solvent under vacuum, the residue was loaded onto silica gel and purified via flash chromatography, using pentane/EtOAc mixtures as the eluent (from 100% pentane to 50% of EtOAc in pentane), to obtain the product.

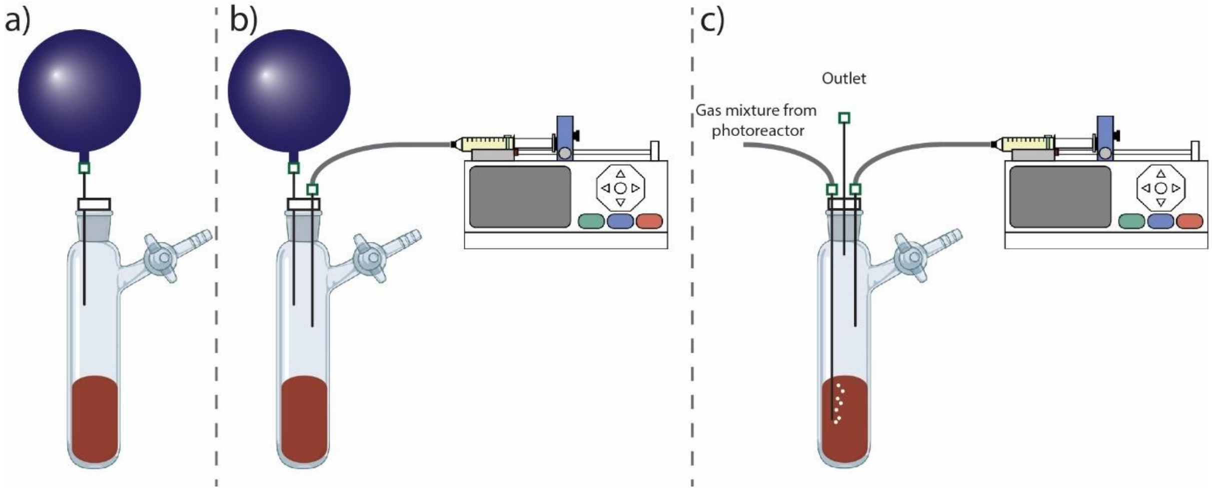

Figure S2 Overview of the batch (a), fed-batch (b), and coupled (c) system to carry out different carbonylation reactions.

S2 Gas chromatography

S2.1 Analysis

For analysis a Shimadzu Nexis GC-2030 gas chromatograph, equipped with both a flame ionization detector (FID) and a thermal conductivity detector (TCD), was used. A two column system (SH-Q-BOND and SH-Msieve 5A) with a bypass was employed to analyze all relevant gaseous compounds. The analysis was operated online, meaning that the outlet of the reactor system is continuously flowing and exiting the system through the vent. When starting the analysis, 50 µl (sample loop volume) of the gaseous sample is taken and sent to the column. The method uses a split injection (split ratio 5.0) at an injector temperature of 80 °C. A helium carrier gas is used, and all compounds pass the first column (Q-BOND). Next, the light gases are parked in the second column (Msieve 5A) and after the methane exits the first column the valve switches and the carbon dioxide will bypass the second column to go to the TCD (200 °C, helium reference) and FID detector (jetanizer, 280 °C), as shown in Figure S3. After the carbon dioxide has passed the FID, the parked gases are flowed to the detectors. The retention times of methane and carbon dioxide need to be separately measured to determine the time of the valve switches and develop the method. During the analysis the oven is kept at 40 °C.

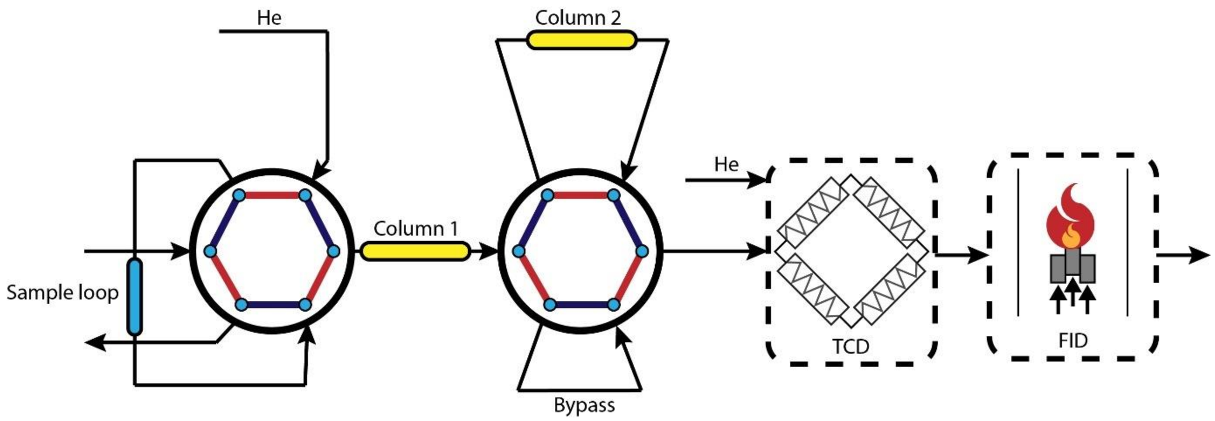

Figure S3 Overview of the gas chromatography setup, including the sample loop, 2 valves, 2 columns (1: SH-Q-BOND and 2: SH-Msieve 5A) and the detectors.

Calibrations for carbon monoxide, methane, carbon dioxide and hydrogen have been made for the corresponding detector (FID or TCD, see Figure S4). All compounds were used in the calculation of the conversion of carbon dioxide, providing a standard based on the expected reactions. Corresponding calibrations were made with mass flow controllers, one providing the compound for calibration and another one providing either nitrogen, carbon dioxide or hydrogen. Volume fractions in the sample loop were determined based on the gas properties, supplied in ml_n (volume at normal conditions; 0 °C and 1 atm).

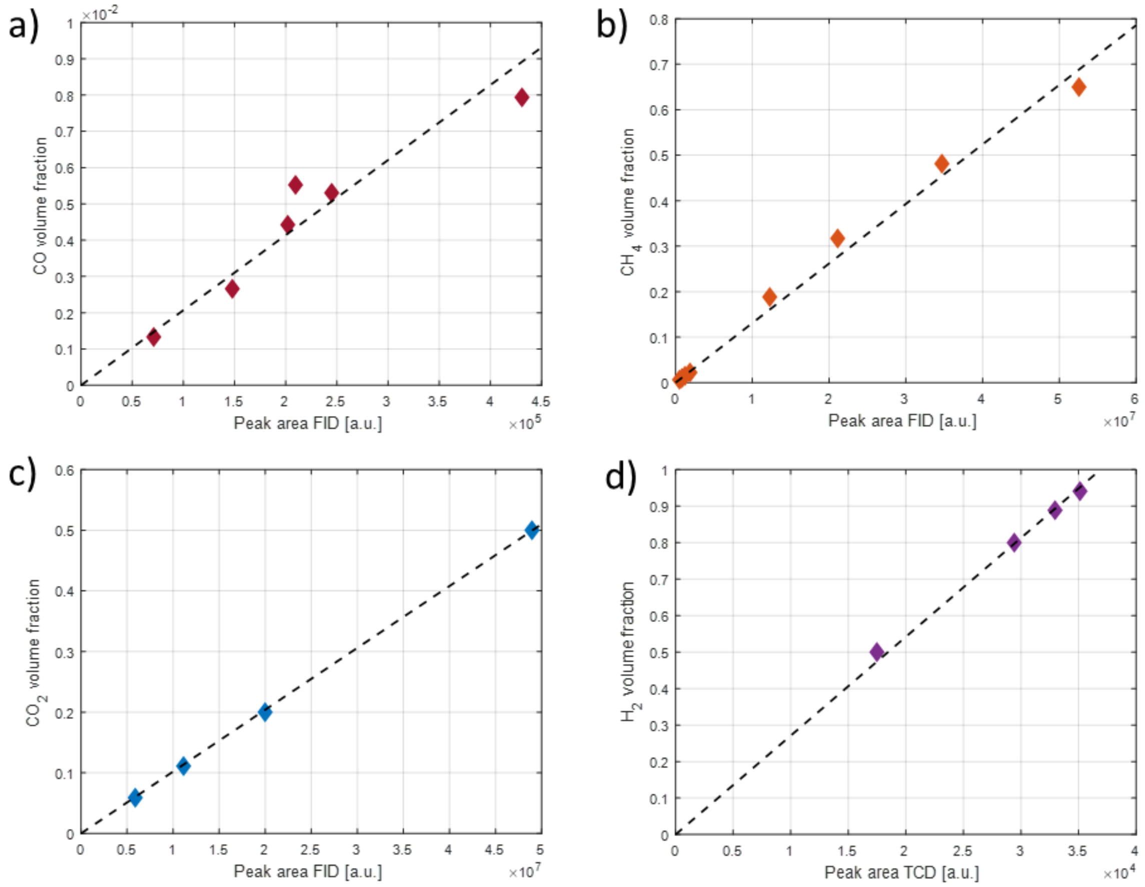

Figure S4 a) FID calibration for carbon monoxide at different sample loop compositions ($y=2.07\cdot10^{-8}x$, $R^2=0.990$). b) FID calibration for methane at different sample loop compositions ($y=1.31\cdot10^{-8}x$, $R^2=0.994$). c) FID calibration for carbon dioxide at different sample loop compositions ($y=1.02\cdot10^{-8}x$, $R^2=0.999$). d) TCD calibration for hydrogen at different sample loop compositions ($y=2.71\cdot10^{-5}x$, $R^2=0.999$).

S2.2 Correction for non-equimolar reactions

Gaseous carbon dioxide and hydrogen are assumed to only form methane, carbon monoxide and water, according to the Sabatier and reverse water gas-shift reaction (Reaction 1 and 2). The gas phase is sampled at the outlet of the reactor. The water formed is assumed to not enter the GC, which is supported by the results obtained during the experiments (sum of volume fractions was found to be approximately equal to 1, excluding the water). The amount of condensed water formed at the set flow rates, was assumed to be negligible in terms of interfering with the reaction or analysis.

1 $$CO_2 + 4H_2 \rightarrow CH_4 + 2H_2O$$

2 $$CO_2 + H_2 \rightleftharpoons CO + H_2O$$

If the water is discarded from the equations, it can be noted that these reactions are not equimolar, meaning less moles of gas are formed than consumed, resulting in smaller volumes at constant pressure (pressure gradients are automatically eliminated). The volume of the sample loop is fixed at 50 µl, leading to over-sampling, since more moles are drawn in to fill the sample loop volume, causing falsified results if not accounted for. An adjustment in the data analysis has to be made to correct for the shrinkage of volume. For this correction it is assumed that Reaction 1 and 2 occur exclusively and water does not occupy any gaseous volume in the sample loop.

The number of moles of every compound can be expressed as functions of the conversions towards the respective products. For convenience, these are labelled as; 1=CO_2, 2=H_2, 3=CH_4, 4=CO. Here x denotes the conversion of 1→3 and y of 1→4. In Table S1 the initial, denoted 0 with ($x=y=0$), and final, denoted τ with ($x\geq0$ & $y\geq0$) states and total amount of moles are shown.

Table S1 An overview of the initial and final number of moles of carbon dioxide, hydrogen, methane and carbon monoxide.

	$i=1$	$i=2$	$i=3$	$i=4$
$n_{i,0}$	$n_{1,0}$	$n_{2,0}$	0	0
$n_{i,\tau}$	$n_{1,0}(1-x-y)$	$n_{2,0}-n_{1,0}(4x+y)$	$n_{1,0}x$	$n_{1,0}y$

The initial and final total amount of moles (n_T) can be summed for all compounds i (Equation 1 and 2). Since this total number of moles from initial to final decreases, the additional sampling to draw the same number of moles/volume can be expressed as the factor f (Equation 3).

$$n_{T,0} = \sum_i n_{i,0} = n_{1,0} + n_{2,0} \quad (1)$$

$$n_{T,\tau} = \sum_i n_{i,\tau} = n_{2,0} + n_{1,0}(1-4x-y) \quad (2)$$

$$f = \frac{n_{T,0}}{n_{T,\tau}} = \frac{n_{1,0}+n_{2,0}}{n_{2,0}+n_{1,0}(1-4x-y)} \quad (3)$$

Here, $n_{T,0}$ is defined as the amount of moles in the sampled volume, consisting of the original composition of compounds 1 and 2 when no reaction has occurred. This original composition can be related to the reactor feed as $\nu = \frac{n_{2,0}}{n_{1,0}}$, the H_2/CO_2 feed ratio. The total amount of moles for each component n_i within the drawn sample can be expressed as such that $n_i = n_{i,\tau} \cdot f$, giving Equation 4 to 7. By definition, the summation of all n_i is equal to $n_{T,0}$ because of correction with f.

$$n_1 = n_{1,\tau} f = n_{1,0}(1-x-y)\cdot f \quad (4)$$

$$n_2 = n_{2,\tau} f = \left(n_{2,0} - n_{1,0}(4x+y)\right)\cdot f \quad (5)$$

$$n_3 = n_{3,\tau} f = n_{1,0}x \cdot f \quad (6)$$

$$n_4 = n_{4,\tau} f = n_{1,0}y \cdot f \quad (7)$$

The actual data obtained from an experiment is the area of the peak of component *i*, measured by GC (FID or TCD). The aforementioned given calibrations, lead to the volume fraction α_i within the sample loop. This can then be coupled with the molar amounts (given by the ideal gas law), shown in Equation 8, eventually leading to Equation 9 to 12.

$$\alpha_i = \frac{V_i}{V_T} = \frac{n_i}{n_{T,0}} = \frac{n_{i,\tau} f}{n_{T,0}} = \frac{n_{i,\tau} n_{T,0}}{n_{T,0} n_{T,\tau}} = \frac{n_{i,\tau}}{n_{T,\tau}} \quad (8)$$

$$\alpha_1 = \frac{n_{1,\tau}}{n_{T,\tau}} = \frac{n_{1,0}(1-x-y)}{n_{2,0}+n_{1,0}(1-4x-y)} \quad (9)$$

$$\alpha_2 = \frac{n_{2,\tau}}{n_{T,\tau}} = \frac{n_{2,0}-n_{1,0}(4x+y)}{n_{2,0}+n_{1,0}(1-4x-y)} \quad (10)$$

$$\alpha_3 = \frac{n_{3,\tau}}{n_{T,\tau}} = \frac{n_{1,0}x}{n_{2,0}+n_{1,0}(1-4x-y)} \quad (11)$$

$$\alpha_4 = \frac{n_{4,\tau}}{n_{T,\tau}} = \frac{n_{1,0}y}{n_{2,0}+n_{1,0}(1-4x-y)} \quad (12)$$

The products can be related to the reagents, so 3 and 4 both to 1 and 2 respectively (Equation 13 and 14). With the measured data, x and y can be obtained by substitution and re-writing of the equations. R_3 and R_4 are in this form given by Equation 16 and 17, leading to the expressions of x and y in Equation 18 and 19.

$$R_3 = \frac{\alpha_3}{\alpha_1+\alpha_2} \quad (13)$$

$$R_4 = \frac{\alpha_4}{\alpha_1 + \alpha_2} \tag{14}$$

$$R_i = \frac{\alpha_i}{\alpha_1 + \alpha_2} = \frac{\frac{n_{i,\tau}}{n_{T,\tau}}}{\frac{n_{1,\tau}}{n_{T,\tau}} + \frac{n_{2,\tau}}{n_{T,\tau}}} = \frac{n_{i,\tau}}{n_{1,\tau} + n_{2,\tau}} = \frac{n_{i,\tau}}{n_{1,0}(1 - x - y) + n_{2,0} - n_{1,0}(4x + y)} \tag{15}$$

$$R_3 = \frac{x}{(1 - 5x - 2y) + \nu} \tag{16}$$

$$R_4 = \frac{y}{(1 - 5x - 2y) + \nu} \tag{17}$$

$$y = \frac{R_4(1 + \nu)}{1 + 5R_3 + 2R_4} \tag{18}$$

$$x = \frac{R_3(1 + \nu)}{1 + 5R_3 + 2R_4} \tag{19}$$

S3 Catalyst characterization

The deposition of all the examined catalyst (1 wt%) coated glass beads was analyzed using a field emission scanning electron microscope (FESEM, model Auriga Compact from ZEISS company, equipped with X-Ray detector (EDS) and electron backscatter diffraction (EBSD)). Samples were prepared according to S1.1 CO_2 hydrogenation catalyst preparation and loading, and a fraction of the beads was deposited on carbon tape. Subsequently, a thin layer of carbon was sputtered (sputter EM MED020 from LEICA) to provide conductivity to the samples. A reference is provided by non-coated glass beads, shown in Figure S5, displaying the particle size distribution of the beads. Figure S6, Figure S10, Figure S11 and Figure S16 indicate that the beads are comparably coated with the catalyst powder.

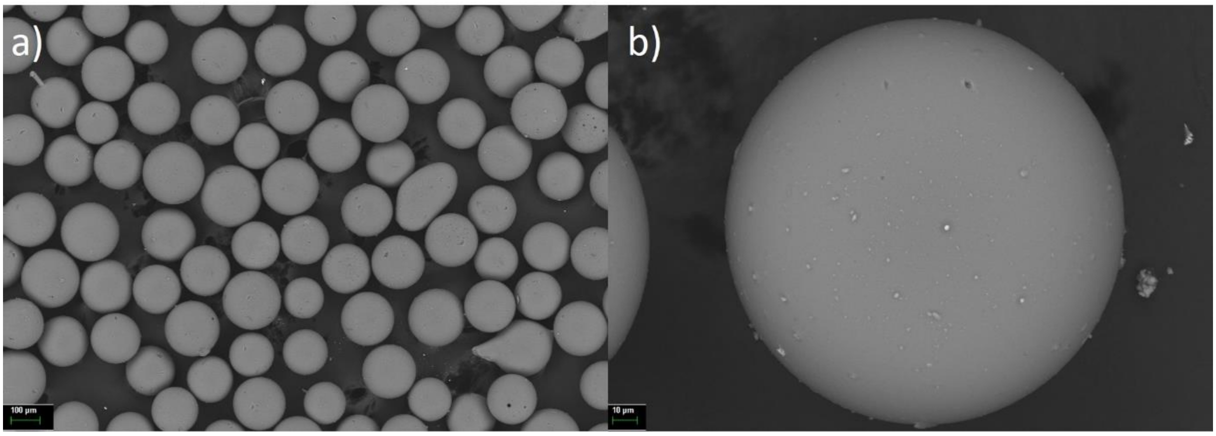

Figure S5 Non-coated glass beads scanning electron microscopy image at different scales (a and b).

Cobalt(II,III) oxide

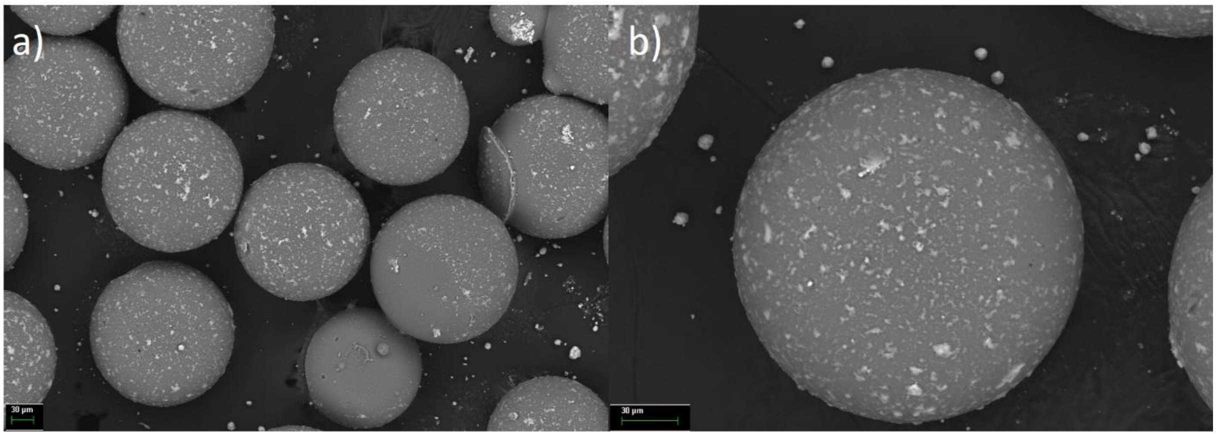

Figure S6 Cobalt(II,III) oxide (1 wt%) coated glass beads scanning electron microscopy image at different scales (a and b).

Ruthenium nanoparticles (5.76 wt%, 1.79 ± 0.51 nm) on titanium dioxide (Evonik Aeroxide P90, < 45 µm)

The ruthenium content in the catalyst was analyzed via inductively coupled plasma atomic emission spectroscopy (ICP-AES) in axial detection mode. Hereto, the sample was digested using a 10 ml mixture of mineral acids ($HCl:HNO_3:HF$ in a 3:1:1 volumetric ratio) in a Milestone microwave setup, whereupon dilution in a 50 ml polypropylene flask was carried out. The digestion procedure is executed *in duplo* and average values are reported. An external calibration curve is used for quantification starting from a 1000 ppm Merck Ru standard in 7% HCl. 5 ppm QC measurements are regularly checked to assure the instrument's constant performance. High-resolution transmission electron microscopy (HRTEM, Figure S7a) studies were performed using a JEOL ARM 200F Transmission Electron Microscope. Imaging was performed in High Angle Annular Dark Field (HAADF) Scanning TEM

mode. The HAADF detector uses the electrons scattered over large angles for imaging. The HAADF detector is therefore mass sensitive, which means that higher brightness in the image corresponds to the presence of (a larger concentration of) heavier atoms. This allows for a fast and accurate inventory of the ruthenium particle size on the TiO_2 support. Samples were prepared by preparing a suspension of the material in ethanol and by depositing a drop of this suspension onto a carbon-coated copper TEM grid and drying at room temperature. In order to determine the average size of the ruthenium nanoparticles and the particle size distribution (shown in Figure S7b), we measured the dimensions of 250 nanoparticles from the corresponding HRTEM micrograph, using the image analysis software ImageJ.

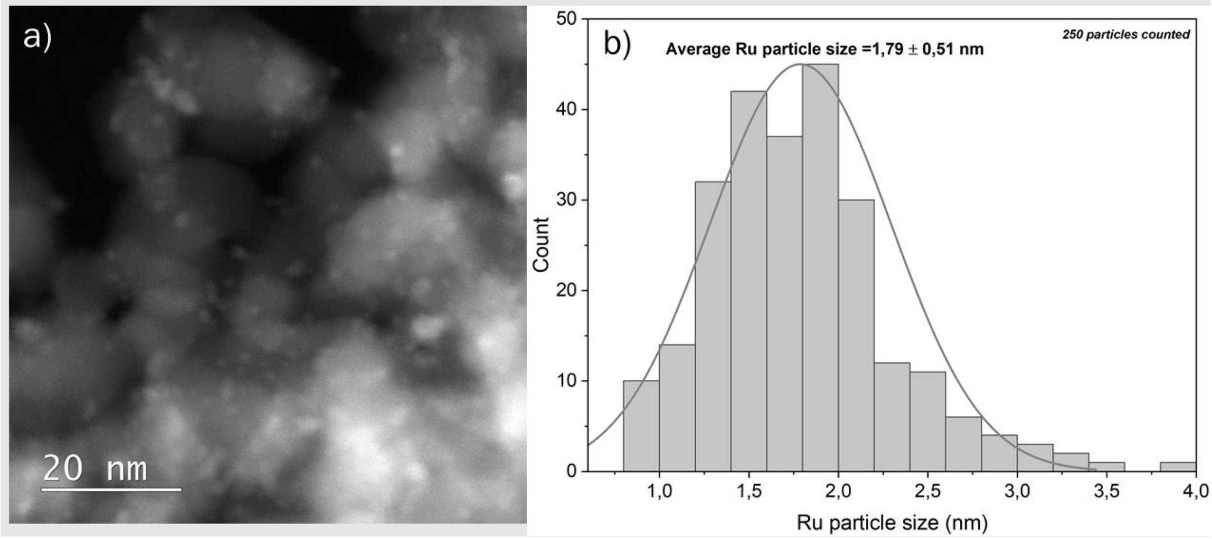

Figure S7 Ruthenium nanoparticles (on TiO_2 support) high-resolution transmission electron microscopy micrographs (a) and associated size distribution histogram (b).

X-ray diffraction (XRD) data sets in Figure S8 were collected using a powder diffractometer (Panalytical) equipped with a Prefix Bragg BrentanoHD mirror and a copper radiation source with a fixed slit of ¼ inch. A Pixcel 1d detector and an anti-scatter slit of 1 inch were used. The incident beam path is 4.41˚, radius 240 mm. The used wavelength is K-Alpha1 (1.5405980 Å). Powder samples were prepared in a ceramic sample holder.

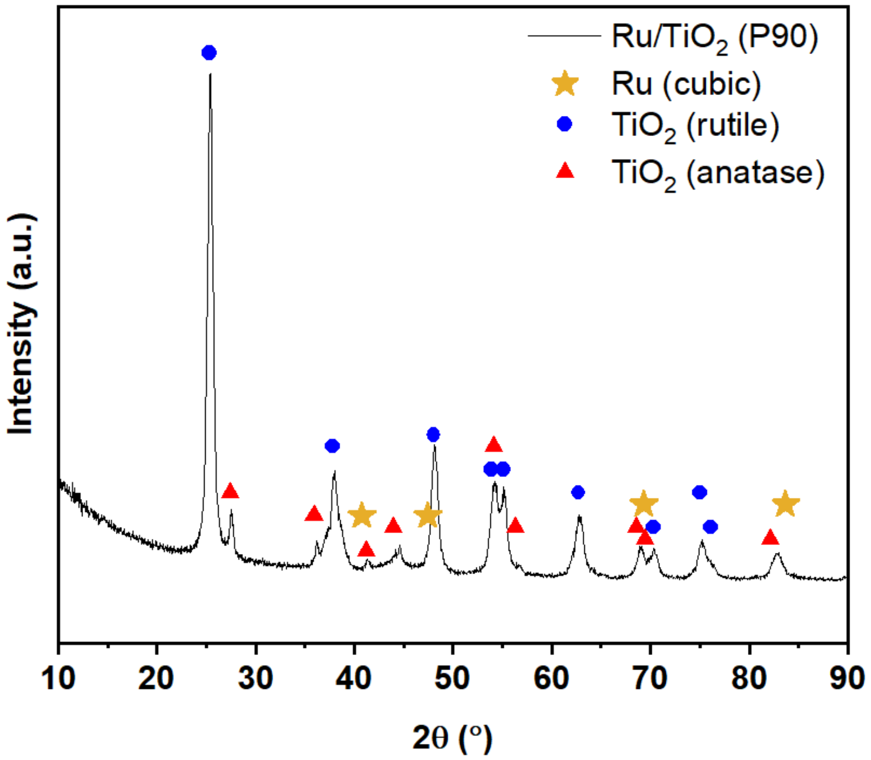

Figure S8 XRD pattern of the Ru/TiO2 (P90) nanocatalyst showing TiO2 anatase, TiO2 rutile and Ru cubic peak positions.

The UV-Visible diffuse reflectance spectrum (Figure S9) was obtained using a Shimadzu UV-3600 spectrophotometer. The powder samples were pressed on a support, and their reflectance was measured in the range between 300 nm and 1200 nm through an integrating sphere. The baseline for the measurements was done with $BaSO_4$.

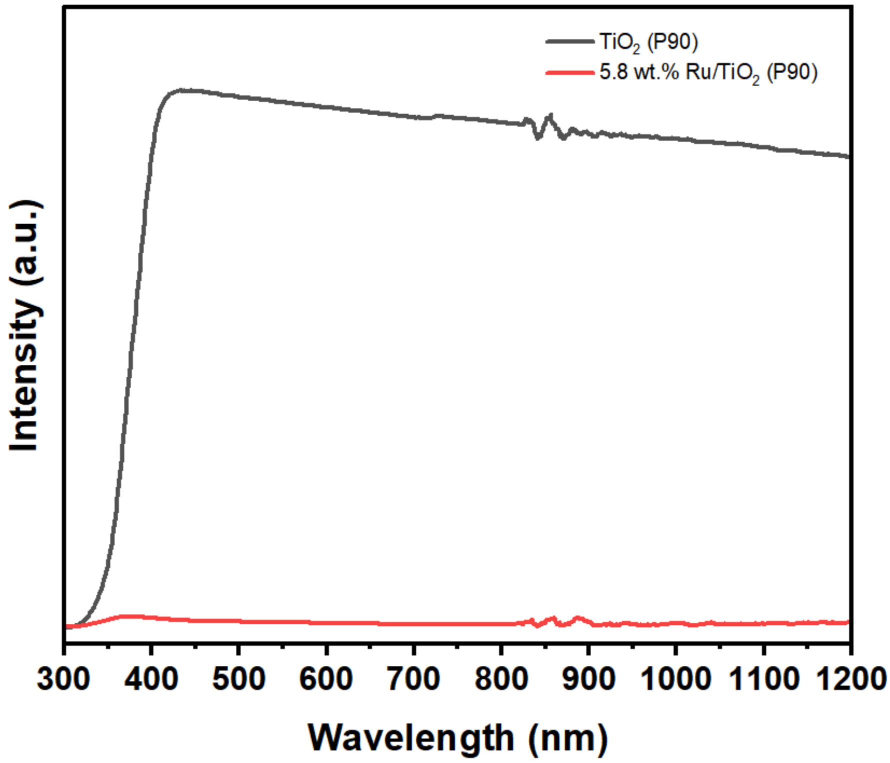

Figure S9 Diffuse reflectance UV–Vis NIR spectra for the TiO2(P90) (black curve) and Ru/TiO2 (red curve).

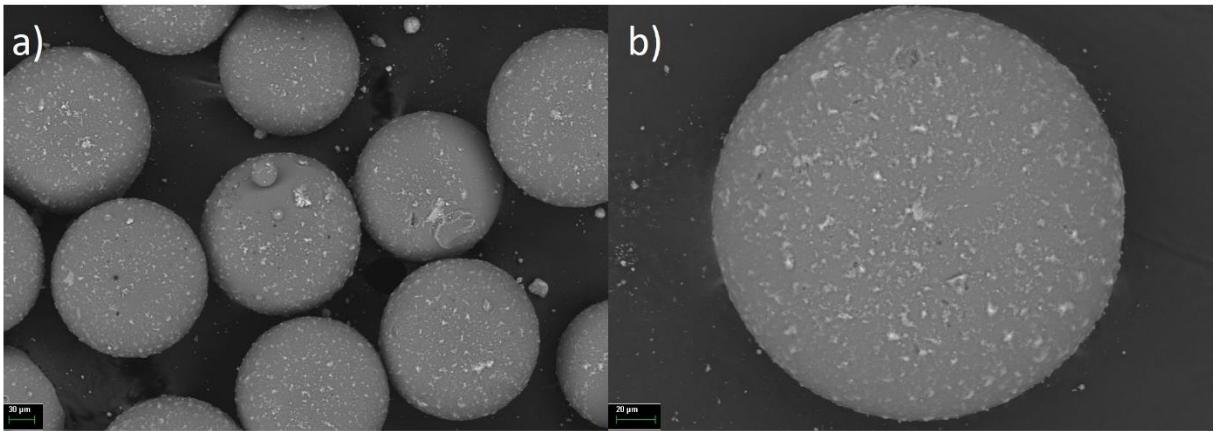

Figure S10 Ru/TiO$_2$ (1 wt%) coated glass beads scanning electron microscopy image at different scales (a and b).

Ruthenium nanoparticles (2.5 wt%, 1.4 ± 0.4 nm) on strontium titanate (80 nm)

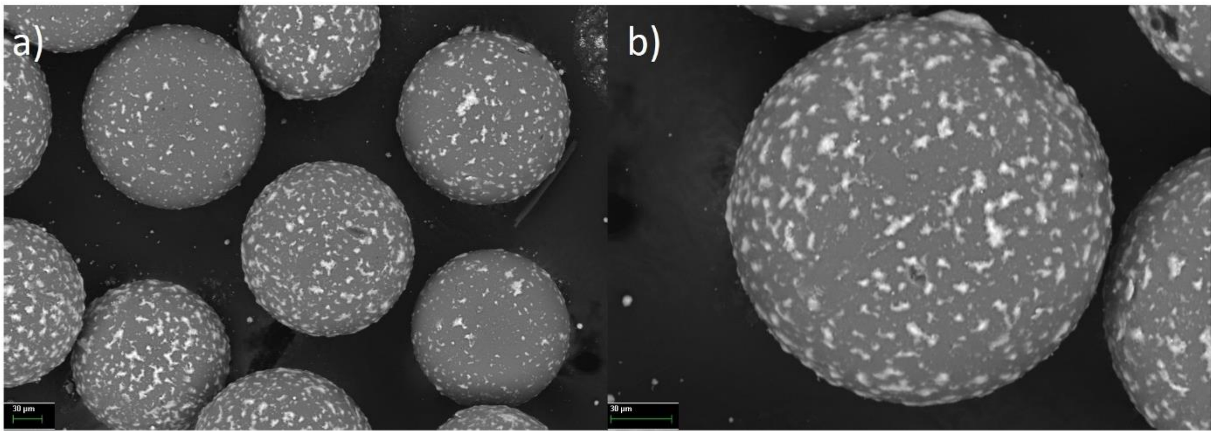

Figure S11 Ru/STO (1 wt%) coated glass beads scanning electron microscopy image at different scales (a and b).

Gold nanoparticles (2.5 wt%, 2.44 ± 0.61 nm) on titanium dioxide (Evonik Aeroxide P90, < 45 µm)

The gold content in the catalyst was analyzed via ICP-AES in axial detection mode. Hereto, the sample was digested using a 10 ml mixture of mineral acids (HCl: HNO_3:HF in a 3:1:1 ratio) in a Milestone microwave setup, whereupon dilution in a 50 ml polypropylene flask was carried out. The digestion procedure is executed *in duplo* and average values are reported. An external calibration curve is used for quantification starting from a 1000 ppm Perkin Elmer Au standard in 10% HCl. 5 ppm QC measurements are regularly checked to assure the instrument's constant performance. HRTEM (Figure S12) studies were performed using a JEOL ARM 200F Transmission Electron Microscope. Imaging was performed in HAADF Scanning TEM mode. An inventory of the gold particle size on the TiO_2 support is shown in Figure S13. Samples were prepared by preparing a suspension of the material in ethanol and by depositing a drop of this suspension onto a carbon-coated copper TEM grid and drying at room temperature.

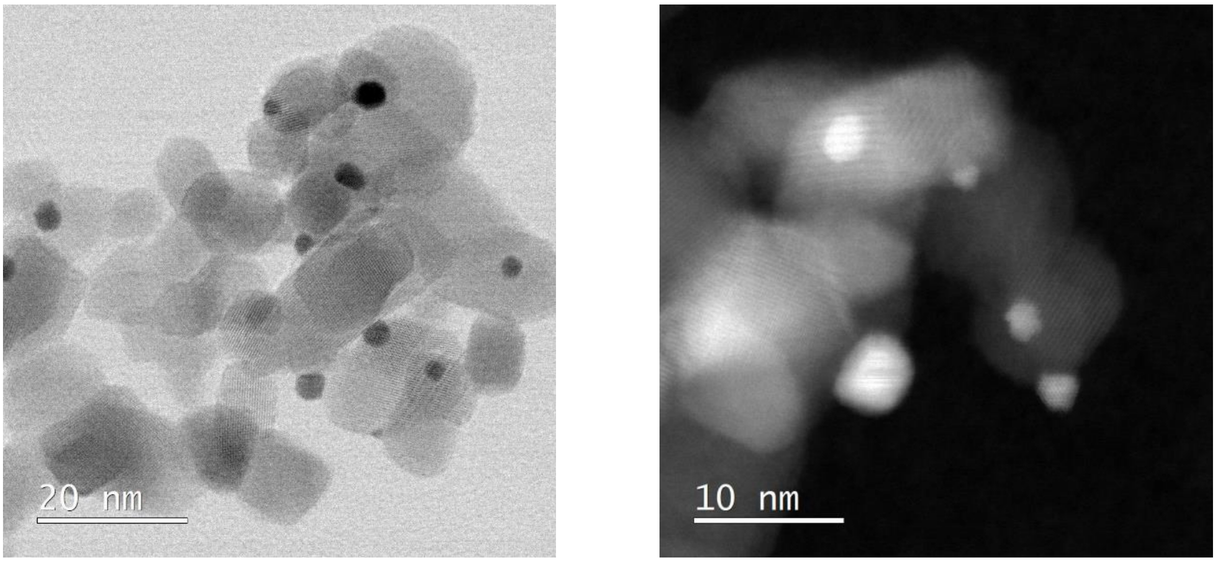

Figure S12 Representative high-resolution transmission electron microscopy images of the gold supported on TiO_2.

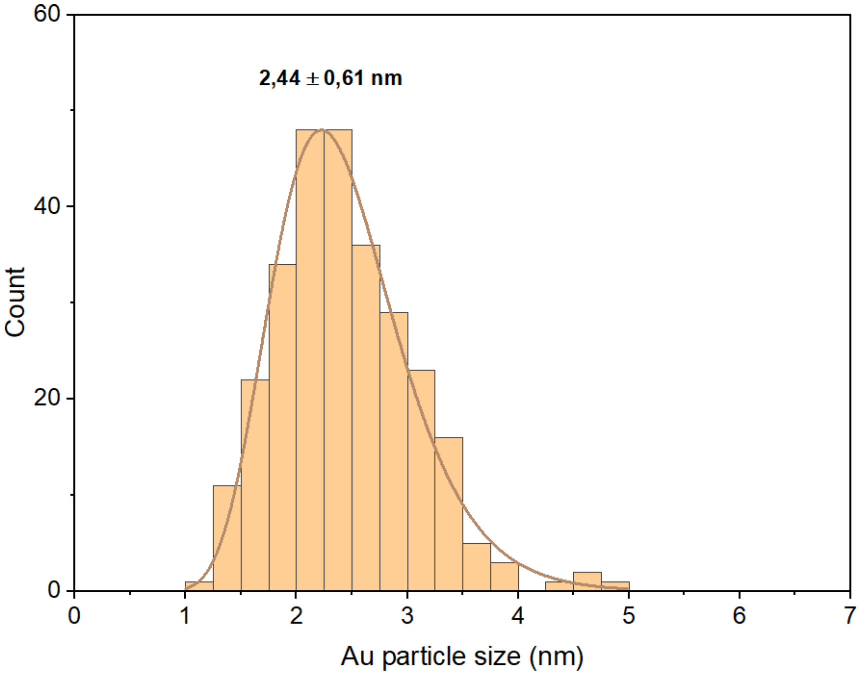

Figure S13 Gold nanoparticle size distribution histogram.

XRD data sets in Figure S14 were collected using a powder diffractometer (Panalytical) equipped with a Prefix Bragg BrentanoHD mirror and a copper radiation source with a fixed slit of ¼ inch. A Pixcel 1d detector and an anti-scatter slit of 1 inch were used. The incident beam path is 4.41˚, radius 240 mm. The used wavelength is K-Alpha1 (1.5405980 Å). Powder samples were prepared in a ceramic sample holder.

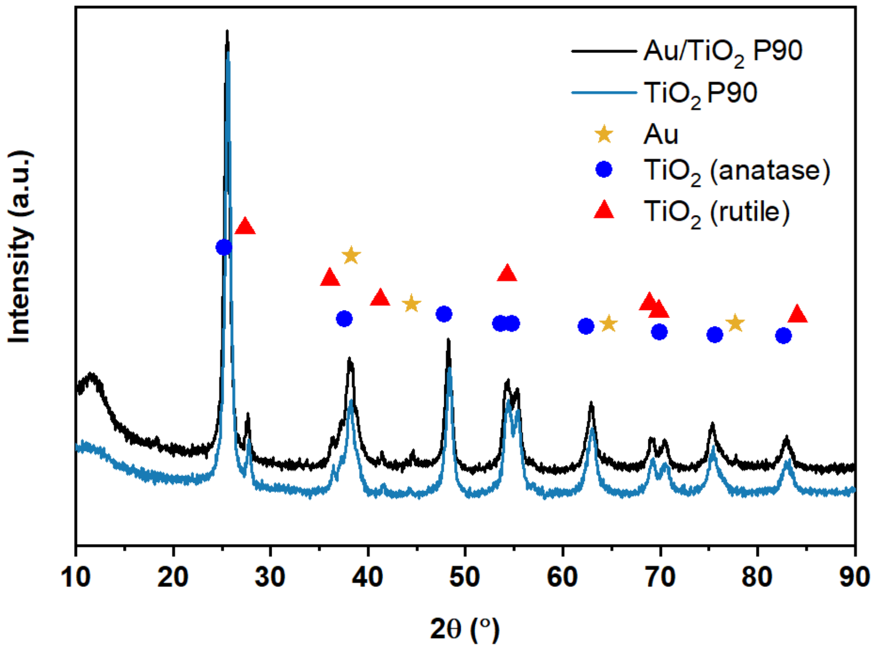

Figure S14 XRD pattern of the Au/TiO_2 nanocatalyst.

The UV-Visible diffuse reflectance spectrum (Figure S15) was obtained using a Shimadzu UV-3600 spectrophotometer. The powder samples were pressed on a support, and their reflectance was measured in the range between 300 nm and 1200 nm through an integrating sphere. The baseline for the measurements was done with $BaSO_4$.

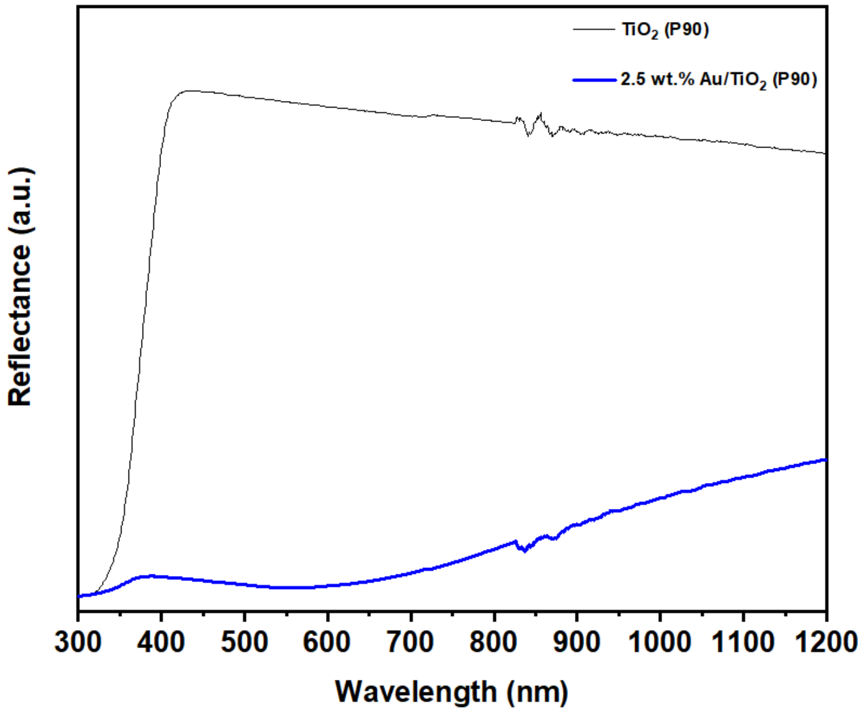

Figure S15 Diffuse reflectance UV–Vis NIR spectra for TiO_2 (P90) (black curve) and Au/TiO_2 (blue curve).

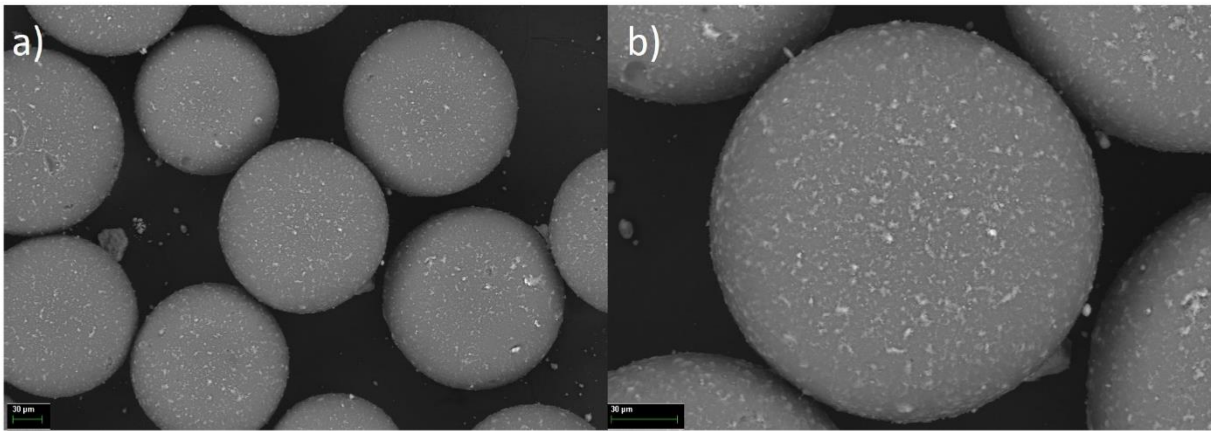

Figure S16 Au/TiO_2 (1 wt%) coated glass beads scanning electron microscopy image at different scales (a and b).

S4 Reactor system

S4.1 Design

The oil tank was designed to be operated in a vertical or tilted position. The quartz window (3.0 mm) was put into place with seven bolts, where Teflon sealings prevented the leaking of the oil. A custom made reactor connector allowed for the use of the Little Things Factory photoreactor (Borofloat®33, ID 1.0 mm, 1.1 ml), to which the gas inlet and outlet could be connected using Teflon ferrules. The reactor connector was fixated in place with two bolts. The thermocouples and connections to control the ceramic heating rods were supplied from the side of the casing and were connected to the PID controller. All elements were placed on a backplate for safety and stability. Pressure build up in the oil tank was prevented by the use of an oil overflow system, where excess oil ends up in a reservoir (at atmospheric pressure). Additional oil could be supplied by an inlet at the bottom of the oil tank, which was connected to a switch valve. Further details on the separate parts, dimensions and construction are shown in Figure S17 and Figure S18.

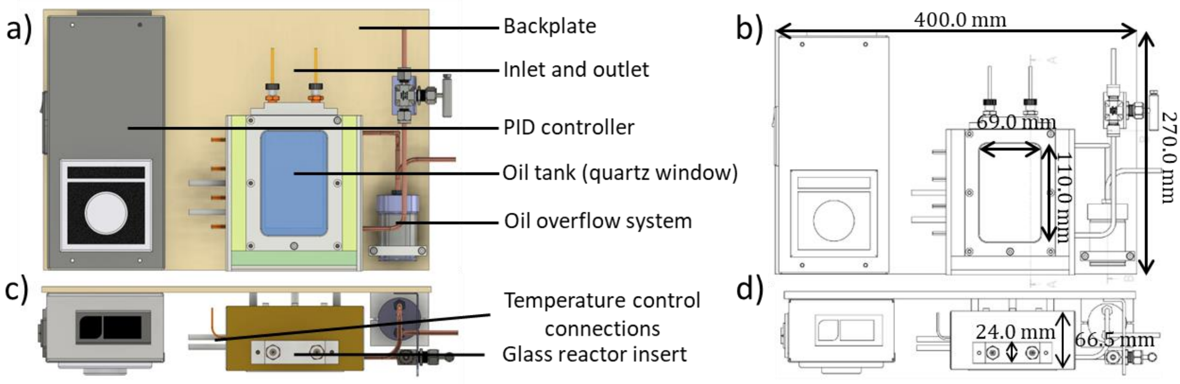

Figure S17 a) Front view of the 3D model of the reactor system. b) Front view of the technical drawing of the reactor system. c) Top view of the 3D model of the reactor system d) Top view of the technical drawing of the reactor system.

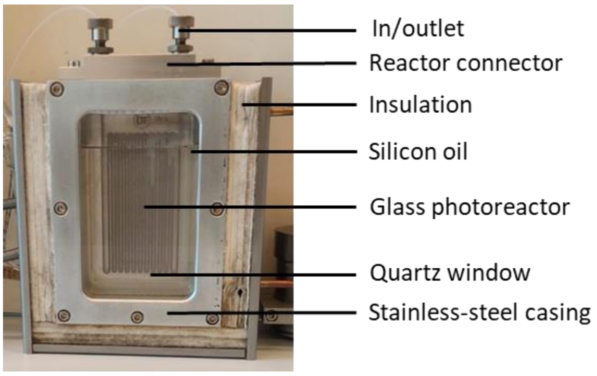

Figure S18 Image of the oil tank with the immersed photoreactor filled with catalyst-coated beads.

S4.2 Silicon oil

The absorbance of the silicon oil (methoxy-dimethyl-[methyl(diphenyl)silyl]oxysilane, viscosity 400 cst, max. 250-315°C, CAS 68083-14-7) was measured to check for competitive absorption within the solar spectrum. Negligible absorbance over the investigated wavelength range was found for the transparent oil, as can be seen in Figure S19.

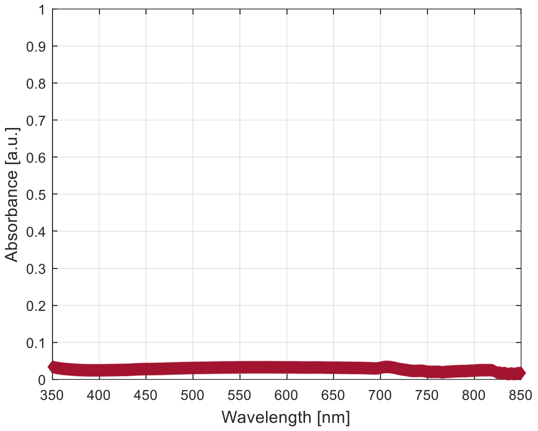

Figure S19 The absorbance of the used silicon oil over the investigated wavelength range.

S4.3 Light source

White LEDs (CXB3590, cool white, 86 W electrical input power per chip, 4 chips) were used to test the catalyst upon visible light irradiation. The spectrum of the light source is shown in Figure S20a, determined using an optical fiber and a spectrophotometer (Avantes, AvaSpec-2048-USB2-UA). The system was placed at 2 cm from the light source, giving the intensity map of Figure S20b (at 100% of power input, measured with an Adafruit TSL2591 sensor, calibrated with an Extech SP505 solar power meter). The average intensity at the quartz window of the reactor system is approximately equal to 2400 W/m^2.

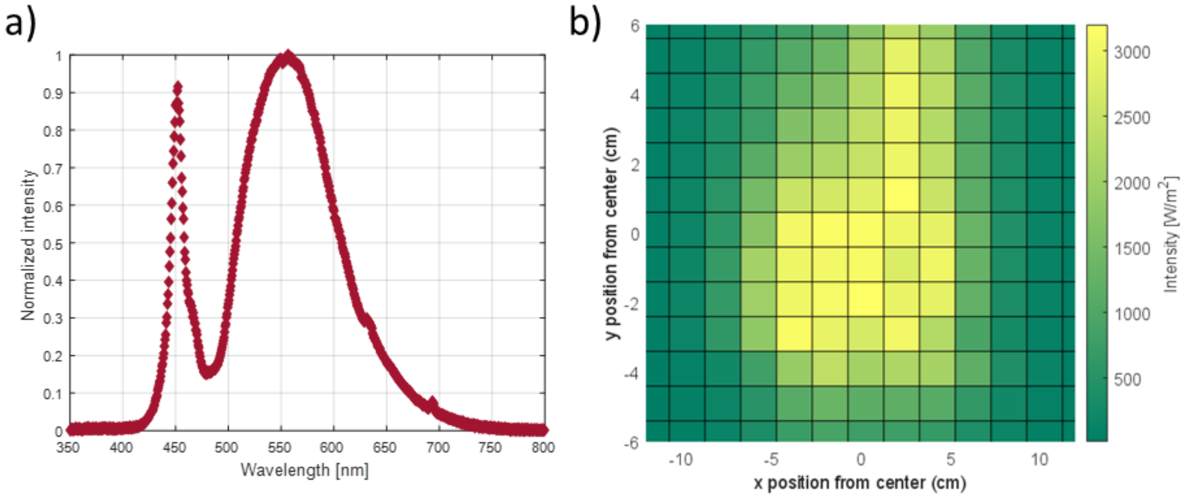

Figure S20 a) Normalized intensity of the employed white LEDs against the wavelength. b) Light intensity at different positions at 2 cm from the light source.

S4.4 Temperature control

Figure S21a shows the temperature response for a heating and cooling down cycle. The set temperature of 200 °C is reached after approximately 10 minutes. After 20 minutes the heating is turned off and the heat retaining properties of the designed system are illustrated by the gradual decrease in temperature. The control of the oil temperature was found to be independent of the light intensity, meaning that stable temperatures were reached in both cases (Figure S21b).

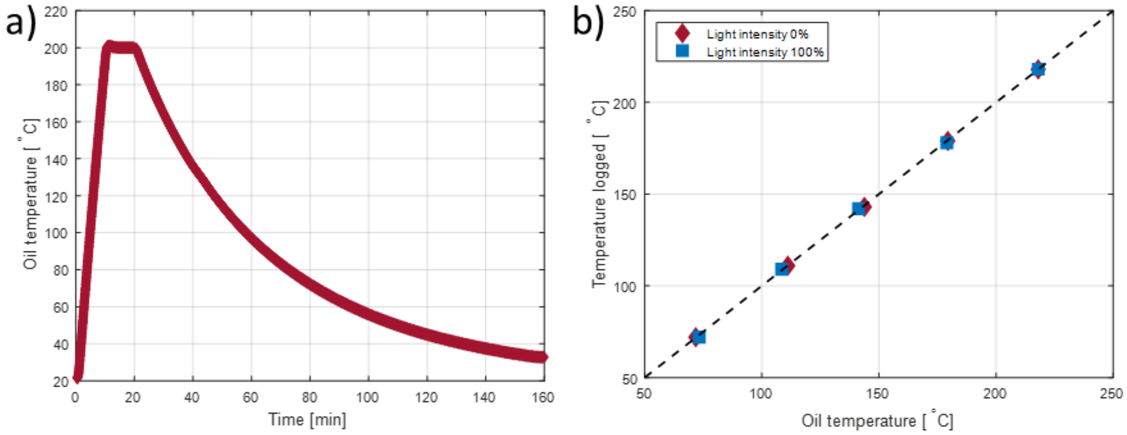

Figure S21 a) The time dependent oil temperature over a heating and cooling down cycle. b) The oil temperature (measured with an external thermocouple) against the temperature logged by the controller, with the light at 100 and 0% of the power.

The development of the gas temperature in the reactor can be estimated by setting up an energy balance over a steady-state plug flow system, as shown in Equation 20 (Where F is the molar flow in mol/s, C_p the molar heat capacity in J/mol/K, T the temperature in K, α the heat transfer coefficient in $W/m^2/K$ and D the inner diameter in m). Here heat generation within the system is assumed to be negligible in comparison to the heat transfer of the reaction channel to/from its surroundings. Moreover, the thermal properties of the gases are assumed to remain constant over the temperature range (values used for 25 °C, 25-250 °C). In the case of fully developed laminar incompressible flow through a tube, the heat transfer coefficient can be estimated from the developed Nusselt number for a uniform velocity profile, which is equal to 5.78.[3] Equation 20 can be solved to yield the temperature at position z within the tube (Equation 21). It can be noted from Figure S22 that both individual pure gases would reach the set temperature of the environment (250 °C) quickly (within 0.5 mm), where a stream of pure carbon dioxide would take relatively longer to reach the set temperature than a steam of pure hydrogen, for both assuming a flow rate of 1 ml_n/min.

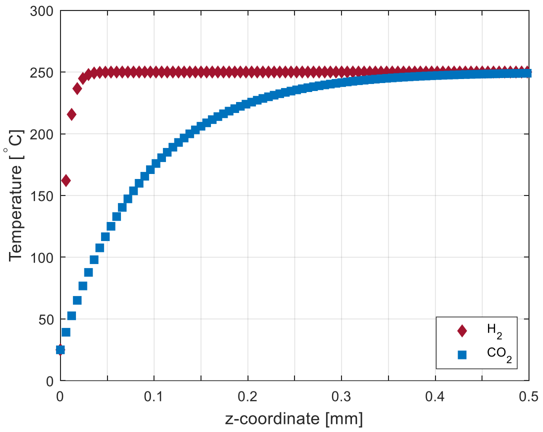

Figure S22 The evolution of the temperature of pure hydrogen and carbon dioxide over the z-coordinate for a laminar incompressible flow through a tube, assuming a uniform velocity profile (flow rate of 1 ml_n/min).

$$F C_p \frac{dT}{dz} = -\alpha \pi D (T - T_1) \qquad 20$$

$$\ln\left(\frac{T - T_1}{T_0 - T_1}\right) = \exp\left(-\frac{\alpha \pi D z}{F C_p}\right) \qquad 21$$

$$Nu = \frac{\alpha D}{\lambda} \qquad 22$$

$$Nu_{\infty} = 5.78 \qquad (23)$$

S4.5 Pressure drop

The pressure over the full reactor setup was measured with a pressure sensor (Huba Control type 548) at a catalyst coated (Au/TiO_2) glass bead loading of 1.9 grams, while flowing CO_2 at a reactor temperature of 200 °C (Figure S23). Using the Ergun equation, a fit for the effective particle diameter was made, which was equal to 399 µm. The size of the glass beads used is 150-212 µm (with a particle size distribution), supporting the range and order of magnitude of the diameter. The pressure drop of the tubing to and from the setup and sample loop were measured to be negligible (0.0 bar), as was expected from estimations made with the Hagen-Poiseuille equation. The system was found to be leak-proof as pressure could be maintained for a substantial amount of time without replenishing the gases.

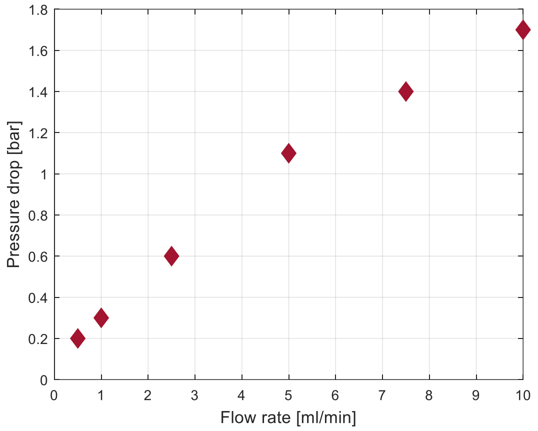

Figure S23 The measured pressure drop of the entire system (tubing and packed bed reactor) against different overall flow rates.

S5 Automation

S5.1 System Overview

The system is comprised of five devices (Table S2) each connected to a dedicated computer via USB (Figure S24). Each device can be independently controlled in order to affect one or more reaction parameters. Some of these parameters could also be monitored in real-time by the same device and recorded by the control unit.

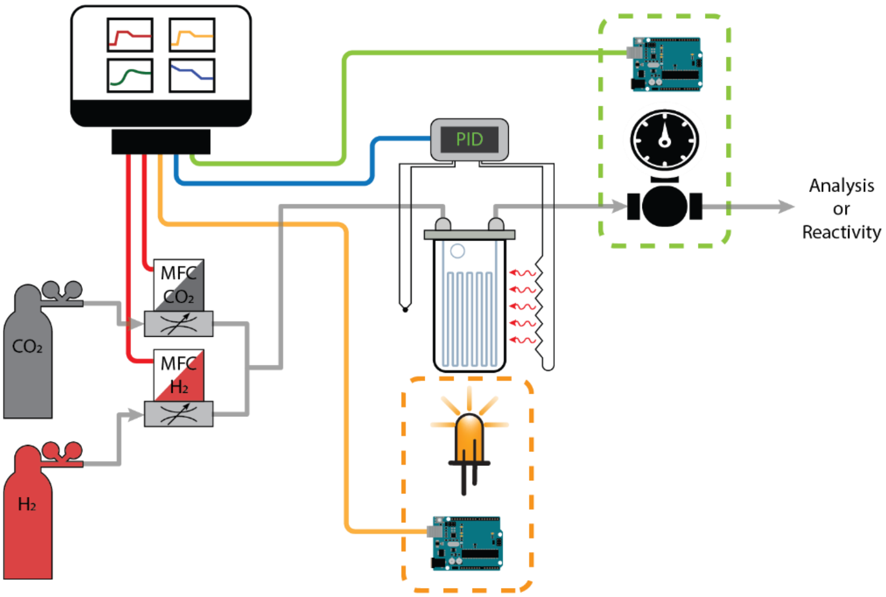

Figure S24 Overview of the devices connected and controlled by the automation system.

Table S2 Automation devices and connection protocols.

Device	Reaction Parameter	Manufacturer	Model	Communication Protocol
**Mass Flow Controller**	CO_2 flow-rate	Bronkhorst	EL-FLOW Select F-201CV 020-AAD-11K	Serial through USB
**Mass Flow Controller**	H_2 flow-rate	Bronkhorst	EL-FLOW Select F-201CV 020-AAD-11K	Serial through USB
**PID Temperature Controller**	Temperature	Omron	E5GC	Serial through USB
**Light Intensity Controller**	Light Intensity	Developed in-house (see Light intensity control)		Serial through USB
**Back Pressure Regulator**	Pressure	Developed in-house (see Back pressure regulator)		Serial through USB

S5.2 Mass flow controllers

Two EL-FLOW Select Mass Flow Controllers (MFC) model F-201CV were used to supply CO_2 and H_2 to the reactor system. The use of two separate devices allows for a accurate definition of the stoichiometry of the reaction mixture, as well as the overall flow rate and residence time within the reactor. Each MFC was supplied with an RS-232 T-part cable (Bronkhorst article no. 7.03.366), an RS-232 to USB converter (Bronkhorst article no. 9.09.122) and a dedicated plug-in power supply (PiPS, Bronkhorst article no. 7.03.422). This allowed connection with the devices via USB ports on the computer.

Communication with the device was established via RS-232 serial-through-USB using the FLOW-BUS Propar protocol.[4] This protocol was accessed from the Python scripting language[5] using the Bronkhorst-propar module available from Pypi via pip.[6,7]

S5.3 Temperature controller

The oil-tank reactor system used was equipped with an Omron E5GC proportional integral derivative (PID) temperature controller capable of being controlled via RS-485 serial connection using the Modbus protocol.[8] A USB to RS-485 Serial converter cable manufactured by FTDI Ltd was used to connect this device to the computer via a USB port. The PID controller had to be manually set up to communicate via Modbus and further controlled from the Python scripting language via the PyModbus module available from Pypi via pip.[9]

S5.4 Light intensity controller

The light source of the reactor setup was powered by a variable current LED power supply (Figure S25 MeanWell, HLG-480H-36AB). This is equipped with a DIM+/- input which allows to control light intensity by sending a 10V (peak to peak, pk-pk) PWM signal proportional to the desired output power (between 10 and 100%).[10]

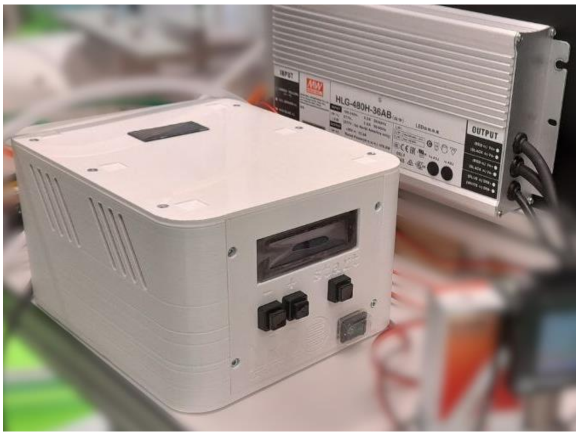

Figure S25 Light intensity control module (left) and LED power supply (right).

The DIM signal is generated by an Arduino UNO microcontroller, which features a built-in PWM generator.[11] Since the microcontroller is only capable of producing 5Vpk-pk, a small custom electronic board featuring an operational amplifier is used to convert the signal to the required amplitude. In addition, two medium power MOSFET transistors are connected, which enable independent control of the cooling fans and light source circuit breaker. Since the light intensity cannot be turned off completely via the DIM signal, the circuit breaker interrupts the power to the entire power supply, ensuring a safe off condition. The control module operates from a dedicated 12V power supply and can be operated with or without an external control computer via the front panel interface.

Serial Communication

The light control module can be controlled by a computer via serial-through-USB taking advantage of the USART module built into in the Arduino UNO board.[11] The microcontroller is also programmed via the same connection. A custom C++ script was developed to allow the control of the light intensity, fans and circuit breaker from the computer. The Arduino UNO firmware for the light control device is available in the repository.

It is recommended to program the device using the Arduino IDE (tested on version 2.1.0), by opening the project files, connecting the Arduino UNO board to the computer and upload the code to it.[12] If the process was successful, opening the Arduino IDE serial monitor and typing the command ‘R1’ followed by the enter key should result in the device responding with its device identifier (by default ‘LIGHT_CONTROL_0’).

All communications between the computer and light control module take place via the following two statements, sent over the serial interface:

- ‘Sx=y’ (Set variable x to value y)
  Sending the ASCII character ‘S’ tells the microcontroller that a variable value needs to be changed. An integer number must follow the ‘S’ character, indicating the variable number (see Table S3). After a delimiter character (any non-digit except ‘.’), the value for the variable must be given. The type depends on the variable.
  Note: in case of a string variable, the command must terminate with a newline.
- ‘Rx’ Read variable x
  Sending the ASCII character ‘R’ tells the microcontroller that a variable value needs to be printed to serial. An integer number must follow the ‘R’ character, indicating the variable number (see Table S3).

Table S3 Variables available for monitoring and control via serial protocol for the light intensity controller module.

Variable number	Access	Type [range]	Description
**0**	RESERVED	-	To avoid undesired errors, this value is not used.
**1**	READ/WRITE	String [20]	**Device identifier**<br>This can be used to distinguish similar serial devices.
**2**	READ/WRITE	Int [0-1]	**Lights On/_Off state**<br>Setting to 1 turns on the lights and the fans. Setting to 0 will turn the lights off only.
**3**	READ/WRITE	Int [0-1]	**Fans On/_Off state**<br>Controls the fans, if the lights are on the fans will not be turned off.
**4**	READ/WRITE	Int [0-1]	**Manual interface lock On/_Off state**<br>Setting this to 1 prevents users from changing the device settings via the manual interface.
**5**	READ/WRITE	Int [0-100]	**Light intensity**<br>Percentage of light intensity for the lights.<br>According to the power supply manufacturer, values below 10% should be avoided. Do not set this to 0 to turn the lights off, use the Lights On/Off variable instead.

To automate the control of this device, the Pyserial module (available from Pypi via Pip) was used within the Python scripting language to be able to generate, send and receive messages via the serial port.[13]

Stand-alone operation

In addition to serial protocol, the device can be controlled from the front panel. The bottom right switch cuts off power to the entire device, including the microcontroller and LED power source. An alphanumeric LCD display provides the user with feedback on the device status and operation. The buttons marked ‘+’ and ‘–’ can be used to select the desired light intensity. To activate the lights, the ‘start’ button must be pressed for 3 seconds. The cooling fans are automatically activated. Pressing the button again for 3 seconds turns the lights off, while the cooling fans will stay on until the device is reset (e.g., turn off main power).

Electrical connections

The electrical connections between the components of the light control module are illustrated in Figure S26. All signal connections were made using Dupont wires, while power supply connections were made with multistrand wires with 0.75 mm^2 cross-section.

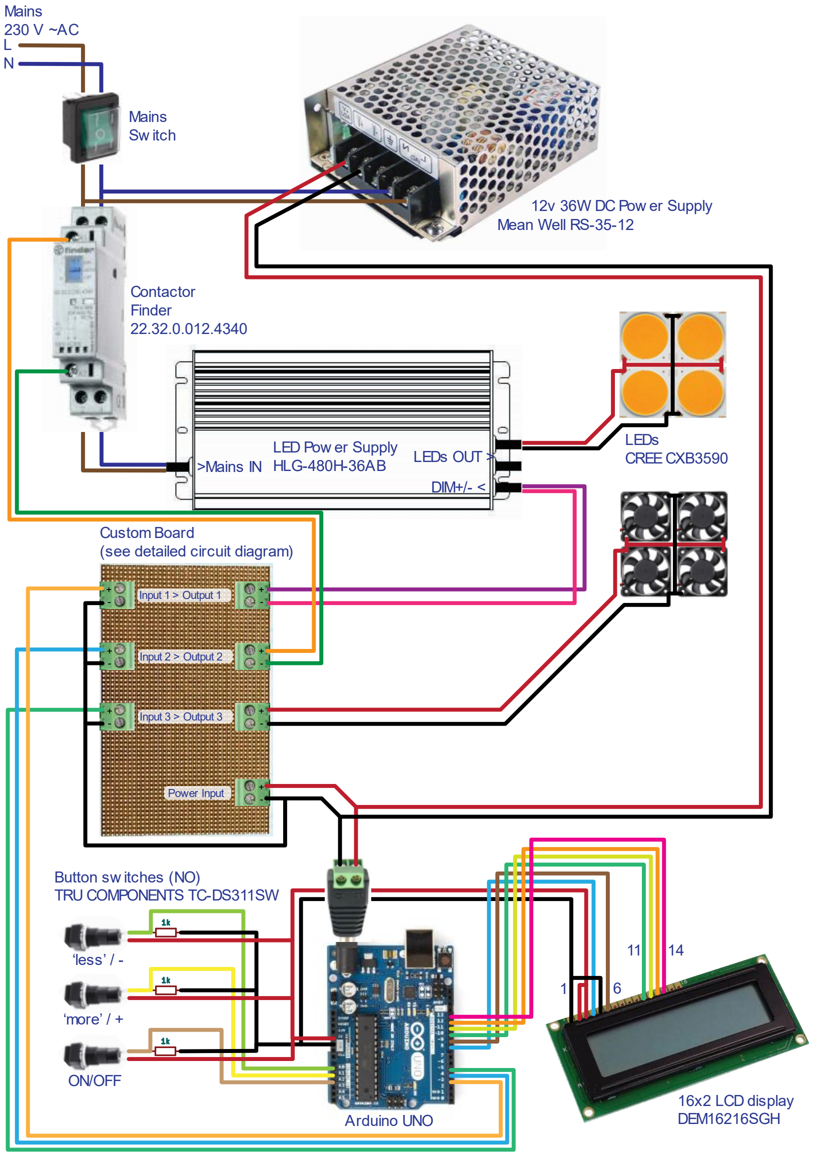

Figure S26 Electrical connections diagram for the light intensity controller module. Where polarity is not specified, black-colored wiring represents a ground line and red-colored wiring represents lines with positive voltage relative to ground.

The custom board was soldered on an Eurocard prototyping PCB with the connections shown in Figure S27.

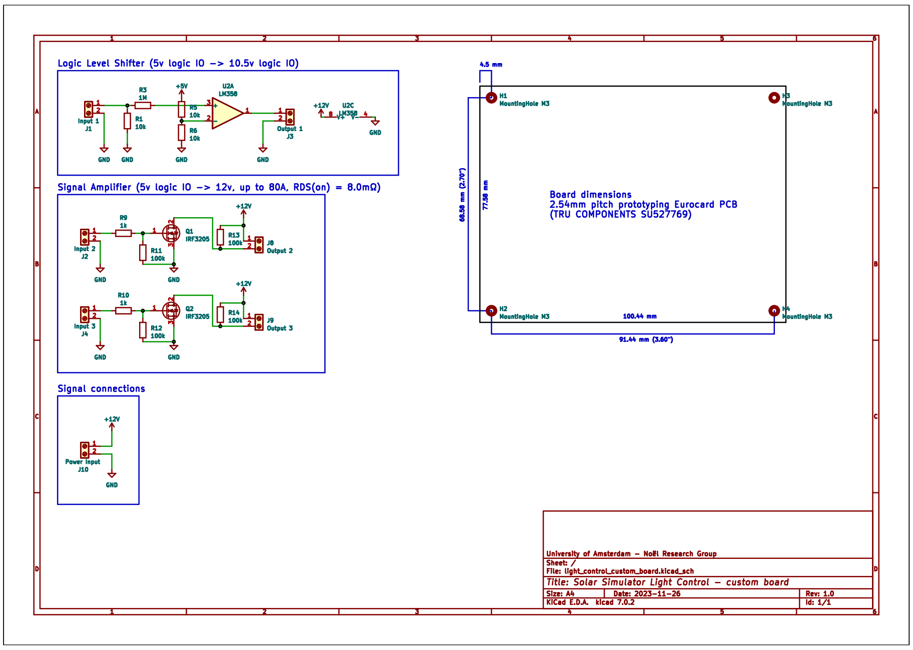

Figure S27 Electrical diagram of the custom board of the light intensity controller.

Mechanical components

Most components of the light control module are housed in a 3D-printed PLA plastic box consisting of 3 main parts held together by 10 M3x12mm cylindrical head DIN 912 screws. The screws are threaded into M3 brass plastic inserts which are embedded into the 3D-printed parts with heat.

The circuit breaker, Arduino board and a custom board are secured to the main box body via adaptors which fit into the rail system at the bottom of the box. All screws used are M3x12mm cylindrical head DIN 912 and all holes are made to fit M3 inserts (5mm OD).

STL files for all parts can be found in the 3D_printed_parts folder within the project repository.

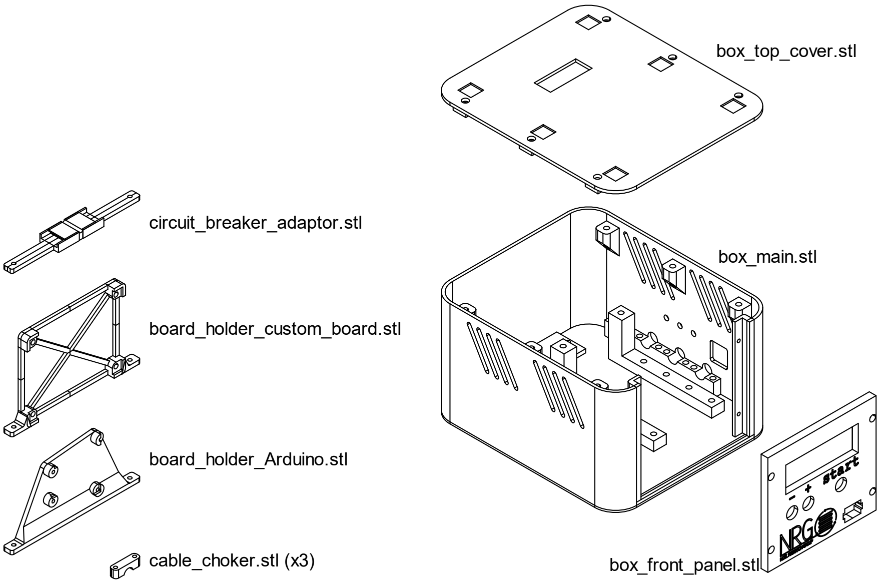

Figure S28 Mechanical components of the light control module.

Bill of Materials

Table S4 Bill of materials for the light control module.

Part	Manufacturer	Model	Amount
**LED Power Supply**	MeanWell	HLG-480H-36AB	1
**12v 36W DC power supply**	Mean Well	RS-35-12	1
**Contactor**	Finder	22.32.0.012.4340	1
**Cooling fans 40mm 12v**	Sunon	MF40101V2-1000U-G99	4
**Press button**	TRU COMPONENTS	TC-DS311SW	3
**LCD display 16x2**	DISPLAY Elektronik GmbH	DEM16216SGH	1
**Arduino UNO**	Arduino	Arduino UNO R3	1
**Mains switch**	Omron	A8L-21-11N2	1
**Screw terminal block 2 contacts**	TE Connectivity	282837-2	7
**1kΩ 1/4W resistor**	-	-	5
**10kΩ 1/4W resistor**	-	-	3
**100kΩ 1/4W resistor**	-	-	4
**1MΩ 1/4W resistor**	-	-	1
**Power MOSFET**	-	IRF3205	2
**Operational amplifier**	-	LM358	1
**Prototyping PCB**	TRU COMPONENTS	SU527769	1
**Brass insert for plastic M3 ⌀5.0mm x 5.0mm**	123-3D	DBM00213	50
**Cylindrical head screw M3x12mm DIN 912**	-	-	50

S5.5 Back pressure regulator

In order to control the pressure within the reactor system, a variable back pressure regulator (BPR) was placed at its outlet before any analysis or reactivity module. Just before the variable BPR, a pressure sensor was connected to the fluid path (Figure S29 and Figure S30).

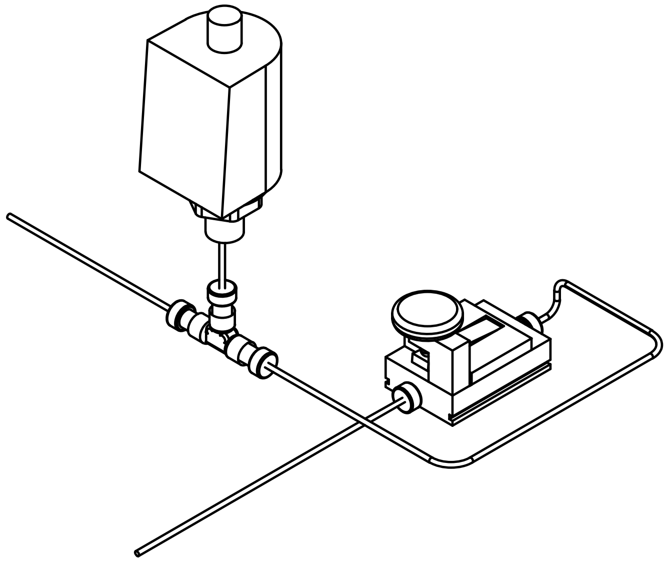

Figure S29 Fluidic paths connections for the pressure sensor (on the top left) and the variable BPR (bottom right). The reactor outlet is connected to the tube in the rear-left, while the analysis or reactivity modules are connected to the tube at the front.

A commercial variable BPR produced by Vapourtec was employed, which can set a determined pressure by turning a knob. The knob was mechanically linked to a stepper motor via an 8 mm axle and a timing belt. Additionally, the axle was connected to a 10 turns precision potentiometer, which allowed the evaluation the absolute position of the valve at any moment. An Arduino microcontroller could operate

the motor while constantly monitoring the pressure at the inlet of the variable BPR, adjusting it through a feedback system to obtain the desired pressure. Reading the pressure is a requirement, as the pressure set has a slight dependency on the flowrate, hence the same knob position results in slightly different pressure values at different flowrates.

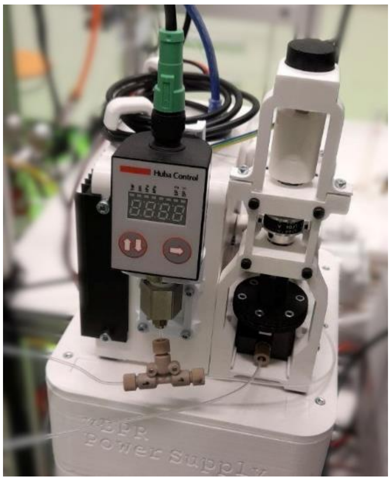

Figure S30 Variable back pressure regulator module with dedicated power supply.

The device was designed to operate under both automated and manual control. In automation, a computer would communicate with the Arduino UNO board to read and set desired pressure values. Automatic control can be remotely enabled or disabled. In the latter case (default when switching on the device) the user has the ability to move the knob by hand and read the pressure from the screen integrated within the sensor. When the automatic control is disabled, the device can still monitor the pressure, which can be logged by the central control unit.

Serial Communication

The variable BPR module can be controlled by a computer via serial-through-USB taking advantage of the USART module built into in the Arduino UNO board.[11] The serial communication takes place with the same modality as for the light intensity controller. The microcontroller is programmed via the same connection. A custom C++ script was developed to allow the control of the pressure from the computer. The Arduino UNO firmware for the variable BPR device is available in the repository.

It is recommended to program the device using the Arduino IDE (tested on version 2.1.0), by opening the project files, connecting the Arduino UNO board to the computer and upload the code to it.[12] If the process was successful, opening the Arduino IDE serial monitor and typing the command ‘R1’ followed by the enter key should result in the device responding with its device identifier (by default ‘PRESSURE_CONTROL_0’).

All communications between the computer and variable BPR module take place via the following two statements, sent over the serial interface:

- ‘Sx=y’ (Set variable x to value y)
  Sending the ASCII character ‘S’ tells the microcontroller that a variable value needs to be changed. An integer number must follow the ‘S’ character, indicating the variable number (see Table S5). After a delimiter character (any non-digit except ‘.’ will do), the value for the variable must be given. The type depends on the variable.
  Note: in case of a string variable, the command must terminate with a newline.
- ‘Rx’ Read variable x
  Sending the ASCII character ‘R’ tells the microcontroller that a variable value needs to be printed to serial. An integer number must follow the ‘R’ character, indicating the variable number (see Table S5).

Table S5 Variables available for monitoring and control via serial protocol for the automated back pressure regulator.

Variable number	Access	Type [range]	Description
**0**	RESERVED	-	Serial.parseInt returns 0 on error<br>To avoid undesired variable access, this value is not used.
**1**	READ/WRITE	String [20]	**Device identifier**<br>This can be used to distinguish similar serial devices.
**2**	READ/WRITE	Int [0-1]	**Device enable On/_Off state**<br>Setting to 1 enables automatic pressure control, 0 disables it. Once the device is enabled, it attempts to achieve the target pressure by moving the back pressure regulator valve. In this state the valve should not be adjusted manually, as this may damage the device and interfere with its operation.<br>If the device is disabled, the valve position can be adjusted manually.
**3**	READ/WRITE	Float	**Pressure setpoint** [Bar, relative]<br>Access this variable to set or read the desired pressure value.
**4**	READ_ONLY	Float	**Pressure measurement** [Bar, relative]<br>Reading this variable provides the latest pressure measured by the device.
**5**	READ_ONLY	Int [0-1024]	**Absolute valve position**<br>Read the absolute position of the valve as an arbitrary number.
**6**	COMMAND	Float	**Begin calibration procedure** [Bar relative]<br>The device reads the pressure as an analogue signal. For this reason, it stores a 2 points calibration curve to convert the signal intensity into the desired units. To set this curve, disable the device (variable 2) and manually bring the system pressure close to the minimum by operating the adjustable valve (e.g., 1.0 bar). Then send this command using the known pressure value in Bar as argument. The accurate pressure value can be read from the screen of the pressure sensor instrument. Note: this command alone will have no effect on the behaviour of the device, as it must be followed by command 7.
**7**	COMMAND	Float	**End calibration procedure** [Bar relative]<br>Stores the second calibration point, causing the calibration data to be changed and stored on the device. Before the command is issued, the valve should be adjusted to achieve a higher pressure than the one recorded for the previously issued command 6.<br>Note: issuing this command before command 6 will result in undefined behaviour.
**8**	COMMAND	None*	**Set current valve position as minimum**<br>Sets the lower limit for the actuation of the valve. The device will not loosen the valve beyond this position. Before issuing the command, the valve should be manually loosened until the pressure reading is zero, or a few turns after that.
**9**	COMMAND	None*	**Set current valve position as maximum**<br>Sets the higher limit for the actuation of the valve. The device will not tighten the valve beyond this position. Before issuing the command, the valve should be manually screwed tightly (as much as it will go without strong resistance), then half a turn back.
**10**	COMMAND	Int	**Move motor**<br>Moves the motor by the specified number of steps (both positive and negative values are allowed, 2560 steps correspond to 1 turn of the valve). The direction of rotation is

			given by the sign of the number. This command should be issued only in the disabled state (S2=0).

** When the type of a variable is None, the command should be issued as 'Sx=' with a newline terminating the string and no argument.*

To automate the control of this device, the Pyserial module (available from Pypi via Pip) was used within the Python scripting language to be able to generate, send and receive messages via the serial port.[13]

Electrical connections

The electrical connections between the components of the variable back pressure regulator are shown in Figure S31. The Arduino microcontroller reads the pressure from the sensor as an analog signal. The analog signal is adjusted to a suitable range for the Arduino UNO (0-5V) and isolated from the sensor power supply with a simple buffer circuit on a small prototyping circuit board. This circuit adapts easily to other pressure sensors, as long as they can produce an analog output, by adjusting the calibration trimmer on the current-to-voltage conversion board and subsequently performing the calibration procedure on the Arduino board (described in variables 6 and 7 of the Serial Communication section).

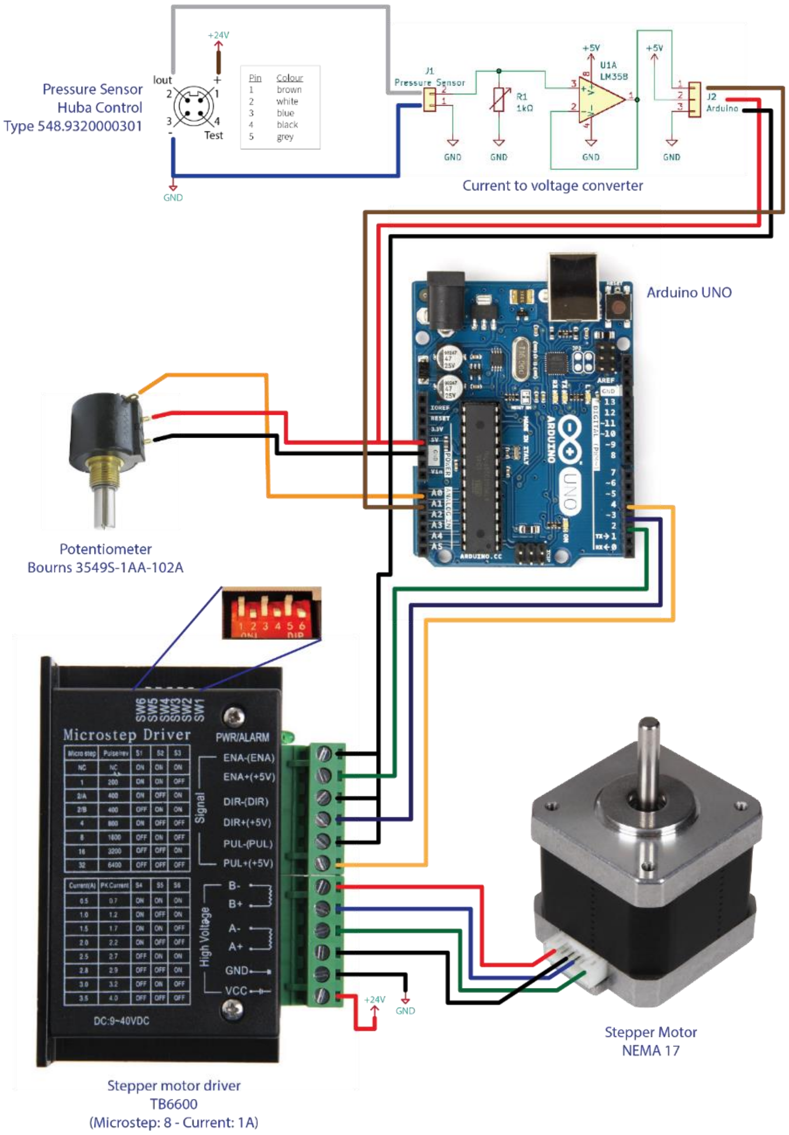

Figure S31 Electrical connections of the variable back pressure regulator module.

Mechanical components

The variable back pressure regulation module is made of two parts: an assembly necessary to mechanically connect the motor, BPR and potentiometer to the same shaft, and a box to hold the other electrical components neatly. Both are made of polylactic acid (PLA) plastic 3D printed parts, with the exception of the 'custom 8mm axle', which was made from steel to ensure rigidity and resistance to mechanical stress. All 3D files are available together with the Arduino code within the repository.

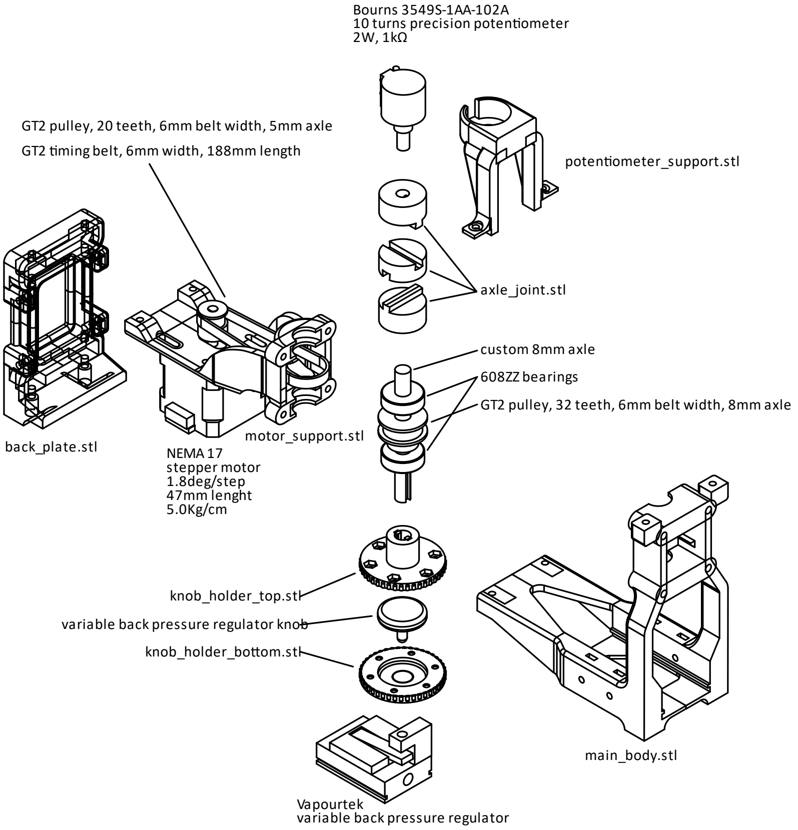

Figure S32 Mechanical components of the drive stack of the variable back pressure regulator module.

The drive shaft is kept in place by two 608ZZ bearings and is moved by the NEMA17 stepper motor via a GT2 timing belt and two pulleys. The knob of the commercial BPR is secured in between two holders, whose connection to the shaft allows a slight vertical motion to allow for the movement of the knob upon screwing and unscrewing. Similarly, a 3-piece axle joint compensates small imprecisions in the horizontal centering of the drive shaft and potentiometer shaft, eliminating mechanical stress upon turning. The rest of the 3D printed parts hold all components in place.

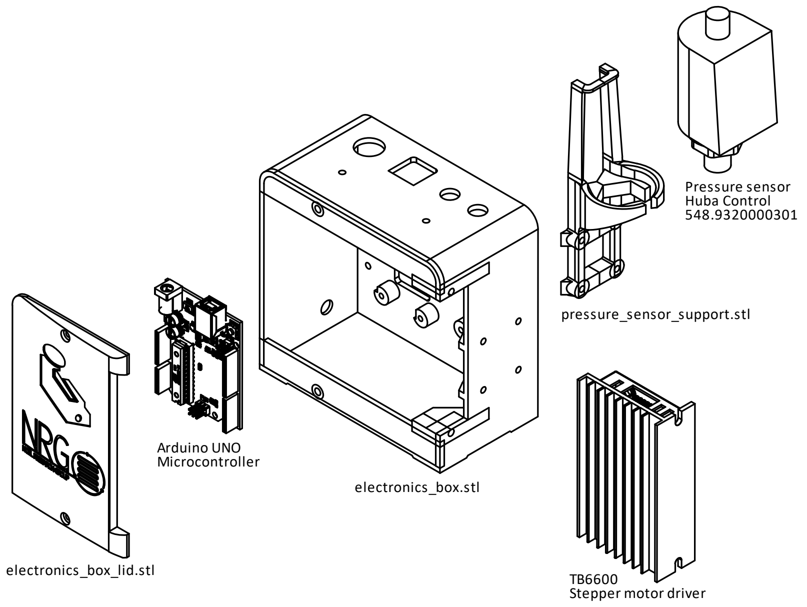

Figure S33 Mechanical and electronic components of the auxiliary box of the variable back pressure regulator module.

Similarly to the light control module, the variable back pressure regulator has an auxiliary 3D-printed box for electronic components and connections, as well as a separate power supply box.

Bill of Materials

Table S6 Bill of materials for the light control module.

Part	Manufacturer	Model	Amount
**10 turns precision potentiometer 2W, 1kΩ**	Bourns	3549S-1AA-102A	1
**Ball bearings 8x22x7mm (ID,OD,height)**	123-3D	608ZZ	2
**GT2 pulley 32 teeth, 6mm, 8mm axle diameter**		DME00107	1
**Back pressure regulation valve**	Vapourtec		1
**NEMA 17 stepper motor, 1.8deg/step, 47mm, 5.0Kg/cm**	123-3D	DMO00052	1
**GT2 timing belt, 6mm width, 188mm length, closed**	123-3D	DME00118	1
**GT2 pulley 20 teeth, 6mm, 5mm axle diameter**	123-3D	DME00051	1
**Stepper driver**	123-3D	TB6600	1
**Pressure sensor**	Huba Control	548.9320000301	1
**Arduino UNO**	Arduino	Arduino UNO R3	1
**Screw terminal block 2 contacts**	TE Connectivity	282837-2	3
**1kΩ 1/4W resistor**	-	-	1
**Operational amplifier**	-	LM358	1
**Prototyping PCB**	TRU COMPONENTS	SU527769	1
**Brass insert for plastic M3 ⌀5.0mm x 5.0mm**	123-3D	DBM00213	50
**Cylindrical head screw M3x12, 20, 30mm DIN 912**	-	-	50

S5.6 System automation monitor

To automatically control the parameters of the reactor system, a modular python graphical user interface (GUI, Figure S34) was developed to communicate with all components of the setup from a centralized computer.

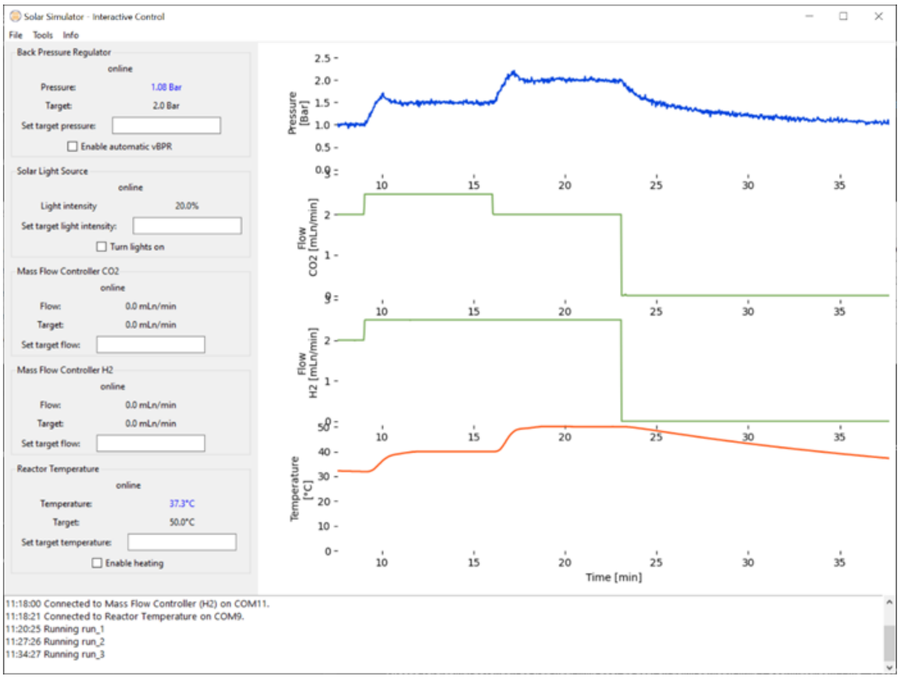

Figure S34 Main window of the system automation monitor controlling the reaction setup.

A main window offers a view of the trend for the key parameters of each device and several panes on the left to manually enable, disable and set a parameter for each device. Additionally, a ‘Tools’ menu gives access to device calibrations and one-time adjustments.

An automation window, accessible from the ‘Tools’ menu, allows the user to set and schedule as many reaction conditions as desired by editing a table and adding values for each parameter, in addition to a reaction duration and a pre-run duration to ensure the system has been purged of the products of the previous reaction (Figure S35).

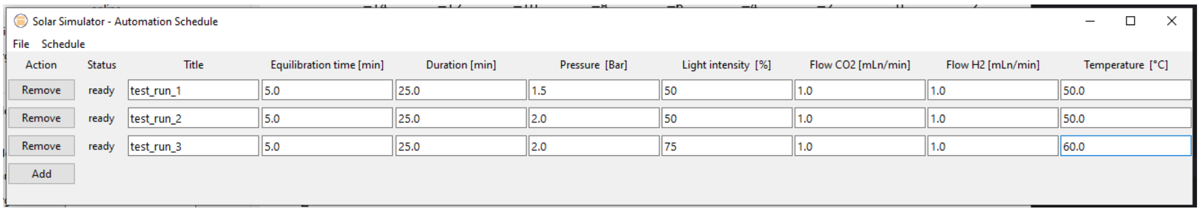

Figure S35 Automation Schedule window of the system automation monitor with 3 test runs setup.

The system automation monitor supports all the devices mentioned earlier, but its modular nature allows to add and modify the supported devices with ease.

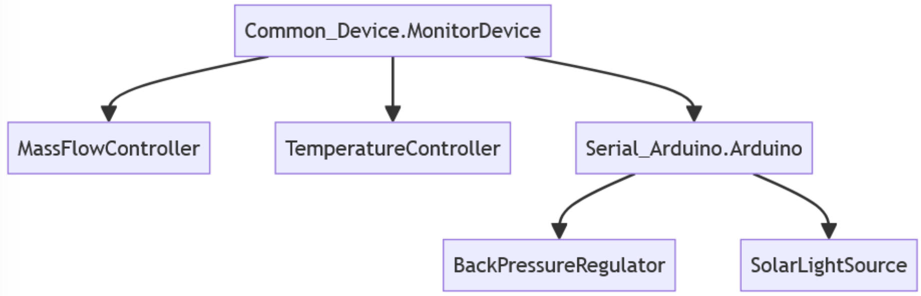

Figure S36 Inheritance structure for the classes controlling the separate devices within the automation monitor.

For each controlled device, a Python class is defined to describe the serial communication methods and provide an interface for the rest of the code. The classes are hierarchically organized with a common

base class and a share base class for the Arduino-based devices developed in-house. The MonitorDevice base class allows all devices to share a common interface and be handled together whenever possible. The Arduino base class implements low level communication with Arduino boards via serial port, leaving only the variable number implementation to the specific classes.

The GUI is based on tkinter,[14] a native Python library which does not add unnecessary overhead and keeps the list of additional requirements to install very low.

Registering a serial device and creating a reaction setup

The program is able to automatically recognize different serial devices by storing several identifiers for each. New devices can be added by running a non-GUI script provided with the code (src/Serial_Devices.py), identifying the device interface in a list and proving a name for it.

Device experimental setups can be created and stored in 'config/device_config.json' by listing the required devices which need to be monitored. The list must specify a name corresponding to a device previously identified and a type, corresponding to the correct class for the device.

S6 Additional results CO and CH_4 production

S6.1 Catalyst screening

Productivities are reported in mmol of compound per weight of catalyst (metal and support, excluding the glass beads) per time. The space time yield (STY) is calculated based on the calculated void volume in the photoreactor. Unless otherwise specified, reactions were carried out without overpressure.

Table S7 Results obtained for the reduction of carbon dioxide with the cobalt(II,III) oxide catalyst.

Oil temper-ature (°C)	Total flow rate (ml/min)	Volumetric ratio CO_2:H_2	Power input lights	Total CO_2 conversion (%)	Selectivity to CH_4 (%)	Productivity (mmol/g_{cat}/h)		STY CH_4 (mmol/ ml/h)
						CH_4	CO	
200	0.5	1:4	100%	32	98	6.2	0.11	0.17
	1	1:4	100%	23	98	8.9	0.19	0.24
	5	1:4	100%	6.9	93	13	0.9	0.36
	1	1:1	100%	6.1	92	5.5	0.49	0.15
160	1	1:4	100%	1.8	96	0.66	0.03	0.02
	1	1:4	0%	1.2	96	0.45	0.02	0.01
250	1	1:4	100%	54	97	21	0.60	0.58
	1	1:4	0%	44	95	17	0.80	0.47

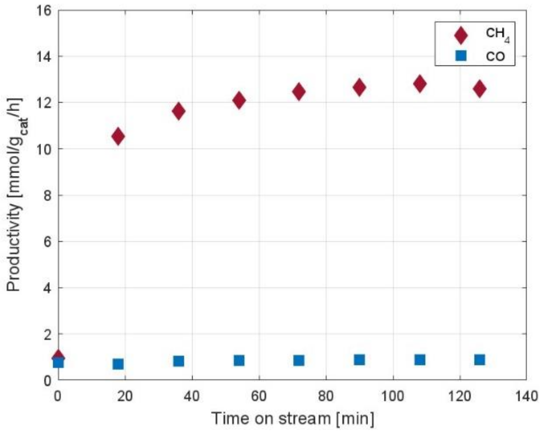

Figure S37 Transient productivity towards methane and carbon monoxide with the cobalt(II,III) oxide catalyst (200 °C, 5 ml/min, CO_2:H_2 1:4, lights on).

Table S8 Results obtained for the reduction of carbon dioxide with the ruthenium nanoparticles on titanium dioxide catalyst. [a] No significant amounts of CO were observed in the analysis.

Oil temper-ature (°C)	Total flow rate (ml/min)	Volumetric ratio CO_2:H_2	Power input lights	Total CO_2 conversion (%)	Selectivity to CH_4 (%)	Productivity (mmol/g_{cat}/h)		STY CH_4 (mmol/ ml/h)
						CH_4	CO[a]	
200	0.5	1:4	100%	70	100	14	0.0	0.38
	1	1:4	100%	48	100	19	0.0	0.51
	1	1:4	0%	35	100	14	0.0	0.38
	5	1:4	100%	11	100	22	0.0	0.59
	1	1:1	100%	14	100	14	0.0	0.38
160	1	1:4	100%	8.9	100	3.5	0.0	0.09
	1	1:4	0%	5.7	100	2.3	0.0	0.06
250	1	1:4	100%	93	100	37	0.0	1.00
	1	1:4	0%	87	100	35	0.0	0.94

	12.5	1:4	100%	26	100	127	0.0	3.43

Table S9 Results obtained for the reduction of carbon dioxide with the ruthenium nanoparticles on strontium titanate catalyst.

Oil temper-ature (°C)	Total flow rate (ml/min)	Volumetric ratio CO_2:H_2	Power input lights	Total CO_2 conversion (%)	Selectivity to CO (%)	Productivity (mmol/g_{cat}/h)		STY CO (mmol/ ml/h)
						CH_4	CO	
200	0.5	1:1	100%	1.9	38	0.59	0.37	0.01
	1	1:1	100%	1.3	64	0.48	0.86	0.02
	1	1:1	0%	1.0	69	0.31	0.69	0.02
	5	1:1	100%	0.64	71	0.93	2.3	0.06
	1	1:4	100%	0.47	66	0.40	0.78	0.02
160	1	1:1	100%	0.26	67	0.09	0.18	0.00
	1	1:1	0%	0.15	60	0.06	0.10	0.00
250	1	1:1	100%	5.7	42	3.4	2.4	0.06
	1	1:1	0%	3.9	51	2.0	2.0	0.05

Table S10 Results obtained for the reduction of carbon dioxide with the gold nanoparticles on titanium dioxide catalyst. [a] No significant amounts of CH_4 were observed in the analysis

Oil temper-ature (°C)	Total flow rate (ml/min)	Volumetric ratio CO_2:H_2	Power input lights	Total CO_2 conversion (%)	Selectivity to CO (%)	CO productivity (mmol/g_{cat}/h)		STY CO (mmol/ ml/h)
						CH_4[a]	CO	
200	0.5	1:1	100%	5.9	100	0.0	2.5	0.10
	1	1:1	100%	4.4	100	0.0	3.1	0.23
	1	1:1	50%	4.1	100	0.0	2.9	0.22
	1	1:1	0%	3.8	100	0.0	2.7	0.20
	5	1:1	100%	1.7	100	0.0	7.3	0.29
160	1	1:1	100%	0.73	100	0.0	0.62	0.02
	1	1:1	0%	0.52	100	0.0	0.45	0.02
250	1	1:1	100%	11	100	0.0	9.5	0.38
	1	1:1	0%	9.9	100	0.0	8.4	0.34
**1.0 bar overpressure**								
200	1	1:1	100%	6.2	100	0.0	4.4	0.33
**2.0 bar overpressure**								
200	1	1:1	100%	7.5	100	0.0	5.3	0.40

S6.2 Control experiments

Table S11 Results obtained for the reduction of carbon dioxide without any catalyst (only glass beads).

Oil temperature (°C)	Total flow rate (ml/min)	Volumetric ratio CO_2:H_2	Power input lights	Total CO_2 conversion (%)	Selectivity to CH_4 (%)	Productivity (mmol/g_{cat}/h)
250	1	1:4	100%	0	-	0

All catalysts have been tested with a feed of pure hydrogen under typical reaction conditions (200 °C, 1 ml_n/min), where for none of the catalysts carbon containing products were detected in the analysis, supporting that the carbon dioxide fed into the reactor is reduced.

S6.3 Catalyst performance in literature

Table S12 The performance of a variety of catalysts under specified conditions.

**Catalyst**	**Temperature (°C)**	**Total flow rate (ml/min)**	**Volumetric ratio CO_2:H_2**	**Light source**	**Productivity (mmol/g_{cat}/h)**	**Ref.**
Iridium on MOF	250	20	1:4	300W Xe 2.4 W/cm^2	CH_4 20	[15]
Nickel on silica/aluminum oxide	225	5	1:4	LED max. 2200 mW/cm^2	CH_4 54	[16]
Ruthenium on aluminum oxide	218	11	1:4.5	1440 mW/cm^2	CH_4 79	[17]
Ruthenium on strontium titanate	150	25	1:4	300 W Xe 0.1 W/cm^2	CH_4 15	[18]
Platinum/ Molybdenum on silica	200	15	1:1	Dolan-Jenner visible light source	CO 1.6 (Conversion rate)	[19]
Rhodium on titanium dioxide	250	20	1:4	300 W Xe 2.7 W/cm^2	CO 21	[20]
Gold on titanium dioxide	200	135	4:1	1440 mW/cm^2	CO 33	[21]

S6.4 Catalyst deactivation

Deactivation under continuous-flow conditions can be tested by running the same conditions over elongated times. During the parameter screening deactivation could be observed over the full time frame (typically more than 10 hours), depending on the catalyst (always below 20% for the targeted compound), showing a qualitative screening where promising conditions are pinpointed for further investigations. During the operation of the gold nanoparticles on titanium dioxide, it was found to be crucial not to cool down the system under reaction conditions (especially with hydrogen) as this led to severe deactivation of the catalyst, hypothesized to be caused by the formation of liquid water at lower temperatures. Ruthenium nanoparticles on strontium titanite showed a decreasing productivity of methane with elongated operation times, causing a varying selectivity towards carbon monoxide.

S7 Additional results carbonylative cross-coupling

S7.1 Carbonylative Suzuki coupling

Table S13 The effect of the controlled addition of boronic acid, solvent system (volume ratio), flow rate and CO:H₂ volume ratio on the Suzuki carbonylation reaction.

Entry	Reactor	Differing conditions	Flow rate (ml/min)	Time (h)	Conversion (%)	Selectivity (%)	Yield (%)
**1**	Batch	-	-	2	76	47	36
**2**	Batch	$Pd(Br)_2$ and tpp	-	2	50	82	43
**3**	Fed-batch	-	0.05	2	65	96	62
**4**	Fed-batch	Dioxane:THF* 98:2	0.05	2	54	98	53
**5**	Fed-batch	Dioxane:THF* 98:2	0.025	2	57	93	53
**6**	Fed-batch	Dioxane:THF* 98:2	0.0125	3	94	99	93 (82)[a]
**7**	Fed-batch	Dioxane:THF* 98:2 $CO:H_2$ 1:9	0.0125	3	63	8	5
**8**[b]	Fed-batch	Dioxane:THF* 98:2 $CO:H_2$ 1:9	0.005	4	56	43	24
**9**[b]	Fed-batch	Dioxane:THF* 98:2 $CO:H_2$ 1:9	0.004	5	54	53	29
**10**[b]	Fed-batch	Dioxane:THF* 98:2 $CO_2:H_2$ 1:1	0.004	6	>99	0	0
**11**[b]	Fed-batch Connected to modular photoreactor	Dioxane:THF* 98:2 Gas mixture 7.5 mol% Pd catalyst, Xantphos	0.0028	6 + 1[c]	97	95	92 (79)[a]

*Reaction conditions: 4-iodoanisole (0.5 mmol), 4-fluorophenylboronic acid (1.5 eq.), $Pd(OAc)_2$ (5%), Xantphos (5%), K_2CO_3 (3 eq.), 3 ml dioxane at 80 °C. Conversion, selectivity and yield were calculated by GC-MS (qualitative, area based). The theoretical yields were checked by ^{19}F-qNMR using fluorobenzene as standard. [a] isolated yield. [b] performed using 0.1 mmol of 4-iodoanisole (all other reagents scaled accordingly). [c] Addition over 6 hours, 7 hours of reaction. * Ratio after addition, THF was added to increase the solubility of the boronic acid.*

S7.2 Alkoxycarbonylation

Table S14 Alkoxylcarbonylation reaction performed with different CO:H₂ volume ratios and with the modular photoreactor system as CO source.

Entry	Reactor	Differing conditions	Flow rate (ml/min)	Time (h)	Conversion (%)	Selectivity (%)	Yield (%)
**1**	Batch	-	-	3	82	100	82 (68)[a]
**2**	Batch	$CO:H_2$ 1:9	-	3	19	21	4
**3**[b]	Fed-batch Connected to modular photoreactor	5 mol% Pd catalyst, dppp	0.0028	6 + 0.5[c]	98	69	68 (61)[a]

Reaction conditions: [1,1'-biphenyl]-4-yl trifluoromethanesulfonate (0.5 mmol), methanol (50 eq.), $Pd(OAc)_2$ (3%), dppp (3%), TEA (3 eq.), 3 ml dioxane at 70 °C. Conversion, selectivity and yield were calculated by GC-MS (qualitative, area

based). [a] isolated yield. [b] performed using the addition of 0.1 mmol of [1,1'-biphenyl]-4-yl trifluoromethanesulfonate. [c] Addition over 6 hours, 6.5 hours of reaction.

S7.3 Aminocarbonylation

Table S15 Aminocarbonylation reaction performed with different CO:H_2 volume ratios and with the modular photoreactor system as CO source.

Entry	Reactor	Differing conditions	Flow rate (ml/min)	Time (h)	Conversion (%)	Selectivity (%)	Yield (%)
**1**	Batch	-	-	3	>99	90	90 (78)[a]
**2[b]**	Fed-batch Connected to modular photoreactor	10 mol% Pd catalyst, tpp	0.0035	5 + 0.5[c]	>99	82	82 (72)[a]

Reaction conditions: (E)-1-iodooct-1-ene (0.5 mmol), 1-hexylamine (2.5 eq.), $Pd_2(DBA)_3$ (5%), tpp (5%), TEA (3 eq.), 3 ml dioxane at 80 °C. Conversion, selectivity and yield were calculated by GC-MS (qualitative, area based). [a] isolated yield. [b] performed using the addition of 0.1 mmol of (E)-1-iodooct-1-ene. [c] Addition over 5 hours, 5.5 hours of reaction.

S8 References

1. Rosas Vargas, D. & Cook, S. P. Palladium nanoparticles: Chemoselective control for reductive Heck with aryl triflates and 2,3-dihydrofuran. *Tetrahedron* **74**, 3314–3317 (2018).

2. Bhattacherjee, D. *et al.* Advances in Transition-Metal Catalyzed Carbonylative Suzuki-Miyaura Coupling Reaction: An Update. *Adv. Synth. Catal.* **363**, 1597–1624 (2021).

3. Shah, R. K. & London, A. L. *Laminar Flow Forced Convection in Ducts*. (Elsevier, 1978). doi:10.1016/C2013-0-06152-X.

4. Retreived from; https://www.bronkhorst.com/int/products/gas-flow/el-flow-select/f-201cv/.

5. Retreived from; https://www.python.org/.

6. Retreived from; https://bronkhorst-propar.readthedocs.io/en/latest/introduction.html.

7. Retreived from; https://pypi.org/project/pip/.

8. Retreived from; https://assets.omron.eu/downloads/manual/en/h174_e5_c_users_manual_en.pdf.

9. Retreived from; https://pymodbus.readthedocs.io/en/latest/.

10. Retreived from; https://www.meanwell-web.com/content/files/pdfs/productPdfs/MW/HLG-480H/HLG-480H-36AB.pdf.

11. Retreived from; https://docs.arduino.cc/resources/datasheets/A000066-datasheet.pdf.

12. Retreived from; https://www.arduino.cc/en/software.

13. Retreived from; https://pypi.org/project/pyserial/.

14. Retreived from; https://docs.python.org/3/library/tkinter.html#.

15. Tang, Y. *et al.* Encapsulating Ir nanoparticles into UiO-66 for photo-thermal catalytic CO_2 methanation under ambient pressure. *J. Mater. Chem. A* **10**, 12157–12167 (2022).

16. Bueno-Alejo, C. J., Arca-Ramos, A., Hueso, J. L. & Santamaria, J. LED-driven continuous flow carbon dioxide hydrogenation on a nickel-based catalyst. *Catal. Today* **355**, 678–684 (2020).

17. Rohlfs, J. *et al.* Continuous-Flow Sunlight-Powered CO_2 Methanation Catalyzed by γ-Al_2O_3-Supported Plasmonic Ru Nanorods. *Catalysts* **12**, 126 (2022).

18. Mateo, D., Albero, J. & García, H. Titanium-Perovskite-Supported RuO_2 Nanoparticles for Photocatalytic CO_2 Methanation. *Joule* **3**, 1949–1962 (2019).

19. Ro, I. *et al.* Measurement of intrinsic catalytic activity of Pt monometallic and Pt-MoO_x interfacial sites over visible light enhanced $PtMoO_x/SiO_2$ catalyst in reverse water gas shift reaction. *J. Catal.* **344**, 784–794 (2016).

20. Tang, Y. *et al.* Photo-Assisted Catalytic CO_2 Hydrogenation to CO with Nearly 100% Selectivity over Rh/TiO_2 Catalysts. *Energy & Fuels* **37**, 539–546 (2023).

21. Martínez Molina, P. *et al.* Sunlight Powered Continuous Flow Reverse Water Gas Shift Process Using a Plasmonic Au/TiO_2 Nanocatalyst. *Chem. – An Asian J.* **18**, e202300405 (2023).

S9 Characterization data

(4-Fluorophenyl)(4-methoxyphenyl)methanone

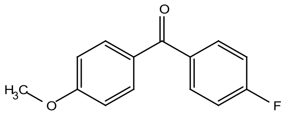

Produced according to section Carbonylative cross-coupling reactions.

^{1}H-NMR (300 MHz, $CDCl_3$): δ 7.85 – 7.74 (m, 4H), 7.15 (t, J = 8.6 Hz, 2H), 6.97 (d, J = 8.9 Hz, 2H), 3.90 (s, 3H).
^{19}F-NMR (282 MHz, $CDCl_3$): δ -106.94 (m).
^{13}C-NMR (75 MHz, $CDCl_3$): δ 194.3, 166.9, 163.5, 163.4, 134.6, 134.6, 132.6, 132.5, 132.4, 130.2, 115.6, 115.3, 113.8, 55.7.

[1,1'-Biphenyl]-4-yl trifluoromethanesulfonate

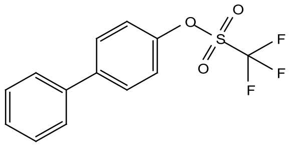

Produced according to ref[1].

^{1}H-NMR (300 MHz, $CDCl_3$): δ 7.66-7.63 (m, 2H), 7.59 – 7.53 (m, 2H), 7.51 – 7.43 (m, 2H), 7.43 – 7.38 (m, 1H), 7.36-7.34 (m, 2H).
^{19}F-NMR (282 MHz, $CDCl_3$): δ -72.77.
^{13}C-NMR (75 MHz, $CDCl_3$): δ 149.1, 141.9, 139.5, 129.1 (2C), 129.0 (2C), 128.2, 127.3 (2C), 121.8 (2C), 118.9 (q, J = 320.7 Hz)

Methyl [1,1'-biphenyl]-4-carboxylate

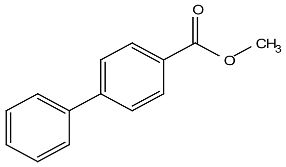

Produced according to section Carbonylative cross-coupling reactions.

^{1}H-NMR (300 MHz, $CDCl_3$): δ 8.17 – 8.05 (m, 2H), 7.71 – 7.57 (m, 4H), 7.52 – 7.35 (m, 3H), 3.94 (s, 3H).
^{13}C-NMR (75 MHz, $CDCl_3$): δ 167.2, 145.8, 140.2, 130.2 (2C), 129.1 (2C), 129.0, 128.3, 127.4 (2C), 127.2 (2C), 52.3.

(*E*)-N-hexylnon-2-enamide

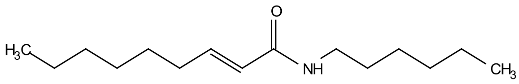

Produced according to section Carbonylative cross-coupling reactions.

^{1}H-NMR (300 MHz, $CDCl_3$): δ 6.82 (dt, J = 15.3, 6.9 Hz, 1H), 5.73 (dt, J = 15.3, 1.6 Hz, 1H), 5.37 (bs, 1H), 3.31 (td, J = 7.2, 5.8 Hz, 2H), 2.16 (qd, J = 7.1, 1.6 Hz, 2H), 1.55-138 (m, 4H), 1.37-1.23 (m, 12 H), 0.95 – 0.81 (m, 6H).
^{13}C-NMR (75 MHz, $CDCl_3$): δ 166.2, 144.9, 123.7, 39.7, 32.2, 31.8, 31.6, 29.8, 29.0, 28.4, 26.8, 22.7 (2C), 14.2 (2C).

S10 NMR and Mass Spectra

(4-fluorophenyl)(4-methoxyphenyl)methanone

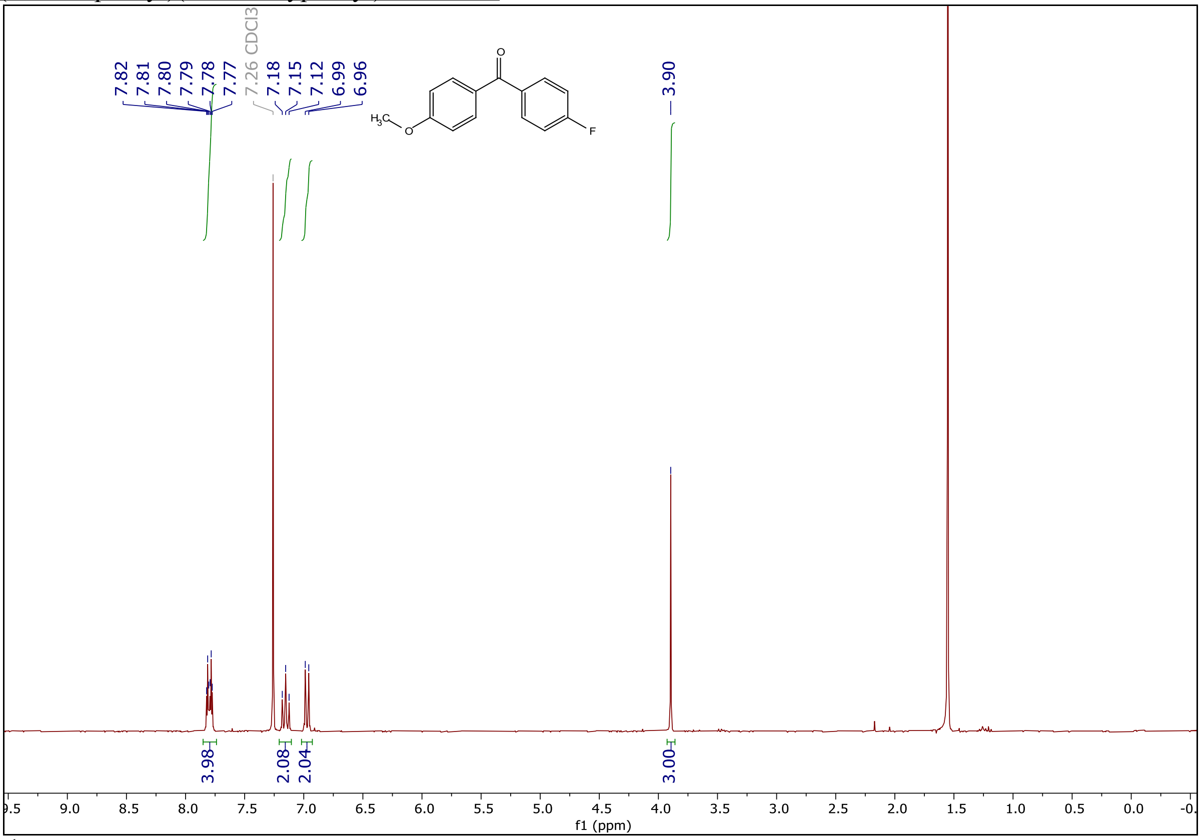

[1]**H-NMR** (300 MHz, $CDCl_3$): δ 7.85 – 7.74 (m, 4H), 7.15 (t, J = 8.6 Hz, 2H), 6.97 (d, J = 8.9 Hz, 2H), 3.90 (s, 3H).

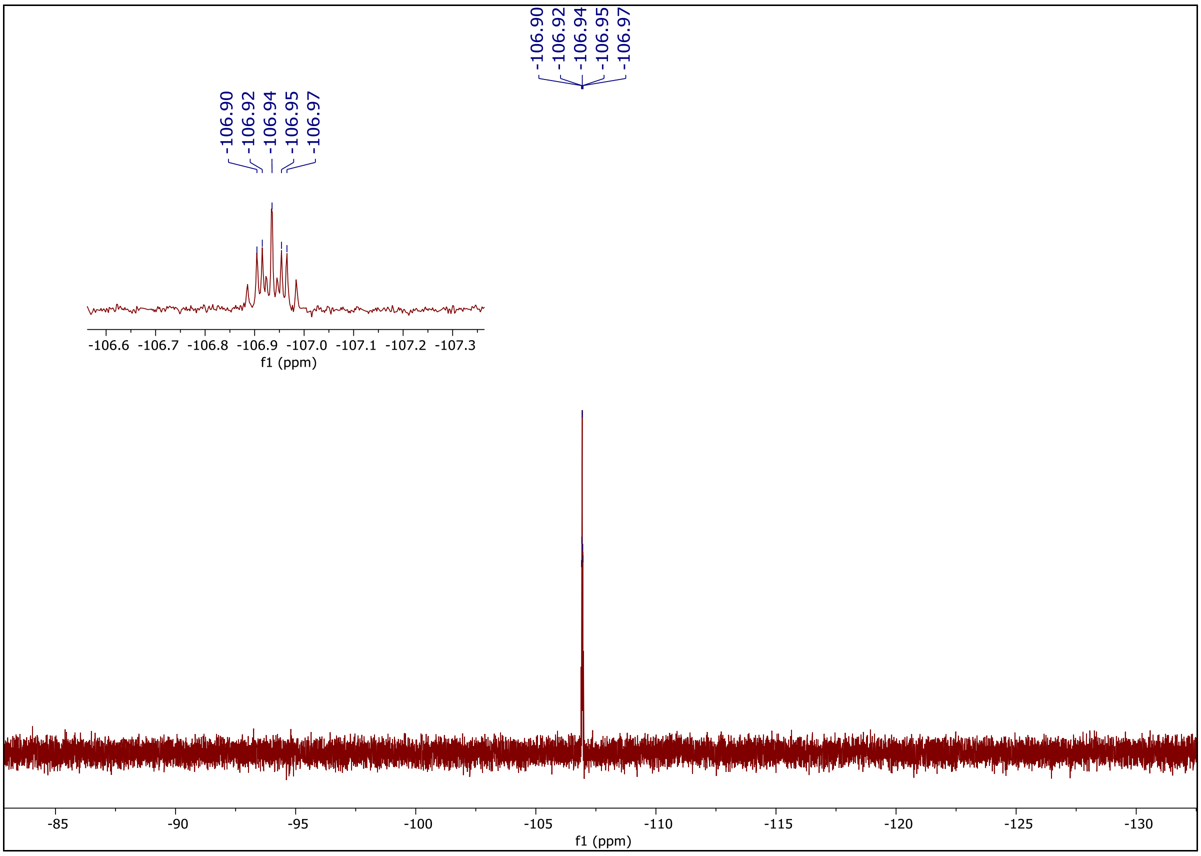

^{19}F-NMR (282 MHz, $CDCl_3$): δ -106.94 (m).

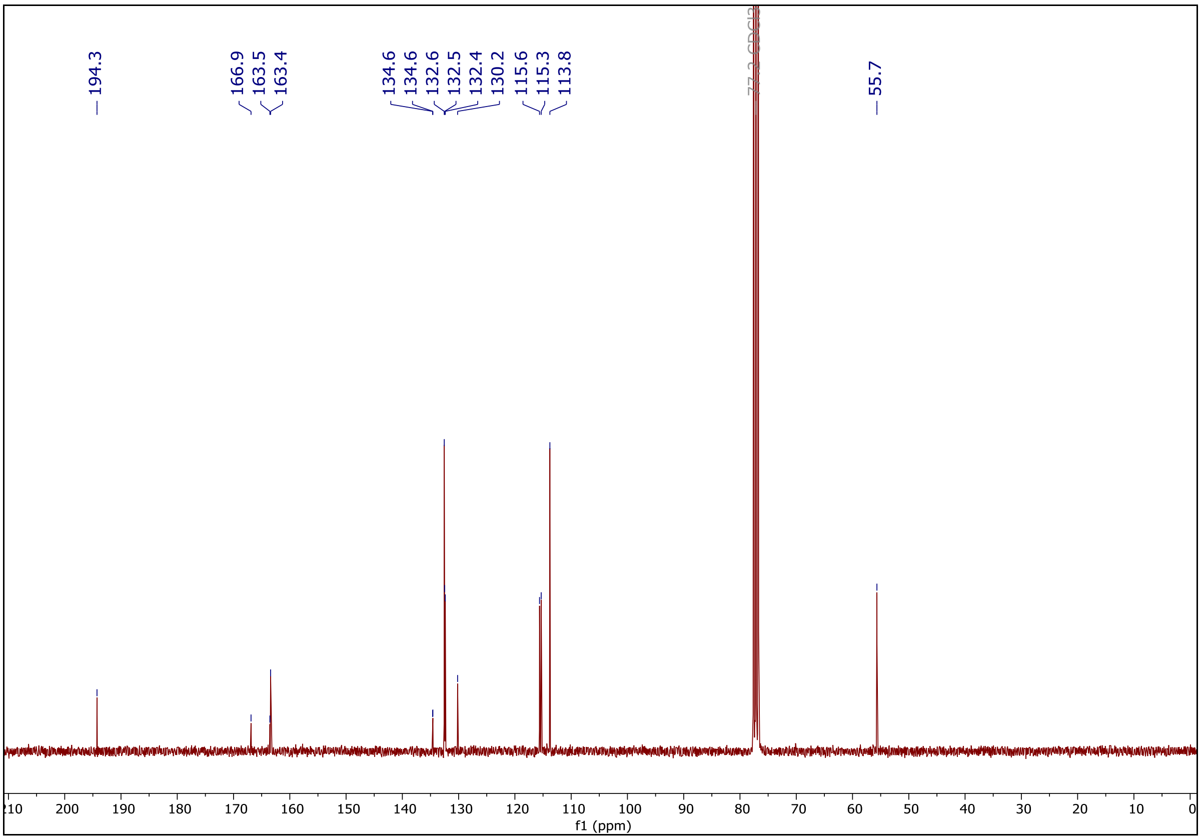

^{13}C-NMR (75 MHz, $CDCl_3$): δ 194.3, 166.9, 163.5, 163.4, 134.6, 134.6, 132.6, 132.5, 132.4, 130.2, 115.6, 115.3, 113.8, 55.7.

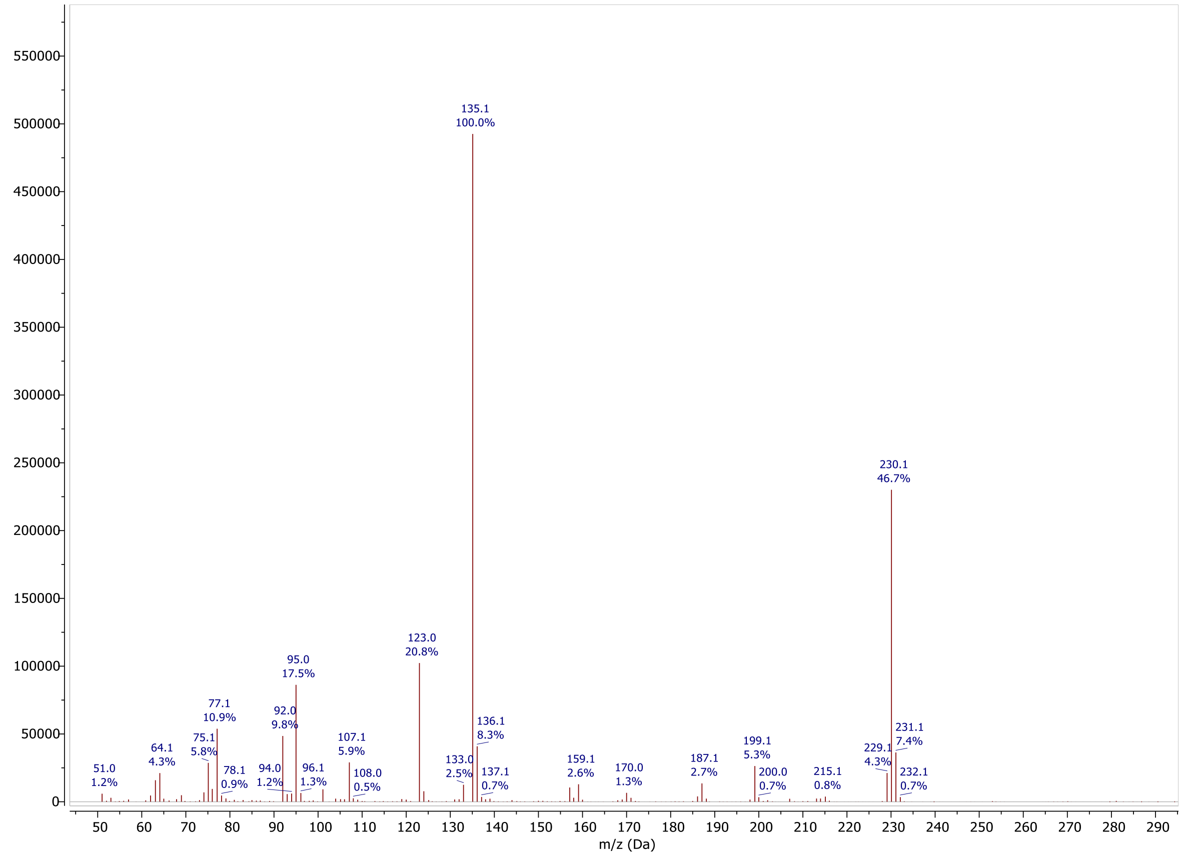

EI-MS: m/z calcd. for (4-fluorophenyl)(4-methoxyphenyl)methanone [M]+ : 230.1, found 230.1.

[1,1'-Biphenyl]-4-yl trifluoromethanesulfonate

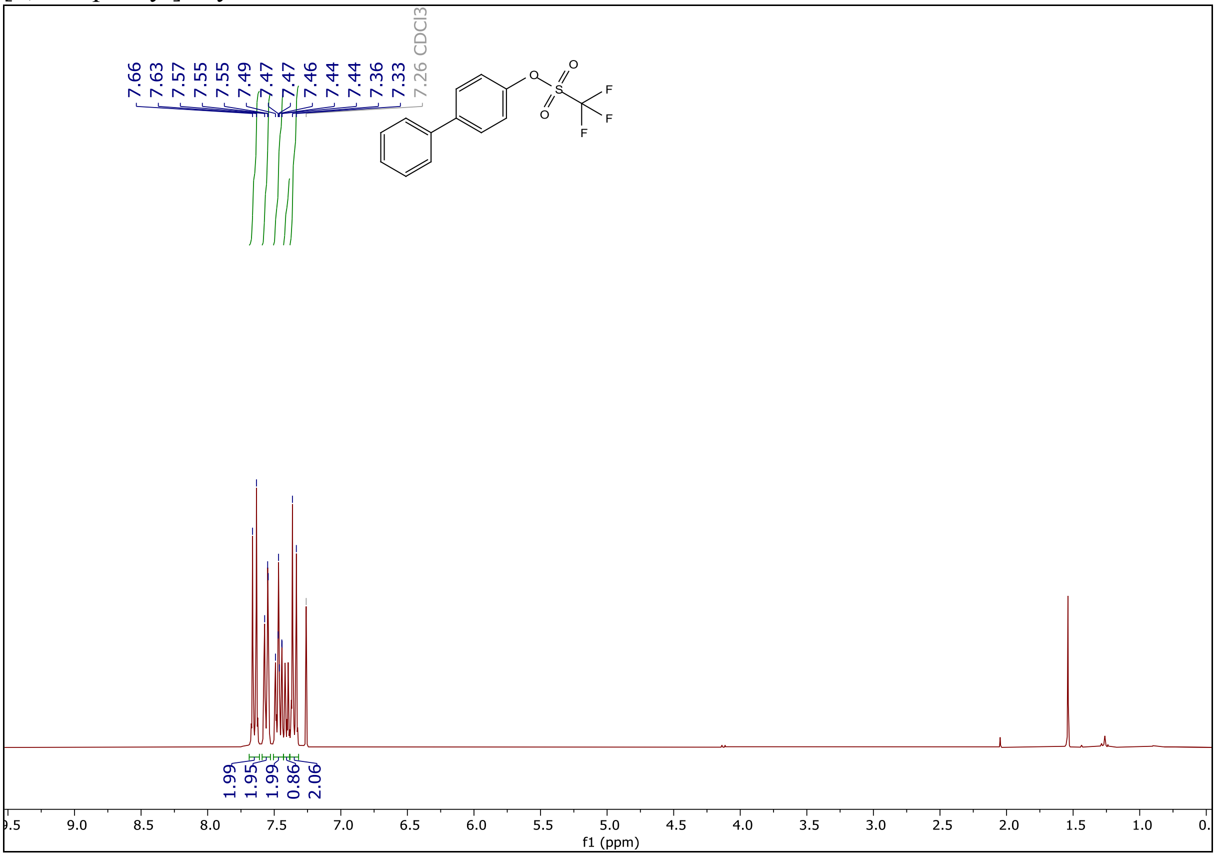

^{1}H-NMR (300 MHz, $CDCl_3$): δ 7.66-7.63 (m, 2H), 7.59–7.53 (m, 2H), 7.51–7.43 (m, 2H), 7.43–7.38 (m, 1H), 7.36-7.34 (m, 2H).

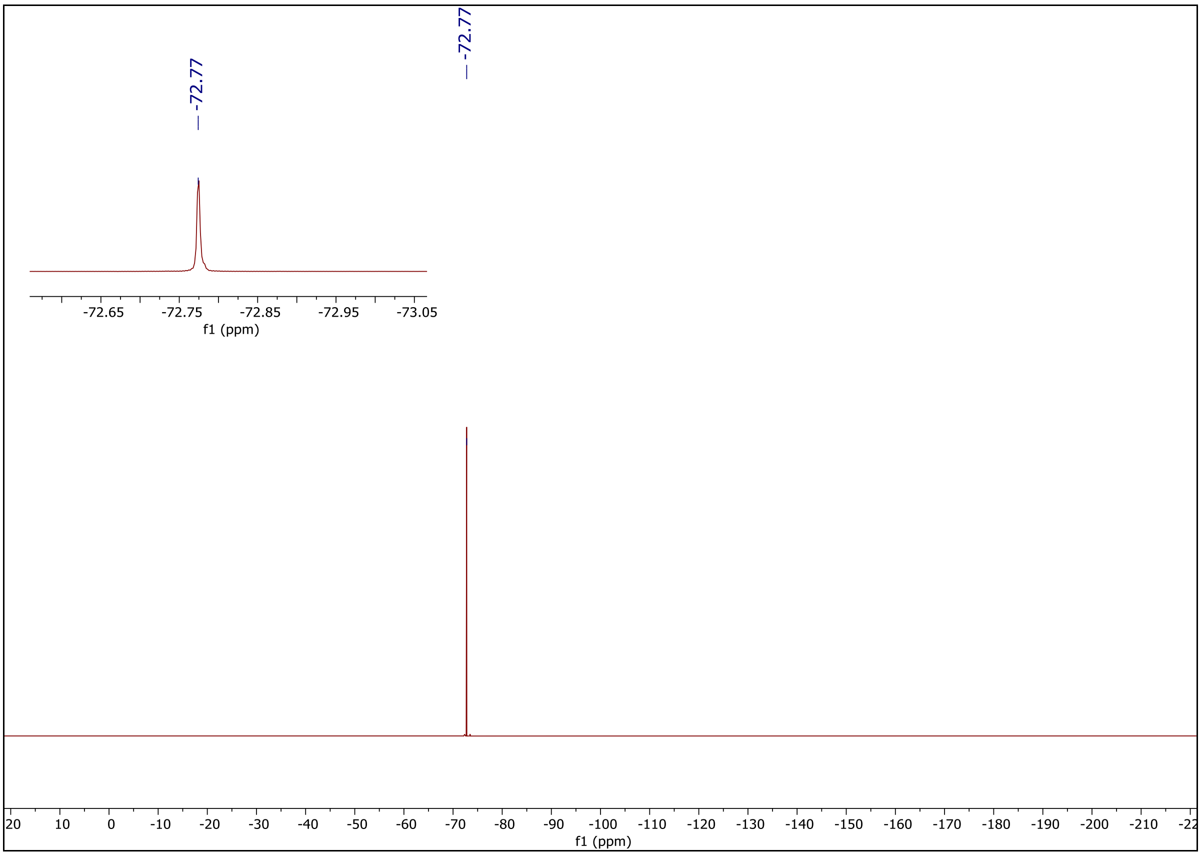

¹⁹F-NMR (282 MHz, $CDCl_3$) δ -72.77.

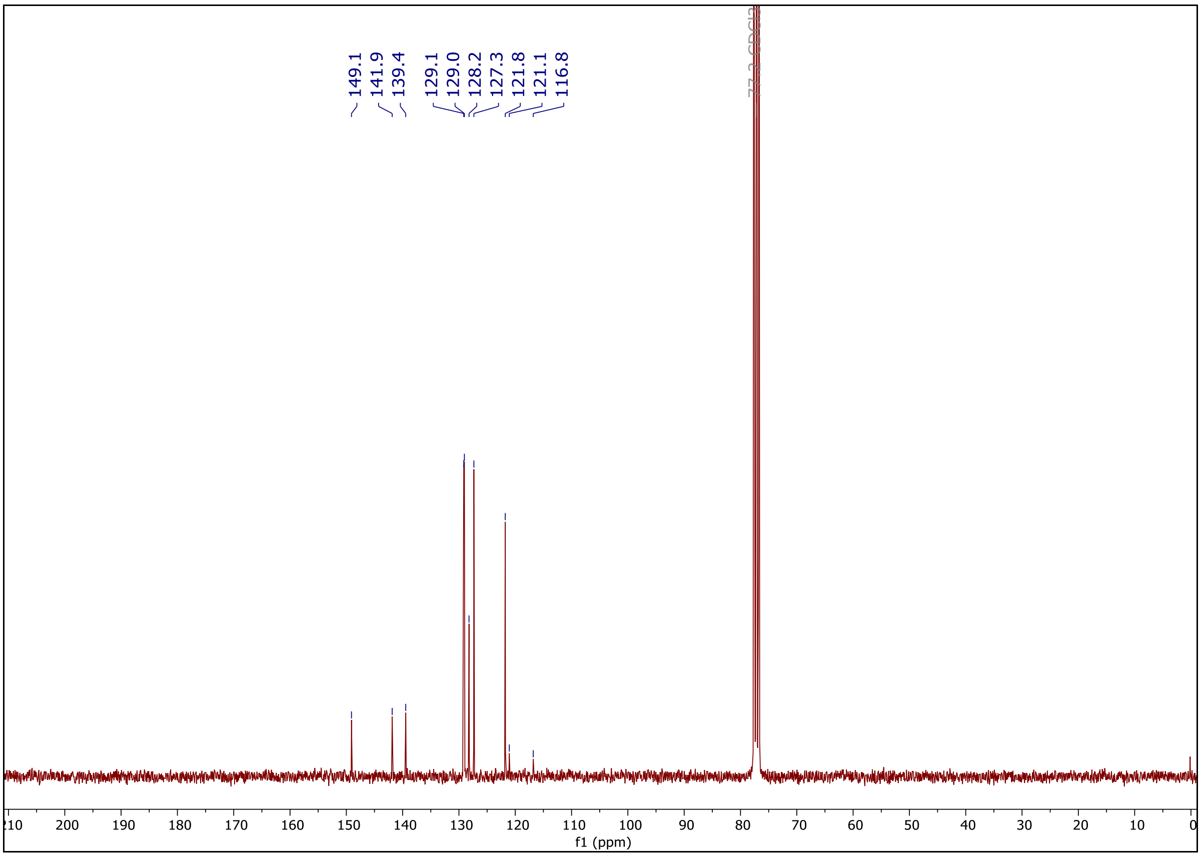

¹³C-NMR (75 MHz, $CDCl_3$) δ 149.1, 141.9, 139.5, 129.1 (2C), 129.0 (2C), 128.2, 127.3 (2C), 121.8 (2C), 118.9(q, J = 320.7Hz)

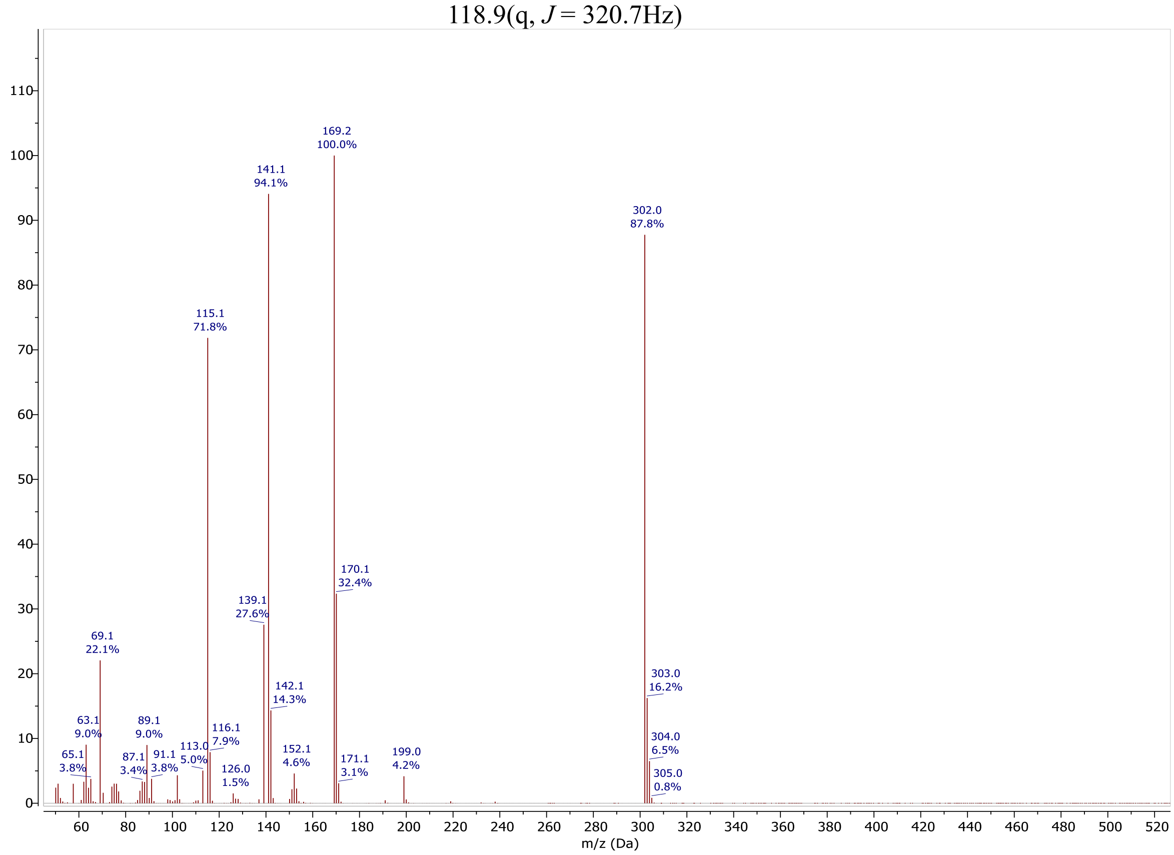

EI-MS: m/z calcd. for [1,1'-Biphenyl]-4-yl trifluoromethanesulfonate [M]+ : 302.0, found 302.0.

methyl [1,1'-biphenyl]-4-carboxylate

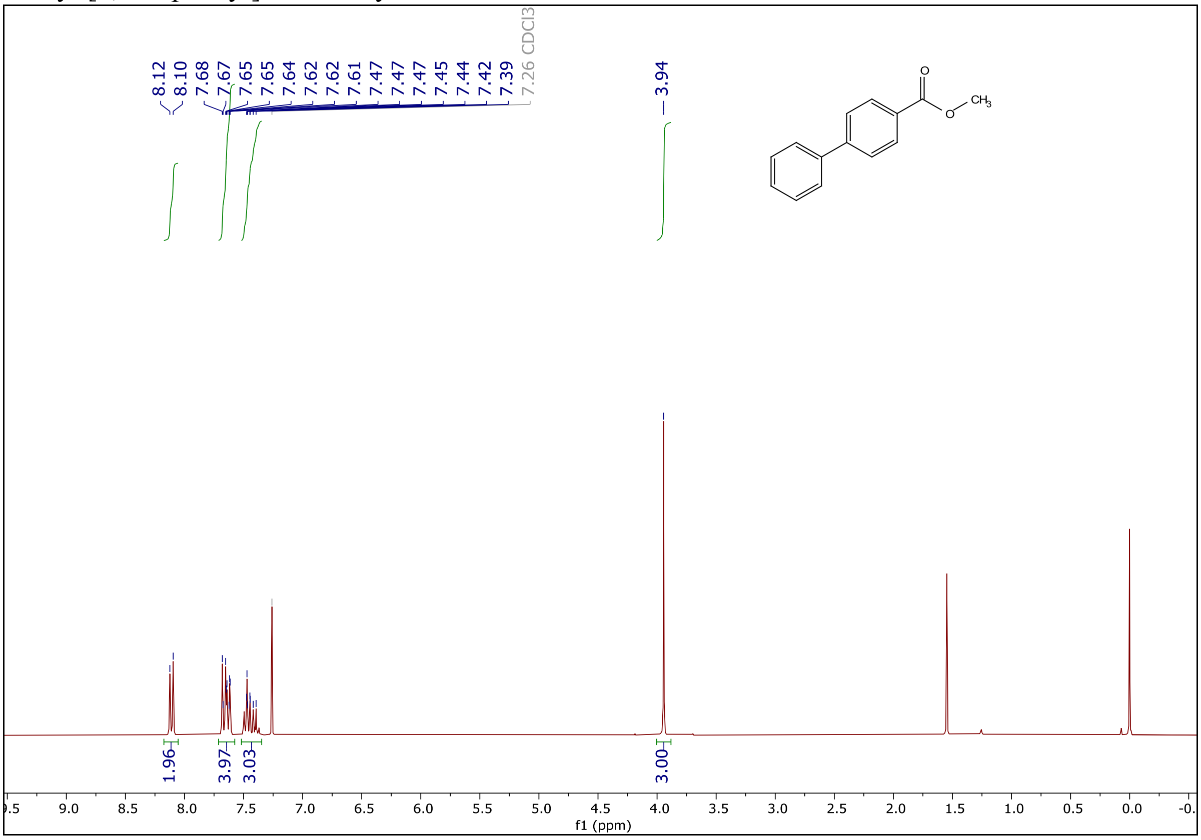

^{1}H-NMR (300 MHz, $CDCl_3$) δ 8.17 – 8.05 (m, 2H), 7.71 – 7.57 (m, 4H), 7.52 – 7.35 (m, 3H), 3.94 (s, 3H).

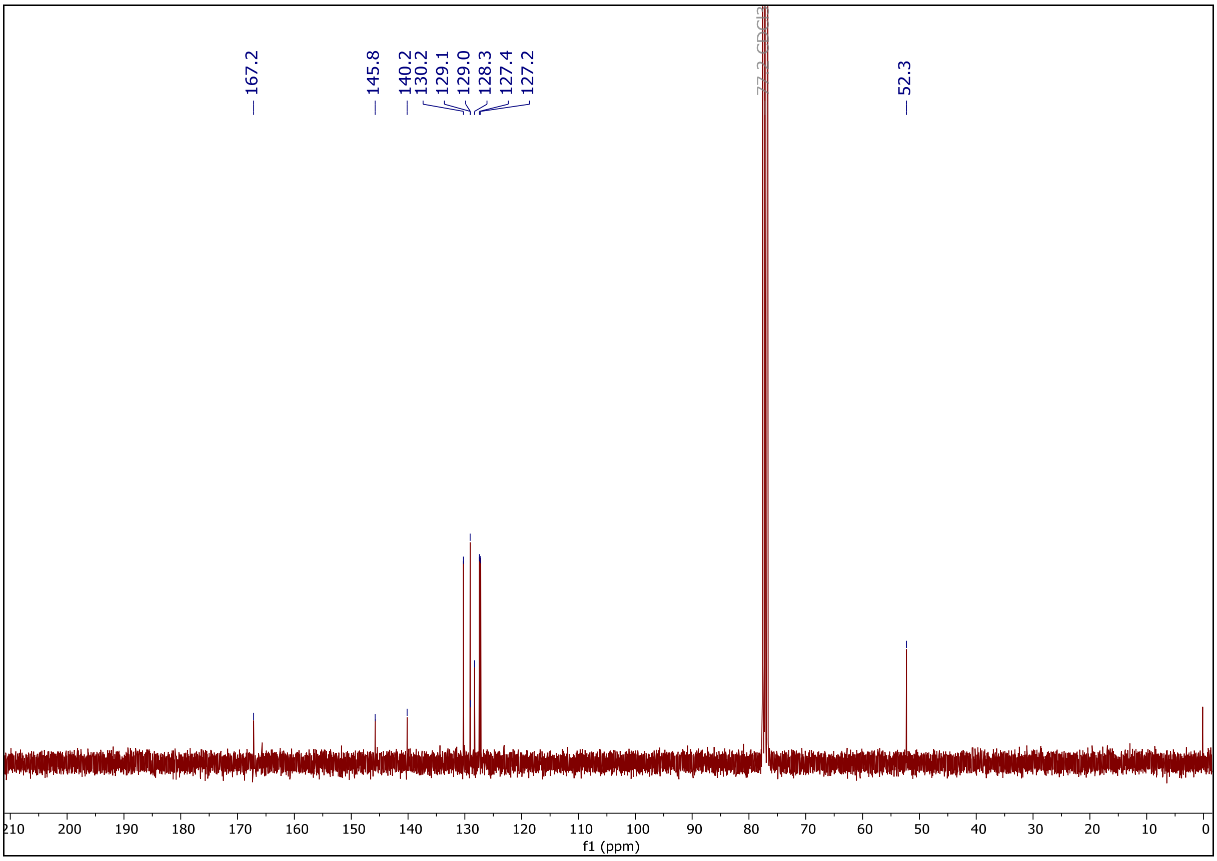

^{13}C-NMR (75 MHz, $CDCl_3$) δ 167.2, 145.8, 140.2, 130.2 (2C), 129.1 (2C), 129.0, 128.3, 127.4 (2C), 127.2 (2C), 52.3.

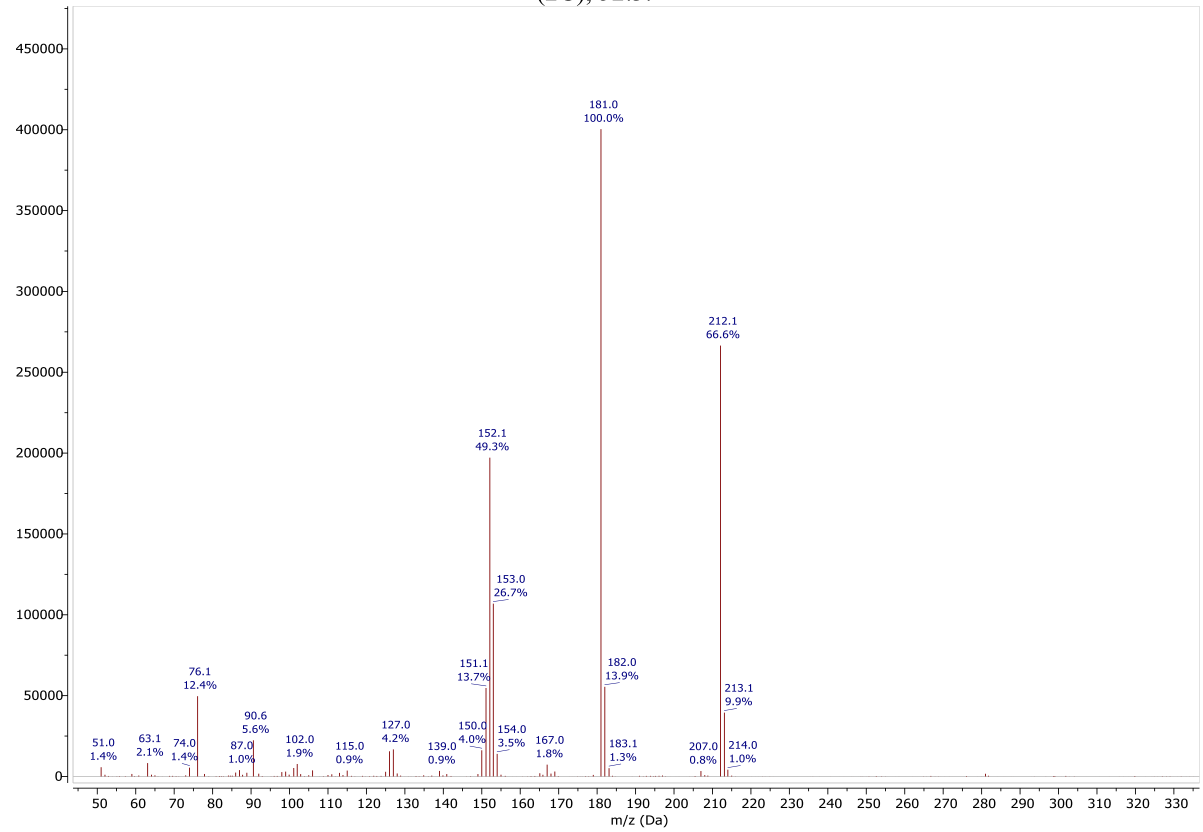

EI-MS: m/z calcd. for methyl [1,1'-biphenyl]-4-carboxylate [M]+ : 212.1, found 212.1.

<u>(E)-N-hexylnon-2-enamide</u>

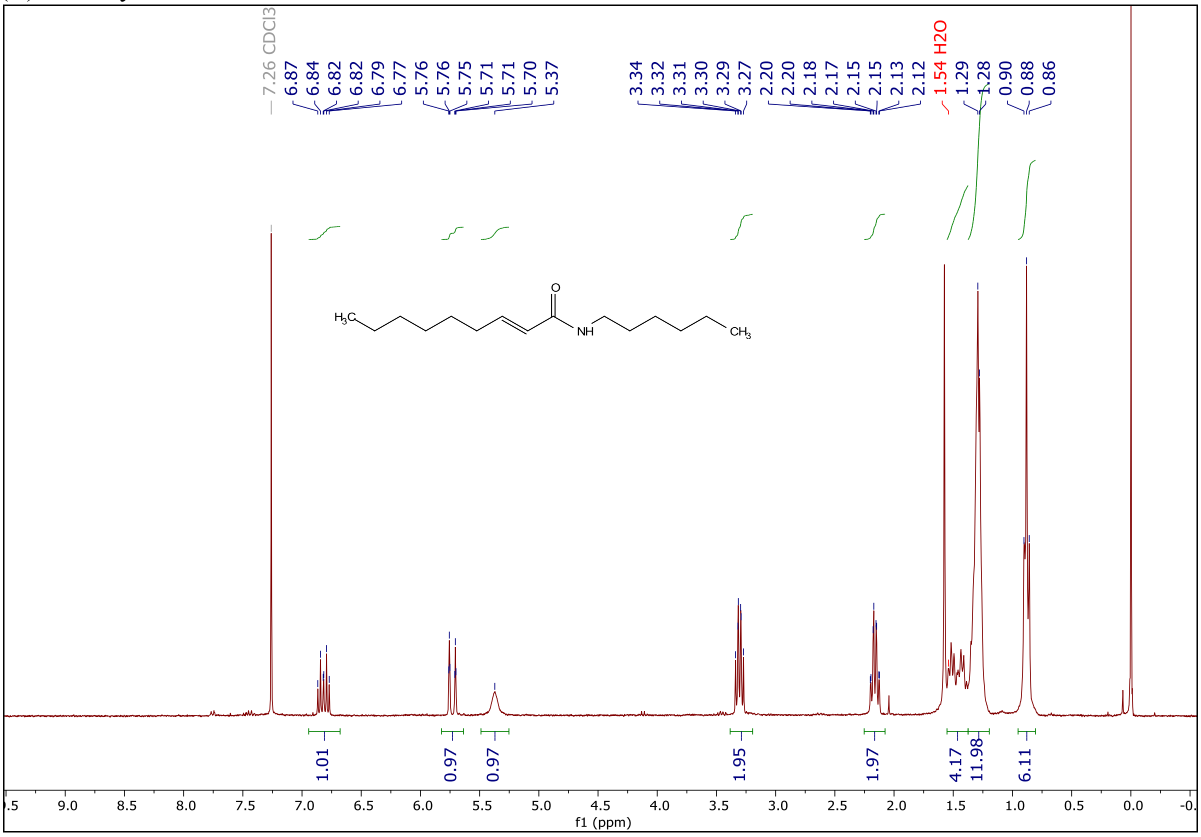

¹H-NMR (300 MHz, $CDCl_3$) δ 6.82 (dt, *J* = 15.3, 6.9 Hz, 1H), 5.73 (dt, *J* = 15.3, 1.6 Hz, 1H), 5.37 (bs, 1H), 3.31 (td, *J* = 7.2, 5.8 Hz, 2H), 2.16 (qd, *J* = 7.1, 1.6 Hz, 2H), 1.55-138 (m, 4H), 1.37-1.23 (m, 12 H), 0.95 – 0.81 (m, 6H).

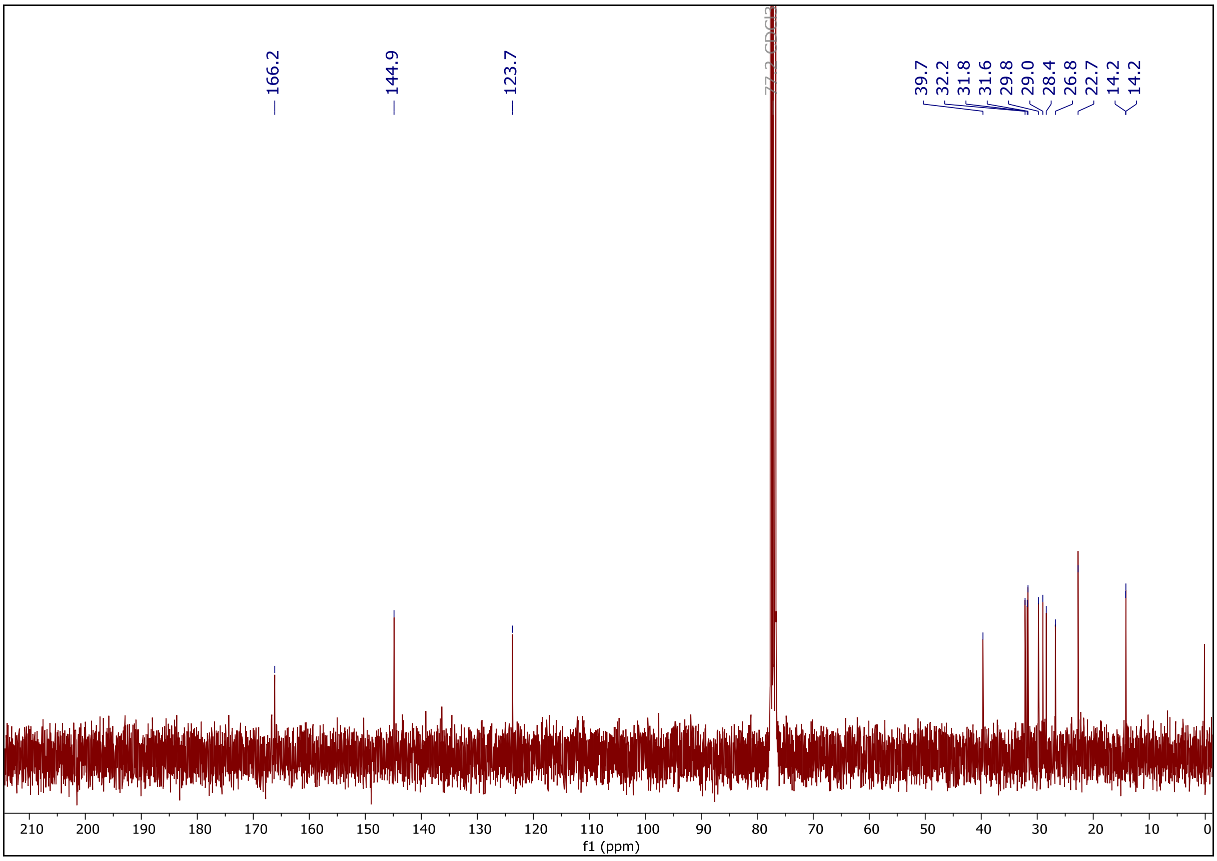

¹³C-NMR (75 MHz, $CDCl_3$) δ 166.2, 144.9, 123.7, 39.7, 32.2, 31.8, 31.6, 29.8, 29.0, 28.4, 26.8, 22.7 (2C), 14.2 (2C).

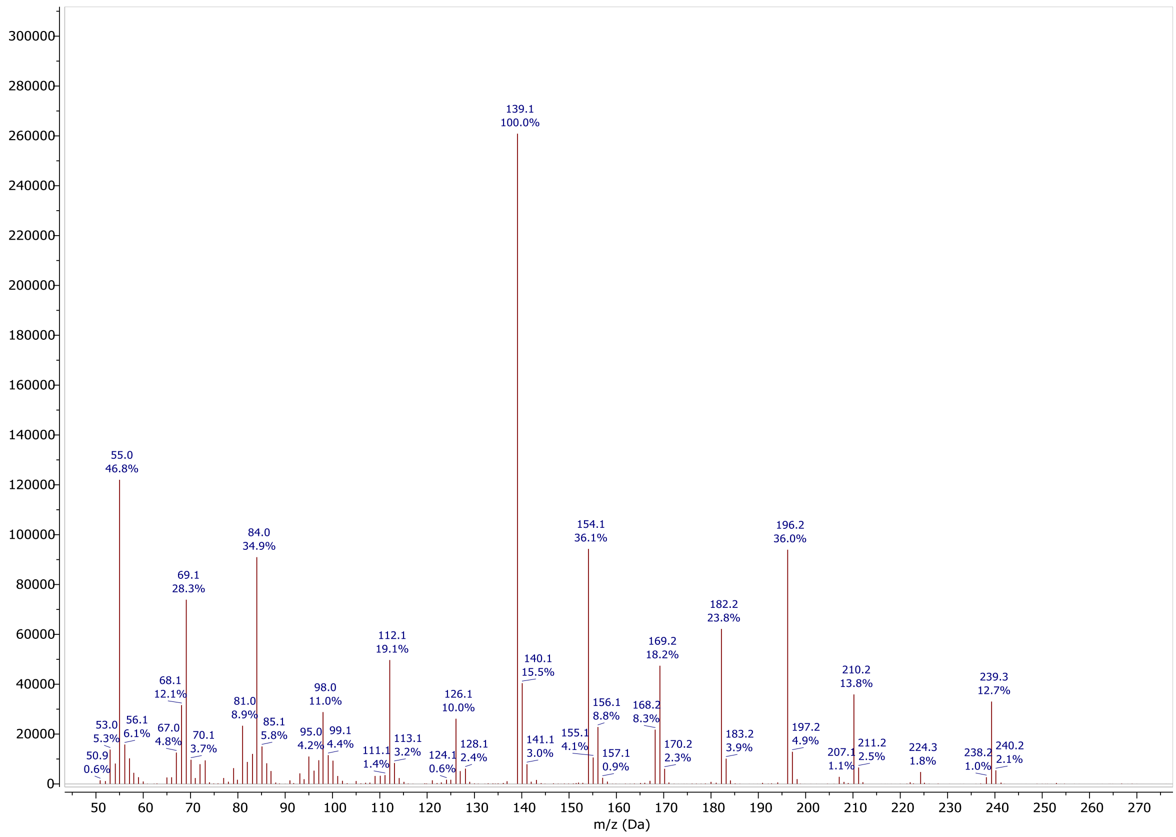

EI-MS: m/z calcd. for (E)-N-hexylnon-2-enamide [M]+ : 239.2, found 239.3
